# Guide RNAs containing universal bases enable Cas9/Cas12a recognition of polymorphic sequences

**DOI:** 10.1038/s41467-022-29202-x

**Published:** 2022-03-25

**Authors:** Amanda R. Krysler, Christopher R. Cromwell, Tommy Tu, Juan Jovel, Basil P. Hubbard

**Affiliations:** 1 Department of Pharmacology, University of Alberta, Edmonton, AB T6G 2R7 Canada; 2 The Applied Genomics Core, Office of Research, University of Alberta, Edmonton, AB T6G 2E1 Canada; 3 Department of Pharmacology and Toxicology, University of Toronto, Toronto, ON M5S 1A8 Canada

**Keywords:** Nucleic-acid therapeutics, Chemical modification, CRISPR-Cas9 genome editing

## Abstract

CRISPR/Cas complexes enable precise gene editing in a wide variety of organisms. While the rigid identification of DNA sequences by these systems minimizes the potential for off-target effects, it consequently poses a problem for the recognition of sequences containing naturally occurring polymorphisms. The presence of genetic variance such as single nucleotide polymorphisms (SNPs) in a gene sequence can compromise the on-target activity of CRISPR systems. Thus, when attempting to target multiple variants of a human gene, or evolved variants of a pathogen gene using a single guide RNA, more flexibility is desirable. Here, we demonstrate that Cas9 can tolerate the inclusion of universal bases in individual guide RNAs, enabling simultaneous targeting of polymorphic sequences. Crucially, we find that specificity is selectively degenerate at the site of universal base incorporation, and remains otherwise preserved. We demonstrate the applicability of this technology to targeting multiple naturally occurring human SNPs with individual guide RNAs and to the design of Cas12a/Cpf1-based DETECTR probes capable of identifying multiple evolved variants of the HIV protease gene. Our findings extend the targeting capabilities of CRISPR/Cas systems beyond their canonical spacer sequences and highlight a use of natural and synthetic universal bases.

## Abstract (web summary)

Genetic variance poses a major challenge for CRISPR-based human therapeutics and pathogen diagnostics. Here the authors show how to circumvent this issue by using guide RNAs containing universal bases to tailor Cas9/Cas12 specificity.

## Introduction

Clustered Regularly Interspaced Short Palindromic Repeat (CRISPR) systems play an important role in mediating adaptive immunity in prokaryotes^1^ and have been effectively repurposed for gene- and RNA-editing applications^2–5^. Over 400 different CRISPR-Cas homologs, comprised of diverse enzymes with unique nucleic acid binding specificities and cleavage mechanisms have been annotated^6^. These have been grouped into two distinct classes^7^. Class I systems employ multi-subunit nuclease complexes, while Class II systems, more widely used for gene editing, rely on a single effector protein^7^. Class II systems may be further divided into subtypes (e.g., II-A, II-B, V, VI) based on architecture^7^. Cas9, a type II-A system, directs DNA cleavage using two separately expressed RNA elements: a CRISPR RNA (crRNA) that contains a 20-nucleotide (nt) sequence complementary to the target DNA sequence, and a trans-activating crRNA (tracrRNA) that bridges the Cas9-crRNA interaction^8^. Target recognition by Cas9 involves binding a protospacer adjacent motif (PAM) sequence (5ʹ-NGG-ʹ3 in *S. pyogenes*), followed by hybridization of the 20-nt spacer sequence to the target^9^. Formation of a fully paired duplex induces conformational changes in the RuvC and HNH nuclease domains in Cas9 that ultimately result in a double-strand DNA cleavage event (Supplementary Fig. 1a)^9–11^. In contrast, Cas12a (Cpf1), which is a Class II Type V system, employs a single RuvC active site to induce staggered cuts within the target and non-target strands (Supplementary Fig. 1b)^7^. Cas12a recognizes a T-rich PAM (5′-TTN-′3), uses a 20–24 base-pair (bp) spacer sequence, does not require a tracrRNA, and has the ability to process its own pre-crRNA^12^. Unlike Cas9, Cas12a unleashes indiscriminate single-stranded DNase activity (collateral or *trans* activity) in vitro upon nuclease activation^13^.

Both Cas9 and Cas12a have been used to edit the genomes of numerous organisms ranging from plants to mammals^14,15^. Moreover, both systems have demonstrated potential for use in the context of clinical therapeutics to treat human genetic disease^14,15^. Furthermore, the collateral DNase activity of Cas12a has been exploited to generate a diagnostic platform for the detection of aberrant mutations or pathogen DNA sequences^13^. Briefly, the DNA endonuclease-targeted CRISPR trans reporter (DETECTR) system links activation of Cas12a nuclease activity to *trans* cleavage of a single-stranded DNA (ssDNA) substrate containing flanking fluorophore and quencher moieties^13^. When combined with isothermal amplification, this system achieves attomolar DNA detection sensitivity^13^.

One of the primary obstacles to translating CRISPR/Cas systems to clinical applications has been concern over off-target DNA cleavage, which could have detrimental health consequences for therapeutics, and yield false-positive results for diagnostics^16^. As a result, much work has been done to improve the specificity of these systems through protein engineering or evolution^17,18^, or engineering or chemical modification of guide RNAs^11^. For example, guide RNAs with engineered secondary structures improve Cas12a specificity^19^, and incorporation of DNA^20^ or bridged nucleic acids (BNA)^11^ into Cas9 gRNAs improves its specificity.

While single-nt precision is desirable for many nucleic acid targeting applications^19^, there are other instances where recognition of a discrete 20 bp sequence may be limiting. First, CRISPR/Cas9 can be sensitive to naturally occurring SNPs within the PAM-proximal portion of a guide sequence^21–23^. Since SNPs occur roughly every 300 bp in the human genome^24^, a CRISPR/Cas9 therapeutic designed for one patient may be ineffective for another. Indeed, a recent test of 263 therapeutically-relevant guide RNAs revealed that >16% failed to cleave the on-target site in at least one of 7700 haplotypes tested^25^. Second, the high degree of natural genetic diversity present in pathogens such as HIV-1 greatly complicates antiviral treatment or diagnostic detection using CRISPR/Cas systems^26,27^. Finally, studies have shown that even successful cleavage of HIV-1 DNA sequences using CRISPR/Cas9 can result in mutations that accelerate viral escape and render the virus resistant to the original guide RNA^28,29^. These scenarios highlight the need for additional CRISPR/Cas capabilities that allow sequences to be targeted in a more flexible manner.

**Fig. 1:**
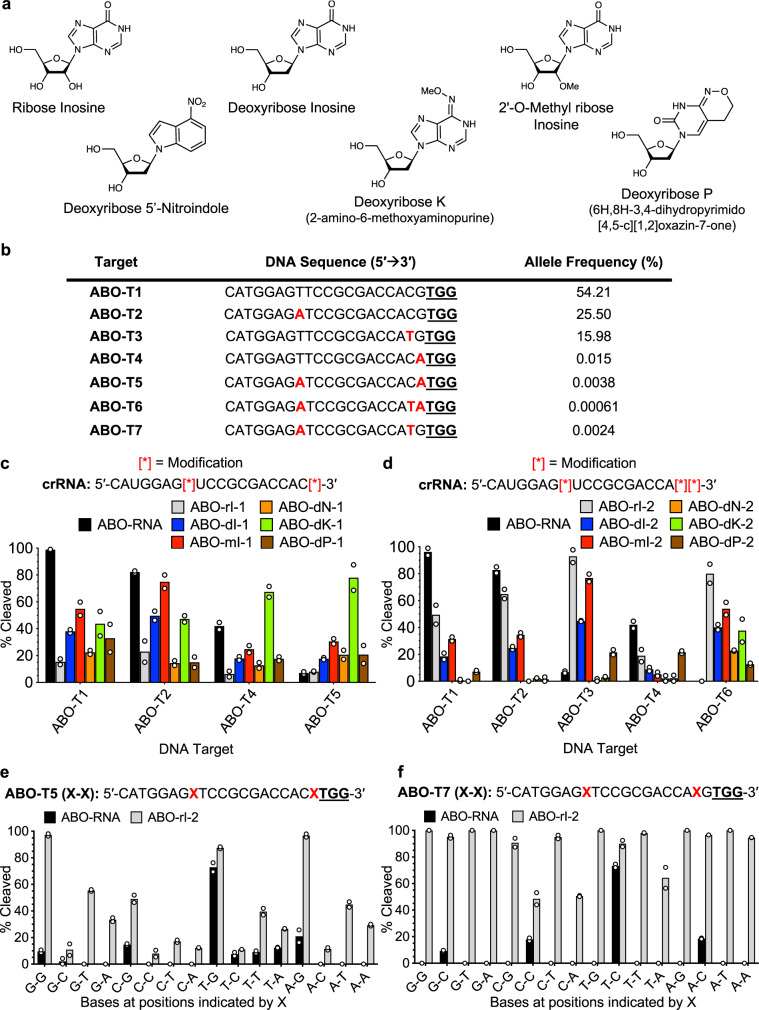
Incorporation of universal bases into Cas9 crRNAs enables the targeting of polymorphic gene variants. **a** Chemical structures of universal and degenerate bases used in this study. **b** List of DNA targets corresponding to sequences in the *ABO* gene based on clinical polymorphism data. SNPs are indicated with red lettering. Allele frequency indicates either the current tallied allele frequency or the statistically predicted frequency (for sequences containing multiple SNPs). The PAM sequence is underlined. Bar graphs showing the relative amount of DNA cleavage resulting from in vitro reactions containing Cas9 with (**c**) double or (**d**) triple modified-crRNAs and the variant DNA target sequences indicated. Locations of the universal bases in the crRNA sequence are indicated with red [*]. rI = Ribose Inosine, dI = Deoxyribose Inosine, mI = 2′-O-Methyl ribose Inosine, dN = Deoxyribose 5′-Nitroindole, dK = Deoxyribose K, dP = Deoxyribose P; Mean with individual data points shown (*n* = 2 independent experiments). Bar graphs showing the relative amount of DNA cleavage resulting from in vitro reactions containing Cas9 with ABO-RNA or ABO-rI-2 versus derivatives of the (**e**) ABO-T5 or (**f**) ABO-T7 target sequences. Base combinations listed along the *x*-axis correspond to the positions indicated by a red ‘X’ in the reference sequence. The PAM sequence is underlined; Mean with individual data points shown (*n* = 2 independent experiments). Reactions were performed using fixed concentrations of gRNA (80 nM) and Cas9 (40 nM). Cleavage percentages were calculated from corresponding agarose gels using densitometry software (ImageJ).

In nature, recognition of degenerate mRNA codons by the tRNA anticodon loop is achieved through the inclusion of ribose inosine (I) nts (containing the hypoxanthine base) (Fig. 1a)^30,31^. Inosine also plays a role in RNA editing^32^ and acts as a DNA damage intermediate following adenosine deamination^33^. Characterized as a ‘universal base’, inosine forms 2 hydrogen bonds with all four canonical bases with a slight I-C > I-A > I-T ≈ I-G bias in stability^30^. Inosine has been successfully applied to the design of degenerate PCR primers and diagnostic probes, as well as in DNA sequencing^30^. It can be incorporated into a nucleic acid strand either as a standard RNA or DNA nt, or as modified variant such as 2′-O-methyl (2′ OMe) RNA, which displays improved nuclease resistance and unique hybridization properties^34^. Synthetic universal bases such as deoxyribose 5′-nitroindole (Fig. 1a) have also been developed^35^. This base lacks the ability to form any hydrogen bonds but adopts a standard *anti* configuration with the opposing nt and acts to stabilize hydrophobic base stacking^36^. While more destabilizing in certain contexts, 5′-nitroindole bases appear to be devoid of any base-pairing bias^36,37^. Other synthetic bases have been developed to exhibit partial degeneracy, including deoxyribose K (2-amino-6-methoxyaminopurine) and deoxyribose P (6H,8H-3,4-dihydro-pyrimido[4,5-c] [4,5-c] [1,2]oxazin-7-one) (Fig. 1a), which show a preference for C/T and A/G pairing, respectively^38,39^.

Based on their present use in nucleic acid amplification and detection technologies^30^, we hypothesized that universal bases could be harnessed to impart Cas systems with the ability to target multiple polymorphic sequences using an individual guide RNA. Here, we show that multiple types of chemically distinct universal bases can be tolerated within both Cas9 and Cas12a guide RNAs, in a context-dependent manner. We show that single crRNAs containing universal bases can be used to target multiple gene variants containing naturally occurring SNPs (Supplementary Fig. 1c). Furthermore, we design individual probes containing universal bases that are capable of identifying multiple variants of the HIV-1 protease gene using the DETECTR system (Supplementary Fig. 1d). Using high-throughput specificity profiling, we show that inclusion of universal bases imparts selective degeneracy at the site of incorporation, without otherwise altering crRNA specificity. Our results outline a new strategy for expanding the capabilities of CRISPR/Cas to the recognition of nucleic acid targets with high variability and those for which only incomplete sequence information is available.

## Results

### Incorporation of universal bases into Cas9 crRNAs enables targeting of polymorphic sequences

Past studies have shown that the inclusion of sugar^11,40^ and backbone^41,42^ chemical modifications in Cas9 crRNAs can be tolerated. In addition, crRNAs containing locked/bridged nucleic acids (LNA/BNA) and DNA have been demonstrated to reduce Cas9 off-target DNA cleavage activity relative to their unmodified counterparts^11,40^. Given these findings, we speculated that incorporation of non-canonical bases into crRNAs might also be permitted. In particular, we wondered if universal bases could be incorporated into crRNAs so as to enable Cas9 recognition of polymorphic target sequences. To test this possibility, we selected a highly polymorphic sequence from the *ABO* gene that determines the most clinically important blood group system in mammals^43^. We generated a series of 16 DNA target sequences (ABO-T1–16), derived from prevalent alleles in the human population, containing naturally occurring single nucleotide polymorphisms (SNPs) within that region (Fig. 1b, Supplementary Fig. 2a). Next, we tested the ability of Cas9 to cleave these sequences in vitro using an unmodified guide RNA (ABO-RNA) corresponding to the reference sequence (ABO-T1). Consistent with previous studies on Cas9 specificity^11,44^, we observed robust cleavage of the on-target sequence (ABO-T1) and two sequences containing single SNPs (ABO-T2, ABO-T4), but weak or absent activity on all of the other sequence variants (Supplementary Fig. 2b). These results reinforce the negative impact that natural genetic variation can have on Cas9 on-target activity.

To generate guide RNAs capable of simultaneously recognizing a broader set of *ABO* sequence variants, we selected two *ABO* sequences bearing 2 (ABO-T5) or 3 (ABO-T6) polymorphisms relative to ABO-T1 (Fig. 1b), and designed a panel of corresponding crRNAs in which ribose inosine (ABO-rI-1, ABO-rI-2), deoxyribose inosine (ABO-dI-1, ABO-dI-2), 2′OMe ribose inosine (ABO-mI-1, ABO-mI-2), deoxyribose 5′-nitroindole (ABO-dN-1, ABO-dN-2), deoxyribose K (ABO-dK-1, ABO-dK-2), or deoxyribose P (ABO-dP-1, ABO-dP-2) bases were substituted at positions overlapping with the SNPs (Fig. 1c, d). Using ABO-rI-1, ABO-dI-1, ABO-mI-1, ABO-dN-1, ABO-dK-1, and ABO-dP-1, we assayed Cas9 cleavage activity on ABO-T1, the corresponding ABO-T5 double SNP variant sequence, and sequences containing each SNP in isolation (ABO-T2, ABO-T4). Using the ABO-mI-1 and ABO-dK-1 crRNAs, Cas9 cleaved ABO-T5 > 5 and >10-fold more abundantly than with ABO-RNA, respectively (Fig. 1c, Supplementary Fig. 3a, b). Both of these crRNAs also supported Cas9 cleavage of the single variant (ABO-T2, ABO-T4) and reference (ABO-T1) sequences (Fig. 1c). Similarly, we found that ABO-rI-2, ABO-dI-2, ABO-mI-2, and ABO-dK-2 guided efficient Cas9 cleavage of ABO-T6 ( > 50% compared to 0% with ABO-RNA) (Fig. 1d, Supplementary Fig. 3a, c). ABO-rI-2 and ABO-mI-2 were also able to direct the cleavage of ABO-RNA and the single variant sequences ABO-T2 and ABO-T3, but not ABO-T4 (Fig. 1d). These results demonstrate that universal bases with diverse chemistries can be incorporated into crRNAs to allow simultaneous targeting of complex SNP variants in vitro.

Our findings in Fig. 1c and Fig. d indicated that amongst the various universal bases we tested, inosine derivatives (ribose, deoxy, 2′OMe) appeared to be the most consistently well tolerated in vitro. Therefore, we chose to focus our studies on this naturally occurring non-canonical base. Unlike synthetic bases such as deoxyribose 5′-nitroindole, previous work has shown that inosine exhibits a slight base pairing preference in certain contexts^30^. We wondered if a base pairing bias might manifest in our in vitro Cas9 DNA cleavage reactions. To test this, we designed two sets of 16 target sequences covering all combinations of bases at the two SNP locations in ABO-T5 and ABO-T7, and evaluated cleavage of these sequences by Cas9 using either ABO-rI-2 or the unmodified crRNA. As shown in Fig. 1e, Cas9 was able to cut 9 of the 16 targets with >25% efficiency using ABO-rI-2 (the remaining seven sequences were also cut at low levels), compared to only the reference sequence being cleaved to this extent using the unmodified crRNA. The results using the ABO-T7 derivative sequences were even more striking. All 16 of the derivative sequences were cleaved at >50% efficiency by Cas9 using ABO-rI-2, while only the reference sequence was cut at appreciable levels using the unmodified crRNA (three other sequences were cleaved at lower levels). These results suggest that incorporation of inosine bases into crRNAs enables targeting of all four canonical bases at the corresponding DNA target sites in a relatively unbiased and independent manner.

To characterize the patterns of inosine modifications permitted by Cas9, we synthesized an additional 13 crRNAs containing 1–4 ribose inosine modifications (Supplementary Fig. 4a) and tested the ability of these to direct cleavage of the ABO-T1 sequence by Cas9. We found that inclusion of a single inosine was tolerated in all instances, albeit with reduced activity, while crRNAs containing 2–4 inosine substitutions supported Cas9 cleavage of ABO-T1 in certain cases (Supplementary Fig. 4b). Next, we sought to determine the cause of the reduced activity and to establish if the effect was general or target-specific. Using ABO-RNA, and two crRNAs containing two inosine modifications, ABO-rI-1 (low activity) and ABO-rI-8 (no activity on the ABO-T1 sequence using the given conditions), we performed a titration of tracrRNA:crRNA to determine if inosines within the spacer sequence might somehow impair the ability of these two RNA elements to hybridize. Altering this ratio did not result in increased activity, ruling out this possibility (Supplementary Fig. 5a, b). In addition, the low cleavage activity observed in vitro using ABO-rI-1 or ABO-rI-8 could not be augmented by increasing reaction time (Supplementary Fig. 5c). Based on these results, we hypothesized that the lowered activity observed using certain inosine-modified crRNAs may be due to decreased ribonucleoprotein (RNP) complex binding to the target DNA sequence. A titration of activity versus RNP concentration provided evidence to support this assertion (Supplementary Fig. 5d). Moreover, data from electrophoretic mobility shift assays (EMSAs) confirmed that RNP binding to ABO-T1 was substantially reduced using ABO-rI-1 and ABO-rI-8 compared to the unmodified crRNA (Supplementary Fig. 6). Interestingly, we found that ABO-rI-8, which did not support Cas9 cleavage of ABO-T1, did support cleavage of ABO-T7, establishing its activity on other target sequences (Supplementary Fig. 7). In all instances, we observed a strong correlation between RNP-target engagement and activity in DNA cleavage assays (Supplementary Figs. 6, 7). Previous work has shown that I-G and I-A pairs decrease thermodynamic duplex stability by 0.84 kcal/mol and 0.52 kcal/mol compared to C-G and A-T pairs, respectively^30^. We found that in the absence of Cas9, T_m_ values for inosine-modified crRNA-target DNA duplexes were in fact reduced compared to the unmodified counterpart (Supplementary Fig. 8). Thus, it is likely that incorporation of inosines into crRNAs destabilizes Cas9-DNA target binding, although the extent to which this affects overall activity appears to be context-dependent and minimal in some cases.

### Inclusion of universal bases into crRNAs alters the specificity only at the site of incorporation

**Fig. 2:**
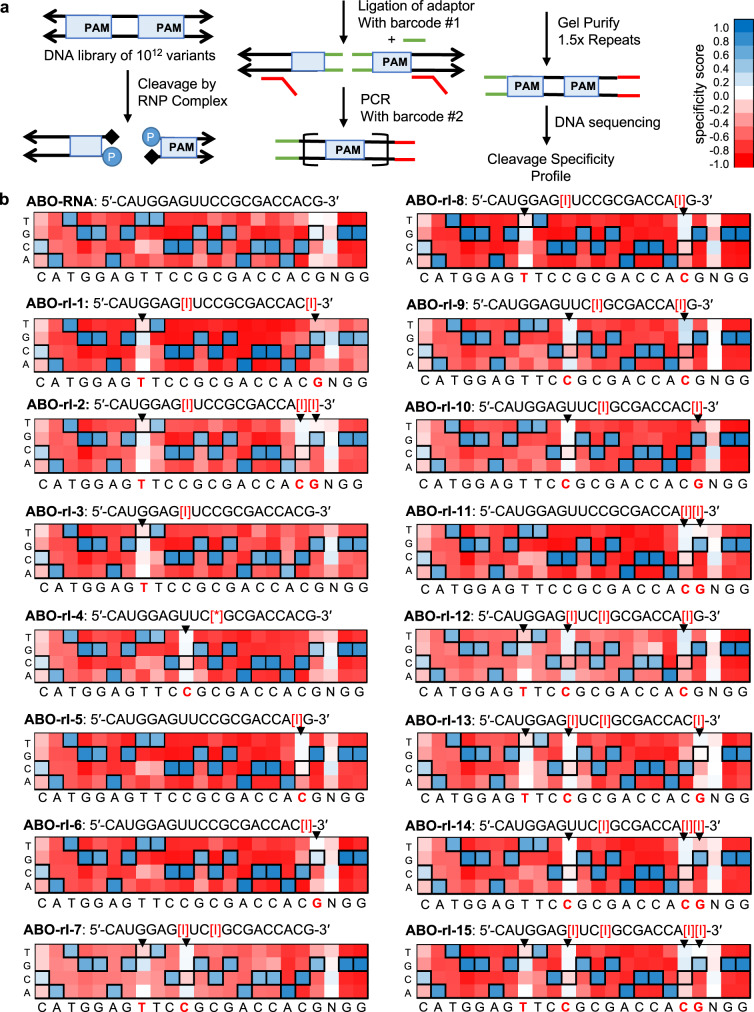
Inclusion of inosine bases in crRNAs affects specificity mainly at the site of incorporation. **a** Diagram depicting the workflow for the high-throughput specificity profiling assay. **b** Heatmaps corresponding to the specificity profiles of the indicated ribose inosine-modified crRNAs. The positions of inosine bases are indicated by black arrows. Specificity scores of 1.0 (dark blue) correspond to 100% enrichment for, while scores of −1.0 (dark red) correspond to 100% enrichment against a base-pair at a specific position. Black boxes denote the intended target nucleotide.

A prerequisite for the practical application of guide RNAs containing universal bases to targeting SNPs is that they must alter Cas9 specificity in a localized and predictable manner. That is to say, they should impart selective degeneracy rather than globally impacting the precision of Cas9 DNA cleavage. To evaluate this, we employed a previously described high-throughput specificity profiling assay^11,16,45^ that measures Cas9 cleavage of a library of >10^12^ off-target sequences, containing a tenfold coverage of all sequences with ≤8 mutations relative to the ABO-T1 sequence (Fig. 2a). We performed the assay on the unmodified crRNA and all 15 of the ribose inosine-modified crRNAs listed in Supplementary Fig. 4a, as well as all of the crRNAs modified using alternative universal bases listed in Fig. 1c, d. We used the datasets for each crRNA to calculate enrichment scores for each base at each position within the ABO-T1 sequence and generated specificity heatmaps to visualize the results. For the collection of inosine-modified crRNAs, we found that in nearly all cases the specificity profile for the crRNAs containing universal bases was similar to that of ABO-RNA at all positions except those that overlapped with the locations of the universal bases (Fig. 2b, Supplementary Figs. 9–11). Moreover, substitution of the indicated bases with inosine rendered the crRNA virtually non-specific at that position (Fig. 2b), and was associated with changes in specificity scores ranging from approximately −0.6 to −1.0 at those sites (Supplementary Fig. 10). Similar results were observed from the analysis of the crRNAs bearing deoxyribose inosine, 2′OMe ribose inosine, deoxyribose 5′-nitroindole, deoxyribose K and deoxyribose P base modifications (Fig. 2, Supplementary Figs. 12–15). Overall, we found that specificity at the site of universal base incorporation was virtually abolished, while specificity at other locations appeared to be preserved, or even enhanced in certain cases (Supplementary Fig. 13). Substitution of the indicated PAM-distal uracil with ribose inosine (ABO-rI-1 and ABO-rI-2), deoxyribose inosine (ABO-dI-1 and ABO-dI-2), or 2′O methyl inosine (RNA-mI-1 and RNA-mI-2) rendered the crRNA non-specific at this position (Supplementary Fig. 13) and was associated with a difference in specificity score in excess of −0.6 (Supplementary Fig. 14). Similar results were observed when the indicated PAM-proximal cytosine base was replaced by a universal base, while specificity at the PAM-proximal guanine position was less affected, ostensibly due to an initial lack of specificity at this position in ABO-RNA (Supplementary Figs. 13, 14). Finally, to generalize our findings to other DNA target sequences, we synthesized a separate set of 8 crRNAs with inosine modifications at positions corresponding to SNPs present in a region of the major histocompatibility complex *HLA* gene. As shown in Supplementary Figs. 16–19, inclusion of inosine bases in this crRNA similarly abolished specificity in a site-restricted manner. Collectively, these data reveal that inclusion of universal bases in crRNAs imparts selective degeneracy at the site of incorporation without otherwise altering specificity, and that this effect extends to compositionally distinct DNA targets.

### crRNAs containing universal bases can direct Cas9 cleavage of polymorphic sequences in cells, but with limitations

**Fig. 3:**
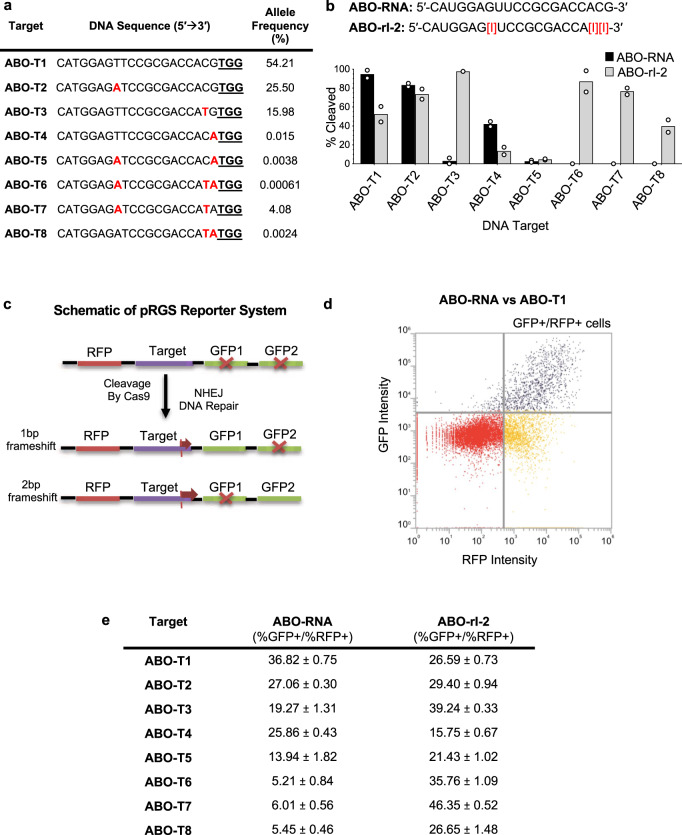
crRNAs containing inosine bases direct simultaneous cleavage of polymorphic gene variants in cells. **a** List of *ABO* variant DNA target sequences (ABO-T1-T8) assayed in cells. Positions of SNPs are indicated with red lettering. The PAM sequence is underlined. **b** Bar graphs showing the relative amount of DNA cleavage resulting from in vitro reactions containing Cas9 with ABO-RNA or ABO-rI-2 versus the indicated DNA target sequences. Assays were performed using fixed concentrations of gRNA (80 nM) and Cas9 (40 nM); Mean with individual data points shown (*n* = 2 independent experiments). **c** Schematic outlining the framework for the fluorescence-based assay used to evaluate cleavage of the *ABO* variant target sequences in cells. **d** Representative FACS plot showing the distribution of RFP and GFP positive cells. Dual positive cells appear in the top right quadrant. **e** Table showing normalized %GFP + /all %RFP + events corresponding to cleavage of the indicated target sequences in cells by Cas9 using either ABO-RNA or ABO-rI-2; Mean ± S.D. shown (*n* = 3 independent samples).

Knowing that inclusion of universal bases in crRNAs could impart selective degeneracy while broadly maintaining cleavage specificity in vitro, we sought to determine if our results could be translated to cells. As an initial test, we adapted a plasmid-based fluorescence reporter system^46^ and used it to measure the cleavage of eight heterologous *ABO* sequences in cells. First, we selected ABO-rI-2, which bears three inosine modifications, and tested its ability to direct Cas9 cleavage of ABO-T1, the corresponding triple SNP variant (ABO-T6), three double SNP sequences (ABO-T5, 7, 8) and three single SNP sequences (ABO-T2, T3, T4) in vitro (Fig. 3a). We found ABO-rI-2 directed >50% cleavage of 6/8 sequences tested, the exceptions being ABO-T4 (~20%) and ABO-T5 (<10%) (Fig. 3b). In contrast, ABO-RNA only supported robust Cas9 cleavage of >50% of its matched sequence (ABO-T1) and ABO-T2 (Fig. 3b). Next, we cloned all of the target DNAs into a plasmid in which sequences were flanked by an in-frame mRFP gene at the 5′ end and two out-of-frame eGFP genes at the 3′ end (Fig. 3c). Past work has shown that double-strand breaks formed in the intervening target sequence can be repaired by non-homologous end-joining (NHEJ), resulting in frameshift mutations that generate a multifluorescent mRFP-eGFP fusion protein (Fig. 3c)^46^. We co-transfected all eight constructs with either ABO-RNA or ABO-rI-2 into HeLa cells stably expressing Cas9 and used fluorescence-activated cell sorting (FACS) to quantify the resulting cell populations (Fig. 3d). Using the ABO-RNA, Cas9 cleaved 3/8 sequences with >20% efficiency (ABO-T1, ABO-T2, ABO-T4). However, 7/8 sequences were cleaved with >20% efficiency, and ABO-T4 RNA was cleaved at 16% efficiency when the ABO-rI-2 guide RNA was used (Fig. 3e).

To examine the utility of this approach for targeting endogenous sites in cells, we sequenced several loci containing PAM sequences in 293 T and HeLa cells that were predicted to contain SNPs based on the Ensembl^47^ and HEK293T^48^ reference genomes. We identified a homozygous sequence within the *HLA-C* gene differing at 2 base positions between 293 T and HeLa cells (Supplementary Fig. 20a). We generated a crRNA corresponding to the *HLA-C* sequence in 293 T cells (HLA-C-RNA), and verified its ability to direct Cas9 cleavage of the *HLA-C* gene in 293 T cells (HLA-C-T1) but not HeLa cells (HLA-C-T2) (Supplementary Fig. 20b). We then synthesized crRNAs containing ribose inosine, deoxyribose inosine, or deoxyribose P bases at positions overlapping with the locations of the mismatches in the HLA-C-T1 and HLA-C-T2 sequences (Supplementary Fig. 20c). We tested the ability of these universal base-modified crRNAs and the unmodified crRNA to direct Cas9 cleavage of the two target sequences in vitro. We found that Cas9 was able to robustly cut both the HeLa and 293 T *HLA-C* sequences when the rI, dI, and dP base-modified crRNAs were used (Supplementary Fig. 20d). The unmodified crRNA induced ~60% cleavage of its corresponding target (HLA-C-T1), but only ~30% when HLA-C-T2 was used as a substrate (Supplementary Fig. 20d). The fact that Cas9 cleavage of this off-target sequence was absent in cells using the unmodified crRNA is consistent with previous reports in literature showing higher stringency against off-target cutting in cells^11,40,49^. Finally, we tested the ability of the unmodified and the rI-, dI-, and dP-modified crRNAs to direct Cas9 cleavage of the *HLA-C* locus in 293 T and HeLa cells. As previously observed, we found that the HLA-C-RNA supported Cas9 cleavage of the *HLA-C* locus in 293 T cells (~40%) but not HeLa cells (0%) (Supplementary Fig. 20e). In contrast to our in vitro findings, HLA-C-rI and HLA-C-dI showed either weak or undetectable Cas9 cleavage activity in both 293 T and HeLa cells. However, HLA-C-dP was able to direct Cas9 cleavage of the *HLA-C* locus in both 293T (~40%) and HeLa cells (~12%). Collectively, these results demonstrate the potential for universal base-modified crRNAs to drive Cas9 cleavage of polymorphic sequences in cells, but also reveal some limitations to their general use.

We wondered if the discrepancy between the activity of the HLA-C-rI and HLA-C-dI crRNAs in vitro and in cells could be the result of delayed Cas9 cleavage kinetics. Previous work has shown that modification of the ribose sugar in crRNAs can lead to slower enzyme kinetics that manifests as reduced activity in cells^11^. To test this hypothesis, we performed a Cas9 cleavage time course on DNA substrates corresponding to either HLA-C-T1 or HLA-C-T2 using HLA-C-RNA or HLA-C-rI, -dI, or -dP crRNAs. As shown in Supplementary Fig. 21, we found that Cas9 cleavage of HLA-C-T1 using HLA-C-rI or HLA-C-dI crRNAs was slower than with HLA-C-RNA or HLA-C-dP by a factor of ~4 fold. Furthermore, we found that Cas9 cleavage of the HLA-C-T2 substrate using HLA-C-dP was substantially quicker than cleavage using the HLA-C-RNA, -rI, and -dI crRNAs (Supplementary Fig. 21). This strong correlation between cellular modification rates and in vitro kinetics suggests that delayed enzyme kinetics could underlie the low activity of the HLA-C-rI and HLA-C-dI crRNAs in cells.

### DETECTR probes containing universal bases identify evolved variants of a pathogen

**Fig. 4:**
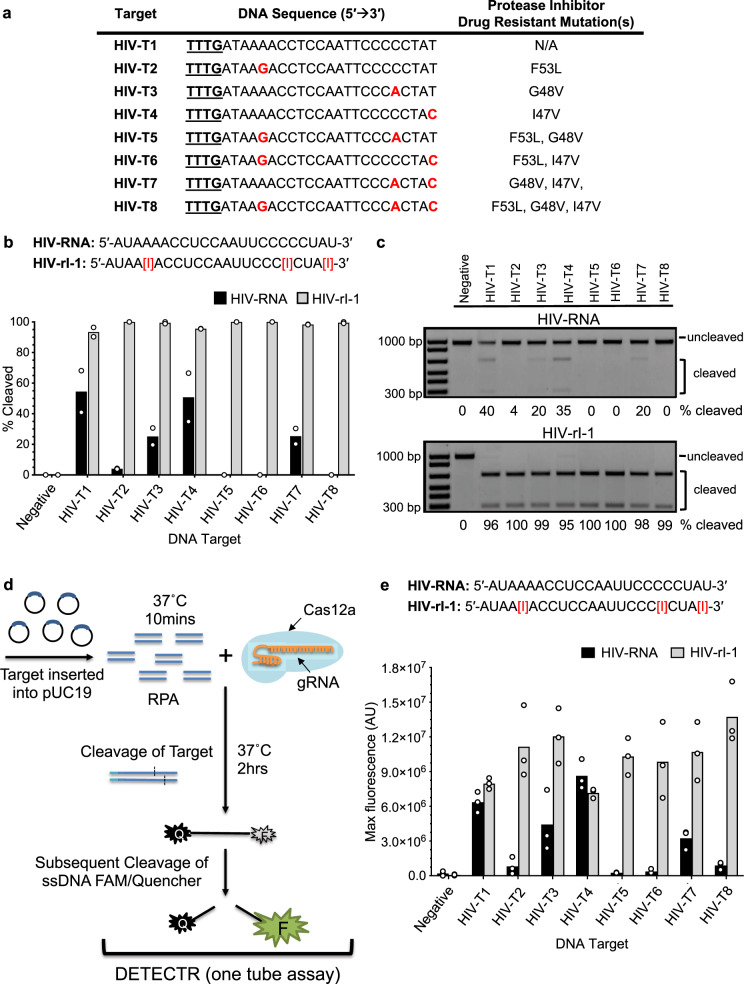
Incorporation of inosine bases into crRNA probes for the Cas12a-based DETECTR system enables the detection of evolved HIV-1 DNA target sequences. **a** List of DNA target sequences derived from the HIV-1 protease gene containing evolved SNPs detected in patient samples. SNPs position(s) are indicated with red lettering. The PAM sequence is underlined. **b** Bar graphs showing the relative amount of DNA cleavage resulting from in vitro reactions containing Cas9 with HIV-RNA or HIV-rI-1 versus the indicated DNA target sequences. A scrambled crRNA with the sequence 5′-AUUCUUGCUCUGCUCUCUUCGUC-′3 was used as a negative control. Assays were performed using fixed concentrations of crRNA (125 nM) and Cas12a (100 nM); Mean with individual data points shown (*n* = 2 independent experiments). **c** Representative gels of the in vitro cleavage assay results for Cas12a with HIV-RNA or HIV-rI-1 versus the indicated DNA target sequences. The bottom two bands in the gel represent the cleaved DNA substrate while the top band corresponds to the undigested substrate. Cleavage experiments were performed in duplicate with similar results. **d** Diagram outlining the DETECTR assay. **e** Bar graph indicating the fluorescence signal obtained in the DETECTR assay using Cas12a in combination with either HIV-RNA or HIV-rI-1 and samples containing the indicated target sequences. Max fluorescence values were normalized to background; Mean with individual data points shown (*n* = 3 independent experiments).

In addition to its use as a gene-editing agent, Cas12a/Cpf1 has also successfully been harnessed for diagnostic purposes as part of the DETECTR system^13^. Point-of-need technologies using this platform to diagnose swine flu^50^ as well as COVID-19^51^ have now been deployed. However, the prospect of viral evolution presents a unique challenge for the identification of these pathogens, as mutations could subvert detection by Cas12a guide probes designed to target only reference sequences, leading to false negative results. We hypothesized that inosine bases could be incorporated into Cas12a guide RNAs to impart them with selectively degenerate targeting capabilities in order to circumvent this limitation. To test this possibility, we selected a DNA sequence from the HIV-1 protease gene and identified seven clinically-relevant sequence variants bearing 1, 2, or 3 SNPs encoding mutations that confer resistance of the virus toward HIV protease inhibitor drugs^52,53^ (Fig. 4a). Next, we synthesized two crRNAs, HIV-RNA to target the canonical sequence, and HIV-rI-1, which contains three inosine substitutions designed to enable flexible targeting of both the canonical and evolved variant sequences. An in vitro cleavage assay of all target sequences using HIV-RNA with Cas12a revealed that HIV-T1, HIV-T3, HIV-T4, HIV-T7 were cleaved at efficiencies of 55%, 30%, 55%, and ~30%, respectively (Fig. 4b, c). In stark contrast, all eight sequences were fully cleaved when HIV-rI-1 was used as the guide RNA (Fig. 4b, c), supporting our assertion and revealing a high degree of tolerance for the presence of inosine substitutions in Cas12a guide RNAs. To ensure that the lack of cleavage activity observed with HIV-RNA on sequences such as HIV-T8 was not simply due to insufficient RNP, we performed titrations of RNP concentration. Consistent with our model, we found that overall Cas12a cleavage activity (combined *cis* and *trans*) was comparable between HIV-RNA and HIV-rI-1 using the HIV-T1 substrate (Supplementary Fig. 22a, b). However, HIV-RNA was unable to direct cleavage of HIV-T8, in contrast to HIV-rI-1, which induced complete cleavage of this substrate at an RNP concentration of ~25 nM (Supplementary Fig. 22c, d). Subsequently, we ported these probes into the DETECTR system, outlined in Fig. 4d. To simulate pathogen DNA, we cloned each of our eight target sequences into pUC19 plasmids and performed recombinant polymerase amplification (RPA) as described in the protocol^13^. Next, we set up individual reactions containing each DNA sample paired with either HIV-RNA or HIV-rI-1 probes in the presence of a fluorescent detection substrate. As shown in Fig. 4e, the HIV-rI-1 probe positively identified all eight of the HIV-1 variant sequences, while the HIV-RNA probe only identified three sequences and provided false negatives for the other five variants. These findings provide justification for the use of universal base-modified crRNAs in CRISPR-based diagnostic platforms.

Viral escape due to mutation of the target site to a variant is a major roadblock to using CRISPR therapeutics as antivirals^54^. Based on our results demonstrating effective targeting of polymorphic sequences using HIV-rI-1 in vitro, we wondered if this crRNA could direct Cas12a cleavage of variant viral sequences in cells. To test this possibility, we used the Flp/FRT system^55^ to stably integrate single copies of the HIV-T1 and HIV-T8 sequences into 293 cells (Supplementary Fig. 23a, b). We found that the unmodified HIV-RNA crRNA directed robust Cas12a cleavage of the HIV-T1 site (~28%) but virtually no cleavage at the HIV-T8 site (<3%) (Supplementary Fig. 23c). In contrast, the HIV-rI-1 RNA induced cleavage of both sites with relatively equal efficacy (HIV-T1: 6%, HIV-T8: 8%) (Supplementary Fig. 23c). This corresponds to a change in HIV-T1:HIV-T8 cleavage preference of >12-fold. Importantly, we did not detect any DNA cleavage using either crRNA at two predicted genomic off-target sites (Supplementary Fig. 23d, e). These data demonstrate that crRNAs containing inosine modifications can be used in combination with Cas12a to cleave polymorphic sequences in cells, albeit with reduced activity.

## Discussion

Cas9 tolerates a number of chemical alterations within the guide segment of its crRNA, including sugar modifications such as 2′O methylation^41^, 2′O-4′C linkages^11^, and 2′ deoxyribose modifications^40^, as well as phosphate backbone modifications such as phosphorothioate^41^ and phosphonoacetate^42^. In fact, 2′ deoxyribose modification of the crRNA was reported to be tolerated in all locations of the spacer segment except position 16^40^. This flexibility is quite remarkable given that the enzyme forms four direct amino acid contacts with sugar moieties within the spacer sequence, and over ten interactions with the phosphate backbone in this region^56^. Crystal structures have elucidated only one major Cas9 amino acid (Tyr1013)-base interaction, which occurs at position 1 in the crRNA targeting region^56^. Our data demonstrate that several classes of chemically unrelated non-canonical bases may be tolerated within Cas9 targeting sequences, even in positions where direct contact between Cas9 and the base or sugar backbone of the crRNA are being made^56^. For example, crRNAs containing modified bases at position 1 (a Cas9-base interaction)^56^ (Supplementary Figs. 16–19), and position 19 (a Cas9-sugar interaction)^56^ (Figs. 1, 2), were still able to direct Cas9 cleavage of target DNA, albeit with reduced activity compared the unmodified RNA in some cases.

We show that incorporation of multiple types of universal bases into Cas9 crRNAs abolishes specificity at the site of incorporation but otherwise preserves specificity (Fig. 2, Supplementary Figs. 13 and 17). These findings are in agreement with results from PCR-based studies in which incorporation of inosine or synthetic universal bases into detection or amplification primers confers partial degeneracy^35,57^. Similarly, our data indicating that the presence of universal bases in crRNAs may lower in vitro Cas9 activity via reduced DNA binding (Fig. 1, Supplementary Figs. 4–7), are reminiscent of the decreases in PCR efficiency observed using inosine-containing primers^58^. This decreased efficiency results from a decrease in duplex thermodynamic stability resulting from A-U to I-U and G-U to I-C transitions^58^. Also in agreement with our findings, the magnitude of this effect appears to be context dependent and is influenced by the nearest-neighbor 5′ and 3′ bases, following a decreasing stability trend of G-C > C-G > A-T > T-A^30^. Interestingly, we observed that Cas9 activity was lower with 2′ deoxyribose inosine substitutions compared to 2′ OMe ribose inosine substitutions (Fig. 1c, d). Since previous reports have suggested that DNA/DNA hybrids are in fact more thermodynamically stable than 2′-O-methyl RNA/DNA duplexes^34^, this could be due to a conformational/steric effect^11^. Other universal bases examined in this work such as deoxyribose 5′-nitroindole have been reported to be more destabilizing than inosine, due to the inability to form hydrogen bonds^37^. However, this effect is also context dependent, as short contiguous stretches of 5′-nitroindole are more tolerated than contiguous stretches of inosine in PCR amplification reactions^35^. Collectively, our data suggest that universal bases behave similarly in the context of crRNAs as they do in other types of nucleic acid probes. Thus, it is likely that many of the established rules governing their effective placement in an oligonucleotide are transferrable to this new application. However, future studies will be needed to test this assertion, and to determine how broadly these base modification schemes can be applied across different crRNAs.

The 1000 genomes project identified over 85 million SNPs, 3.6 million short indels, and 60,000 structural variants, underscoring the vast myriad of human genetic diversity^59^. While current CRISPR/Cas technology is not adequately equipped to deal with the challenge, this study puts forth a possible solution. We show that the incorporation of universal bases into individual crRNAs can enable simultaneous targeting of a clinically relevant polymorphic gene in vitro and in cells (Figs. 1–3, Supplementary Fig. 20). Given the extensive costs associated with personalized-medicine based clinical trials, CRISPR guide RNAs with partial degeneracy could be designed to circumvent natural genetic variation and enable all individuals in a patient population to be treated using a single, heavily validated therapeutic. Similarly, this technology could be applied in the lab to the development of guide RNAs capable of directing cleavage of a gene sequence across multiple different species for which evolutionary divergence may have occurred. However, our data suggest that certain limitations will first need to be overcome to realize this full potential. We noted in several instances that universal base-modified crRNAs yielded reduced or absent Cas9 activity in cells, despite showing strong activity in vitro (Supplementary Fig. 20). Interestingly, we found that these same crRNAs induced a delay in Cas9 cleavage kinetics in vitro, and that there was a direct correlation between slower cleavage kinetics and lower cellular modification rates (Supplementary Fig. 21). These findings are not unprecedented^11^. Previous work has shown that LNA and BNA substitutions in crRNAs also induce a delay in Cas9 enzyme kinetics that is associated with lower cleavage activity in cells^11^. It has been proposed that delayed kinetics could increase the probability that Cas9 is ejected from DNA by cellular factors prior to cutting, thereby reducing modification rates^11^. Prospective studies could address this issue by identifying ways to increase the residence time of Cas9 on DNA in cells, or by generating enzymes with kinetic properties more tailored to this application through protein evolution or engineering.

Several viruses have mutation rates that are up to 1 million times higher than their hosts^60^. This statistic highlights the obstacle that genetic variation presents for detecting and targeting pathogens using CRISPR systems. One study aiming to treat HIV-1 infection using CRISPR/Cas9 documented effective viral escape through evolved mutations^54^. Here we show that universal bases can be incorporated into Cas12a guide RNAs to enable detection of evolved viral gene sequences using the DETECTR platform (Fig. 4). Unlike Cas9, where rI-modified crRNAs resulted in lower in vitro activity in certain cases (Fig. 1), we found that Cas12a activity remained robust when coupled with universal base-modified crRNAs (Fig. 4), making this an ideal application of the technology. We speculate that this could be due to the fact that our Cas12a experiments measured combined *cis* and *trans* enzyme activity. It is conceivable that small to moderate decreases in *cis* cleavage activity could be fully masked by Cas12a collateral activity. Overall, we envision that this technology could be used to help reduce the false negative detection rate of the DETECTR system by imparting the platform with the flexibility to take into account pathogen evolution, either documented or predicted.

We demonstrate the potential to use universal base-modified crRNAs with Cas12a to target polymorphic viral sequences in cells (Supplementary Fig. 23). While the rI-modified crRNA we used resulted in the expected degenerate sequence cutting in cells, it did show reduced activity (Supplementary Fig. 23). This could be due to the apparent lack of Cas12a *trans* activity in cells^61^, or also the result of slower enzyme kinetics, as described above for Cas9.

In addition to targeting known polymorphic sequences, this technology could be used to target pathogens for which only incomplete sequence information is known. For example, future studies could assess if contiguous stretches of universal bases can be incorporated into crRNAs to reduce the requisite length of the spacer sequence. Theoretically, this would enable targeting of shorter sequences in emergent pathogens for which all 20 bp of the sequence may be unavailable.

This work details the first demonstration that incorporation of non-canonical universal bases into Cas9/Cas12a guide RNAs can be tolerated and impart selectively degenerate specificity. We demonstrate the applicability of this technology to targeting a series of polymorphic gene variants in vitro and in cells using a single guide RNA. Furthermore, we delineate how this technology can be applied to diagnostics to circumvent false-negative results caused by pathogen evolution. By relaxing the current restrictions of guide RNA targeting, we anticipate that this study will expand the operative capabilities of Cas9, Cas12a/Cpf1, and potentially other CRISPR systems.

## Methods

### Chemical reagents and design and synthesis of crRNAs

Unless otherwise noted, all chemical reagents were purchased from Sigma-Aldrich. DNA oligonucleotides and tracrRNA were purchased from Integrated DNA Technologies (IDT). crRNAs were rationally designed based on clinical polymorphism data for the *HLA-B*^62^, *ABO*^62^, and HIV^53^ gene sets. *HLA-C* crRNAs were designed as described in the manuscript. Two of the four SNPs chosen for the *ABO* target site are found in the most common *ABO* alleles and are linked to changes in blood type^43^. The polymorphisms seen in the HIV gene set are linked to the formation of drug-resistant mutations in a domain of the viral protease^52^. Cas9 crRNAs were designed based on the presence of a 3′-NGG PAM directly adjacent to a 20 bp target site for the *HLA-B*, *HLA-C* and *ABO* genes. Cas12a crRNAs were designed based on a 5′-TTTN sequence directly adjacent to the 23 bp target site for the HIV protease gene. Sequences for these crRNAs can be found in Supplementary Table 1. Chemical synthesis of the crRNAs was performed by Bio-Synthesis Inc. and GeneLink Inc.

### Preparation of DNA targets

Forward and reverse ssDNA target inserts were designed for Cas9 target sites. The oligos used to make the DNA targets are listed in Supplementary Table 2. The forward and reverse ssDNA sequences were annealed by heating to 95 °C for 5 mins, then cooling to 25 °C over 1 h. Next, pUC19 plasmid (Invitrogen) and the annealed dsDNA target inserts were double-digested with *HindIII* and *XbaI* (NEB). These were ligated and then transformed into DH5α *E. coli*. Proper insertion was confirmed by performing Sanger sequencing. DNA targets for in vitro experiments concerning *HLA-C* were prepared through PCR amplification of genomic DNA (gDNA) from 293 T or HeLa cells using primers listed in Supplementary Table 2.

### Expression and purification of S. pyogenes Cas9

Recombinant Cas9 and dCas9 were purified as previously described^45^. Briefly, *E. coli* Rosetta (DE3) cells were transformed with a plasmid encoding either *S. pyogenes Cas9* or catalytically-dead *S. pyogenes Cas9* (dCas9) fused to an N-terminal 6xHis-tag, MBP, and TEV site (Addgene #39312 and #39318, respectively). 25 mL of LB broth containing 25 µg mL^−1^ of kanamycin was inoculated and grown overnight (~16 hrs) at 37 °C. These cells were diluted 1:100 in the same growth media and grown at 37 °C until an OD_600_ of 0.8 before moving to 18 °C for 30 mins. Protein production was induced by the addition of isopropyl-ß-D-1-thiogalactopyranoside (IPTG) to a final concentration of 0.5 mM. After induction for 16 h, the cells were harvested by centrifugation for 15 min at 2700 × *g* and resuspended in 15 ml/L culture lysis buffer (20 mM Tris-Cl, pH 8.0, 250 mM NaCl, 5 mM imidazole, pH 8.0) supplemented with lysozyme and 0.1 M PMSF. This was incubated on ice for 30 mins before being further lysed by sonication (30 sec pulse-on and 60 secs pulse-off for 7.5 min at 60% amplitude) and centrifuged at 30,000 × *g* for 1 h to obtain cleared lysate. The lysate was applied to a 1 mL HisTrap FF Crude column (GE Healthcare) attached to an AKTA Start System (GE Healthcare), washed (20 mM Tris-Cl, pH 8.0, 250 mM NaCl, 10 mM imidazole, pH 8.0), and eluted with a single concentration of imidazole (20 mM Tris-Cl, pH 8.0, 250 mM NaCl, 250 mM imidazole, pH 8.0). Fractions containing Cas9 were pooled, TEV protease was added, and this was dialyzed into ion-exchange buffer overnight (20 mM HEPES-KOH, pH 7.5, 150 mM KCl, 10% (v/v) glycerol, 1 mM dithiothreitol (DTT), 1 mM EDTA). After dialysis, the sample was centrifuged to remove cleaved MBP. The supernatant was loaded onto a 1 mL HiTrap SP FF column (GE Healthcare), washed (20 mM HEPES-KOH, pH 7.5, 100 mM NaCl), and eluted with a 0–50% gradient of NaCl (20 mM HEPES-KOH, pH 7.5, 1 M NaCl). Fractions containing purified Cas9 were concentrated using a 50 kDa centrifugal filter (Pall). During concentration, the buffer was exchanged into storage buffer (20 mM HEPES-KOH, pH 7.5, 500 mM NaCl, 1 mM DTT). Concentrated protein was aliquoted and stored at −80 °C.

### In vitro cleavage assays (Cas9)

In vitro DNA cleavage assays were performed as previously described^11^. Briefly, plasmid templates containing DNA targets were amplified with pUC19F/R primers listed in Supplementary Table 2. gRNAs were created by mixing equimolar amounts of tracrRNA (IDT) and crRNA (GeneLink) in Nuclease Free Duplex Buffer (IDT), and then heating to 95 °C for 5 min before cooling to 25 °C over 1 h. Sequences for crRNAs and tracrRNA are listed in Supplementary Table 1. Each reaction consisted of the amplified 5 nM DNA target with 40 nM Cas9 protein and 80 nM gRNA, unless otherwise stated. Initially, Cas9 and gRNA were incubated in 1× NEB 3.1 buffer (100 mM NaCl, 50 mM Tris-HCl, 10 mM MgCl_2_, 100 µg/ml BSA, pH 7.9) at 25 °C for 10 mins. Subsequently, the DNA template was added and the reaction was incubated at 37 °C for 3 h (or in the time indicated in the figure legend for kinetic experiments). Reactions were stopped by purifying the DNA with a MinElute PCR Purification Kit (Qiagen). Cleavage products were run on a 1% agarose gel and imaged with an Amersham Imager 600 (GE Healthcare). Densitometry was performed using Image J.

### Library construction for high-throughput specificity profiling

Pre-selection libraries were generated as previously described^11^. Briefly, 10 pmol of each partially randomized oligo (IDT, sequences are listed in Supplementary Table 1) was circularized with CircLigase II ssDNA Ligase Kit (Epicenter). 5 pmol of the circularized ssDNA was used as a template for the Illustra TempliPhi Amplification Kit (GE Healthcare) according to the manufacturer’s protocol. The resulting amplified libraries were quantified with a Qubit 2.0 Fluorometer (Invitrogen).

### In vitro high-throughput specificity profiling

A specificity profile of the modified crRNAs was created as previously described^11^. Briefly, 200 nM of the pre-selection library was incubated with 1000 nM gRNA and 1000 nM Cas9 in NEB Buffer 3.1 for 1 h at 37 °C to create the post-selection library. In addition, 200 nM of the library was incubated with 2U of BspMI using the same reaction conditions as above, to create the final pre-selection library. Both digestion reactions were purified using a QiaQuick PCR Purification Kit (Qiagen) and ligated to 10 pmol of barcoded adaptor S50X-F/R (post-selection) or lib_adapter1 with ABO/HLA_lib_adapter2 (pre-selection) using 1000U of T4 DNA Ligase (NEB) for 16 hrs at room temperature. Ligation reactions were purified using the MinElute PCR Purification Kit (Qiagen) then amplified using primer PE2_short with barcoded primer HLA/ABO-N70X (post-selection) or primer lib_PCR_F with barcoded ABO/HLA_PCR_R (pre-selection) using Q5 Hot Start High-Fidelity Master Mix (NEB). Products were gel extracted and purified using MinElute Gel Extraction Kit (Qiagen) and quantified with a Qubit 2.0 Fluorometer. Finished libraries were run on a HiSeq 2000 (Novogene), demultiplexed, and analyzed as previously described^45^. The sequences used for this protocol are listed in Supplementary Table 2.

### Electrophoretic mobility shift assay (EMSA)

EMSA assays were performed as previously described^11^, with minor modifications. Briefly, the dsDNA target was created by annealing the ssDNA target and Cy-5 labelled non-target strands in a 1.5:1 ratio. dsDNA substrate, gRNA, and dCas9 were diluted in the binding buffer to working concentrations (20 mM HEPES, pH 7.5, 50 mM KCl, 2 mM MgCl_2_, 0.01% Triton X-100, 0.1 mg mL^−1^ bovine serum albumin, 10% glycerol) and 50 µg/mL of heparin was added. 500 nM dCas9 and 750 nM gRNA were incubated at room temperature for 10 mins. For titration reactions, 0, 10, 25, 50, 100, 250, 500 nM & 1 µM of dCas9 and 1.5× gRNA was used. 10 nM Cy-5 labelled annealed DNA substrate was then added and incubated for 1 h at 37 °C. The reaction was run on a 10% TBE-2mM MgCl_2_ polyacrylamide gel at 4 °C and imaged with an Amersham Imager 600 (GE Healthcare). Densitometry was used to measure percent binding (Image J).

### Determination of crRNA–DNA heteroduplex melting temperature

Equimolar amounts of crRNA and complementary ssDNA were combined in Duplex Buffer (30 mM HEPES, pH 7.5, 100 mM Potassium Acetate) (IDT) to a final concentration of 2 µM. 100× SYBR Green I was then added to yield a final concentration of 10×. The solution was added to a CFX96 Real-Time System (BioRad). The following program was run to anneal the RNA/DNA heteroduplex: 5 min at 95 °C followed by cooling to 25 °C at 0.1 °C s^−1^. To measure the melting temperature, the heteroduplex was heated to 45 °C and then subsequently heated at a rate of 0.1 °C s^−1^ to 95 °C. The SYBR Green I fluorescent signal was used to generate a melt curve from which a T_M_ value was determined.

### Cell-based RFP/GFP reporter assay

Target sites were cloned into a pRGS backbone (PNA Bio Inc.) containing an RFP reporter and two out-of-frame GFP reporters, as previously described^63^. gRNA was annealed as described above. HeLa-Cas9 cells (previously authenticated and shown to be free of mycoplasma)^11^ were cultured in high-glucose DMEM media with pyruvate (Gibco) supplemented with 10% FBS/1× pen-strep/1× glutamine (Gibco) and 5 µg mL^−1^ Blasticidin S HCl (Gibco) at 37 °C in 5% CO_2_. Transfection of the HeLa-Cas9 cells was performed using DharmaFECT Duo (Dharmacon), according to manufacturer instructions for the CRISPR system. The degree of target sequence cleavage was calculated based on the %GFP + /%RFP + cells using an Attune NxT Flow Cytometer (Invitrogen).

### Expression and purification of humanized Lachnospiraceae bacterium Cas12a/Cpf1

Humanized Cpf1 was purified as previously described^64^. Briefly, *E. coli* Rosetta (DE3) pLyseS (EMD Millipore) cells were transformed with a plasmid encoding humanized *Lachnospiraceae bacterium* Cpf1 fused to an N-terminal 6xHis-tag, MBP, TEV site, and C-terminal NLS and HA tag (Addgene # 90096). 25 mL of Terrific broth containing 100 µg mL^−1^ of carbenicillin was inoculated and grown overnight (~16 hrs) at 37 °C. These cells were diluted 1:100 in the same growth media and grown at 37 °C until OD_600_ of 0.2. This was moved to 21 °C and grown until an OD_600_ of 0.6 before induction with IPTG to a final concentration of 0.5 mM for 14–18 h. After induction, the cells were harvested by centrifugation for 15 min at 2700 × *g* and resuspended in 50 mL/L culture of lysis buffer (50 mM HEPES pH 7, 2 M NaCl, 5 mM MgCl2, 20 mM imidazole, pH 8.0), supplemented with lysozyme and 0.1 M PMSF. Cell lysis and protein purification were performed as described above. lbCpf1 was stored in Cpf1 storage buffer (50 mM Tris-HCl pH7.5, 2 mM DTT, 5% glycerol, 500 mM NaCl).

### In vitro cleavage assays (Cas12a)

In vitro DNA cleavage reactions for Cas12a were performed as described above with slight modifications. Each reaction consisted of amplified 10 nM DNA target with 100 nM Cas12a protein and 125 nM gRNA. Reactions were incubated at 37 °C for 30 mins (or the time indicated in the figure legends for kinetic experiments). For experiments involving titrations of Cas12a RNP, each reaction consisted of amplified 10 nM DNA target with 0 nM, 10 nM, 25 nM, 50 nM, 100 nM, 150 nM, 250 nM, 500 nM, or 1 µM of Cas12a protein and 1.25× gRNA. Reactions were incubated at 37 °C for 30 mins. Sequences for the crRNAs used in these experiments are listed in Supplementary Table 1.

### DETECTR assay

DETECTR assays were performed as previously described^13^, with minor modifications. Briefly, target constructs were created with a pUC19 backbone as described above. Recombinase Polymerase Amplification (RPA) reactions were performed using the target plasmid constructs as the template and pUC19 RPA F/R primers. This reaction was incubated at 37 °C for 10 mins. 250 nM LbCas12a, 312.5 nM crRNA, and 250 nM ssDNA-FQ reporter were incubated at 25 °C for 10 mins and added directly to the reaction. Subsequently, reactions were incubated at 37 °C in a fluorescent plate reader (Spectramax i3, Molecular Devices) for 2 hrs with measurements taken every 2 mins (λ_ex_: 535 nm; λ_em_: 595 nm). The sequences used for this experiment are listed in Supplementary Table 2.

### Generation of Flp-In 293-Cas12a-HIV-T1/T8 cells

Parental Flp-In 293 cells were obtained from ThermoFisher (catalog number R75007) and were cultured in high-glucose DMEM media containing pyruvate (Gibco), supplemented with 10% FBS, 1X Penicillin-Streptomycin (Gibco) and 100 µg/mL Zeocin (Invitrogen). For Flp-In 293-Cas12a-HIV-T1/T8 cells, Zeocin was substituted with 100 µg/mL Hygromycin B (Invitrogen) and 300 µg/mL Geneticin (Gibco). All cells were cultured in a 5% CO_2_ atmosphere. Stable integration of HIV-T1 and -T8 Cas12a target sites into parental Flp-In 293 cells was performed as described by the manufacturer. Briefly, oligonucleotides containing the Cas12a target site of interest (listed in Supplementary Table 2) were annealed and ligated into BamHI / XhoI double-digested pcDNA5 plasmid containing an FRT site (Addgene #127108). Sequence-verified pcDNA5-HIV-T1/T8 plasmids were then co-transfected with pCSFLPe (Addgene #31130) at a ratio of 1:9 (w/w) into Flp-In 293 cells using Effectene Transfection Reagent (Qiagen) according to the manufacturer’s instructions. 24 hrs after transfection, media was replaced with fresh DMEM lacking Zeocin. The next day cells were split at a confluence of 30% into media containing 100 µg/mL Hygromycin B. Following 2 weeks of selection, cells were singly sorted on a BD FACS Aria III instrument by the Flow Cytometry Core at the University of Alberta into the wells of a 96-well plate for monoclonal expansion. Viral particles for Cas12a expression were generated in 293 T cells transfected with plenti-Lb-Cas12a-2xNLS (Addgene #155046), psPAX2 (Addgene #12260) and pMD2.G (Addgene #12259) using Effectene Transfection Reagent (Qiagen) according to the manufacturer’s instructions. Supernatant containing the viral particles was harvested 48 hrs after transfection and filter sterilized before being used to transduce Flp-In 293-HIV-T1 and Flp-In 293-HIV-T8 cells. 48 h after infection, media was replaced with DMEM containing 300 µg/mL Geneticin.

### Lipid transfection of crRNAs into stable cell lines

Cells stably expressing Cas9 or Cas12a were transfected with crRNAs to a final concentration of 60 nM using Lipofectamine RNAiMAX (Invitrogen) according to manufacturer’s instructions.

### Cellular cleavage assays

72 hrs after transfection, gDNA from transfected cells was isolated using the DNeasy Kit (Qiagen) and quantified using a NanoPhotometer NP80 (Implen) spectrophotometer. Target-specific primers (listed in Supplementary Table 2) were used to PCR amplify the desired site, with 100 ng of gDNA used as template. PCR products were purified using QIAquick PCR Purification Kit (Qiagen). 200 ng of product was subject to T7 endonuclease I (T7E1) digestion as described by the manufacturer (NEB). Cleavage assays were resolved on a 2% TAE agarose gel.

### Calculations, statistics & reproducibility

Indel percentages were calculated using the formula indel (%) = 100 × (1 − (1 − fraction_cut_)^0.5^). Replicate numbers and measures of variance are included in the Figure legends. Experiments were not randomized, but the high-throughput specificity profiling assays employed large libraries of partially randomized sequences. No statistical method was used to predetermine sample size, and no data were excluded from the analyses. The investigators were not blinded to allocation during experiments and outcome assessment.

### Reporting summary

Further information on research design is available in the Nature Research Reporting Summary linked to this article.

## Supplementary information

Supplementary Information

Reporting Summary

## Data Availability

Data associated with this paper are included in this published article and its associated Supplementary Information files. All high-throughput sequencing data files associated with this paper have been deposited in the NCBI SRA database and are available under accession number: PRJNA669024. Databases that were used in the selection of cellular target sequences are publicly accessible: Ensembl (https://uswest.ensembl.org/index.html) and HEK293 Genome (http://www.hek293genome.org/v1/index.php). Source data are provided with this paper.

## Source data

Source Data

## Code availability

Python scripts used for data analysis are available upon request.
