# Supplementary material for: Guide RNAs containing universal bases enable Cas9/Cas12a recognition of polymorphic sequences
Source: Nat Commun. 2022 Mar 25;13:1617. doi: 10.1038/s41467-022-29202-x (PMC8956631; doi:10.1038/s41467-022-29202-x)
Supplement: Supplementary file 1 — Supplementary Information [file 41467_2022_29202_MOESM1_ESM.pdf]

Guide RNAs containing universal bases enable Cas9/Cas12a recognition of polymorphic sequences

Amanda R. Krysler, Christopher R. Cromwell, Tommy Tu, Juan Jovel, Basil P. Hubbard

Supplementary Information

**Supplementary Figure 1**	**Applications of universal bases to CRISPR/Cas9 and CRISPR/Cas12a systems.**
**Supplementary Figure 2**	***In vitro* cleavage of *ABO* variant sequences by Cas9 using ABO-RNA.**
**Supplementary Figure 3**	**Cleavage of polymorphic sequences by Cas9 using crRNAs containing universal bases.**
**Supplementary Figure 4**	***In vitro* activity of inosine-modified crRNAs on ABO-T1 target.**
**Supplementary Figure 5**	**Effect of inosine modifications on the kinetics and activity of Cas9.**
**Supplementary Figure 6**	**Effect of inosine modifications in crRNAs on the ability of dCas9 to bind target DNA.**
**Supplementary Figure 7**	**Relationship between cleavage activity and target engagement of Cas9 using inosine-modified crRNAs.**
**Supplementary Figure 8**	**Effect of ribose inosine modifications on crRNA-DNA target heteroduplex melting temperature.**
**Supplementary Figure 9**	**Distribution of mutations in pre- and post-selection libraries for ABO-RNA and inosine-modified crRNAs.**
**Supplementary Figure 10**	**Change in specificity score of inosine-modified crRNAs compared to ABO-RNA.**
**Supplementary Figure 11**	**Average specificity score of ribose inosine modified positions in the *ABO* crRNAs.**
**Supplementary Figure 12**	**Distribution of mutations in pre- and post-selection libraries for ABO-RNA and universal base-modified crRNAs.**
**Supplementary Figure 13**	***In vitro* specificity profiles for *ABO* crRNAs containing various universal base modifications.**
**Supplementary Figure 14**	**Change in specificity score of universal base-modified crRNAs compared to ABO-RNA.**
**Supplementary Figure 15**	**Average specificity score of universal base modified positions in the *ABO* crRNAs.**
**Supplementary Figure 16**	**Distribution of mutations in pre- and post-selection libraries for HLA-RNA and inosine-modified crRNAs.**
**Supplementary Figure 17**	***In vitro* specificity profiles for *HLA* crRNAs containing inosine base modifications.**

**Supplementary Figure 18**	**Change in specificity score of inosine-modified crRNAs compared to HLA-RNA.**
**Supplementary Figure 19**	**Average specificity score of ribose inosine modified positions in the *HLA* crRNAs.**
**Supplementary Figure 20**	**Cas9 cleavage activity using HLA-C-RNA and universal base-modified crRNAs *in vitro* and on the endogenous *HLA-C* locus in 293T and HeLa cells.**
**Supplementary Figure 21**	***In vitro* Cas9 cleavage kinetics using HLA-C-RNA or universal base-modified crRNAs.**
**Supplementary Figure 22**	**Titration of Cas12a RNP containing HIV-RNA or HIV-rI-1 against select target sequences *in vitro.***
**Supplementary Figure 23**	**Cleavage of HIV target sequences by Cas12a with unmodified and inosine modified crRNAs in cells.**
**Supplementary Table 1**	**Sequences of crRNAs and tracrRNA.**
**Supplementary Table 2**	**Sequences of oligonucleotides.**

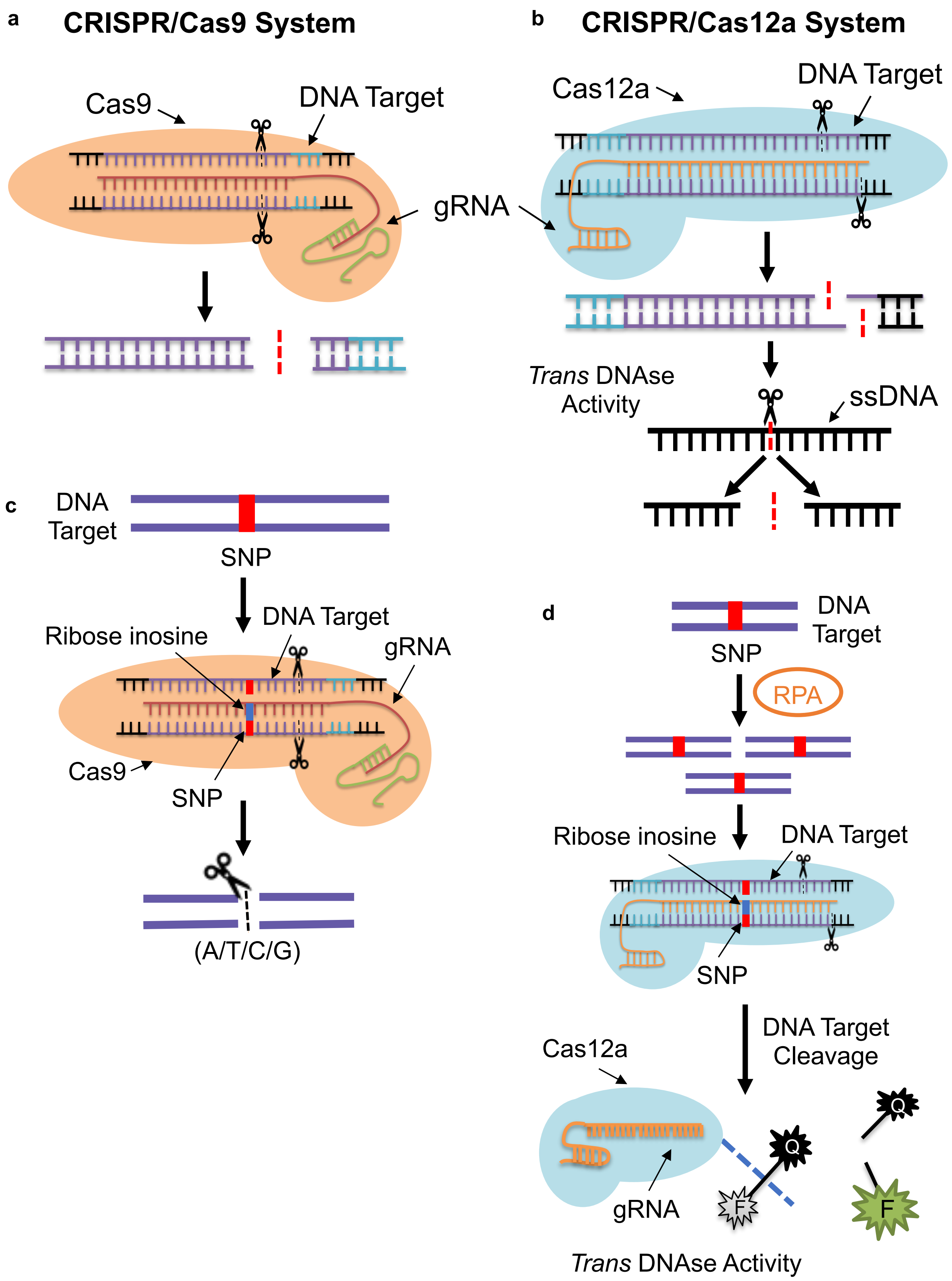
a
CRISPR/Cas9 System
Cas9
DNA Target
gRNA
b
CRISPR/Cas12a System
Cas12a
DNA Target
Trans DNAse Activity
ssDNA
c
DNA Target
SNP
Ribose inosine
DNA Target
gRNA
Cas9
SNP
(A/T/C/G)
d
DNA Target
SNP
RPA
Ribose inosine
DNA Target
SNP
DNA Target Cleavage
Cas12a
Q
F
gRNA
Q
F
Trans DNAse Activity

Supplementary Figure 1. Applications of universal bases to CRISPR/Cas9 and CRISPR/Cas12a systems. a Diagram outlining the CRISPR/Cas9 DNA cleavage mechanism. After forming a ribonucleoprotein complex with a designed gRNA, Cas9 identifies its DNA target sequence based on the presence of a PAM sequence (blue section of the DNA target) and base-pair interactions between the DNA and the 20-nt spacer sequence of the gRNA. If successful pairing occurs, Cas9 induces a blunt, double-strand cut in the DNA. **b** Diagram outlining the CRISPR/Cas12a DNA cleavage mechanism. After forming a ribonucleoprotein complex with a designed gRNA, Cas12 identifies its DNA target sequence based on the presence of a PAM sequence (blue section of the DNA target) and base-pair interactions between the DNA and the 23-25-nt spacer sequence of the gRNA. If a highly matched sequence is found, Cas12a induces a staggered double-strand DNA cut in the target (*cis* cleavage). In addition, this process activates the collateral, or *trans* DNase activity of Cas12a which acts to subsequently degrade nearby ssDNA in a non-specific manner. Diagrams depicting applications where universal bases substituted into the gRNA could **c** enable the simultaneous targeting/cleavage of multiple DNA sequence variants in cells or **d** allow for the detection of polymorphic sequences. 'SNP' denotes single nucleotide polymorphism, 'RPA' denotes recombinase polymerase amplification, 'F' denotes fluorophore, and 'Q' denotes quencher.

a

Target	DNA Sequence (5′→3′)	# of SNPs	Target	DNA Sequence (5′→3′)	# of SNPs
**ABO-T1**	CATGGAGTTCCGCGACCACGTGG	0	**ABO-T9**	CATGGAGTTCTGCGACCACGTGG	1
**ABO-T2**	CATGGAGATCCGCGACCACGTGG	1	**ABO-T10**	CATGGAGATCTGCGACCACGTGG	2
**ABO-T3**	CATGGAGTTCCGCGACCATGTGG	1	**ABO-T11**	CATGGAGTTCTGCGACCATGTGG	2
**ABO-T4**	CATGGAGTTCCGCGACCACATGG	1	**ABO-T12**	CATGGAGTTCTGCGACCACATGG	2
**ABO-T5**	CATGGAGATCCGCGACCACATGG	2	**ABO-T13**	CATGGAGATCTGCGACCATGTGG	3
**ABO-T6**	CATGGAGATCCGCGACCATATGG	3	**ABO-T14**	CATGGAGATCTGCGACCACATGG	3
**ABO-T7**	CATGGAGATCCGCGACCATGTGG	2	**ABO-T15**	CATGGAGTTCTGCGACCATATGG	3
**ABO-T8**	CATGGAGTTCCGCGACCATATGG	2	**ABO-T16**	CATGGAGATCTGCGACCATATGG	4

b

ABO-RNA: 5′-CAUGGAGUUCCGCGACCACG-3′

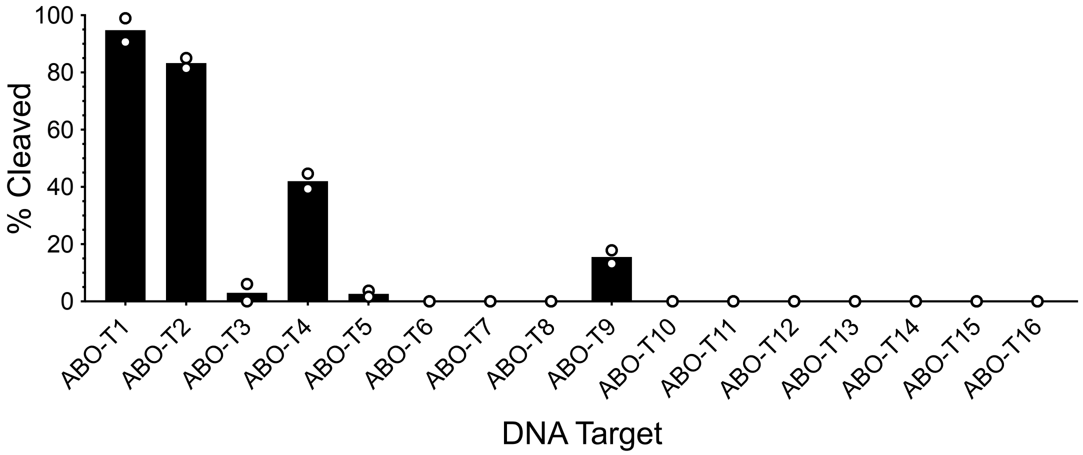

Supplementary Figure 2. *In vitro* cleavage of *ABO* variant sequences by Cas9 using ABO-RNA. a List of DNA targets corresponding to sequences in the *ABO* gene based on clinical polymorphism data. SNPs are indicated with red lettering. The PAM sequence is underlined. **b** Bar graph showing the relative amount of DNA cleavage resulting from *in vitro* reactions containing Cas9 with ABO-RNA versus the indicated DNA target sequences; Mean with individual data points shown (n = 2 independent experiments). Cleavage percentages were calculated from corresponding agarose gels using densitometry software (ImageJ).

a

Target	DNA Sequence (5′→3′)	Allele Frequency (%)
**ABO-T5**	CATGGAG**A**TCCGCGACCAC**A**TGG	0.0038
**ABO-T6**	CATGGAG**A**TCCGCGACCA**TA**TGG	0.00061

b

ABO-RNA: 5′-CAUGGAGUUCCGCGACCACG-3′

[*] = Modification

crRNA: 5′-CAUGGAG[*]UCCGCGACCAC[*]-3′

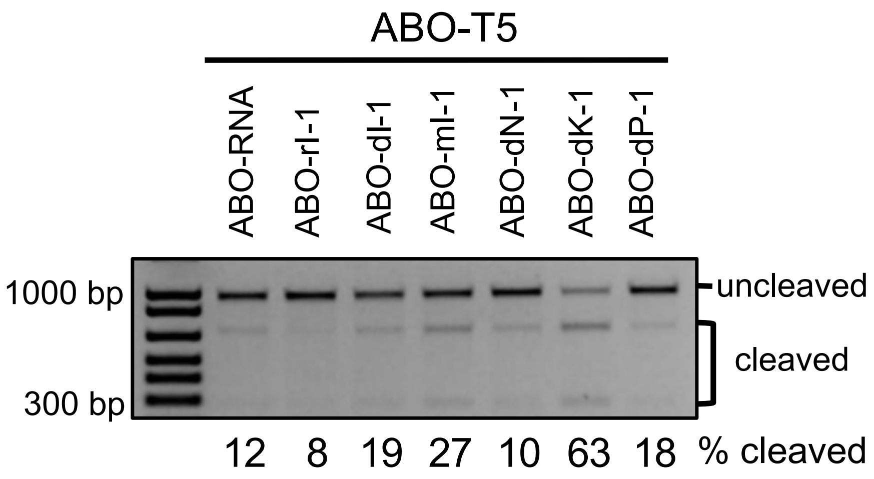

c

ABO-RNA: 5′-CAUGGAGUUCCGCGACCACG-3′

[*] = Modification

crRNA: 5′-CAUGGAG[*]UCCGCGACCA[*][*]-3′

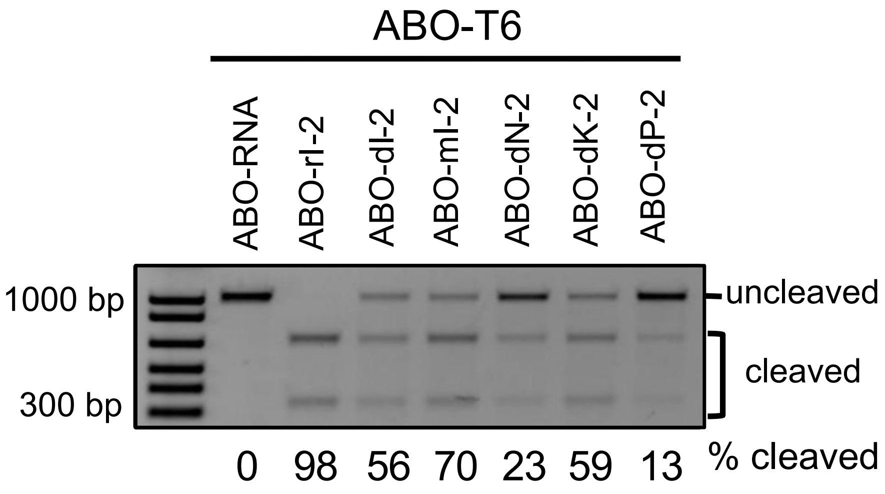

Supplementary Figure 3. Cleavage of polymorphic sequences by Cas9 using crRNAs containing universal bases. a List of DNA target sequences. SNPs are indicated with red lettering. The PAM sequence is underlined. Representative gels showing cleavage of **b** ABO-T5 or **c** ABO-T6 DNA targets by Cas9 using the indicated crRNAs. The bottom two bands in the gel represent the cleaved DNA substrate while the top band corresponds to the undigested substrate. Reactions were performed using fixed concentrations of gRNA (80 nM) and Cas9 (40 nM). Quantification of cleavage percentages was performed using ImageJ. Cleavage experiments were performed in duplicate with similar results.

a

Name	crRNA Sequence (5′→3′)
**ABO-RNA**	CAUGGAGUUCCGCGACCACG
**ABO-rI-1**	CAUGGAG[I]UCCGCGACCAC[I]
**ABO-rI-2**	CAUGGAG[I]UCCGCGACCA[I][I]
**ABO-rI-3**	CAUGGAG[I]UCCGCGACCACG
**ABO-rI-4**	CAUGGAGUUC[I]GCGACCACG
**ABO-rI-5**	CAUGGAGUUCCGCGACCA[I]G
**ABO-rI-6**	CAUGGAGUUCCGCGACCAC[I]
**ABO-rI-7**	CAUGGAG[I]UC[I]GCGACCACG

Name	crRNA Sequence (5′→3′)
**ABO-rI-8**	CAUGGAG[I]UCCGCGACCA[I]G
**ABO-rI-9**	CAUGGAGUUC[I]GCGACCA[I]G
**ABO-rI-10**	CAUGGAGUUC[I]GCGACCAC[I]
**ABO-rI-11**	CAUGGAGUUCCGCGACCA[I][I]
**ABO-rI-12**	CAUGGAG[I]UC[I]GCGACCA[I]G
**ABO-rI-13**	CAUGGAG[I]UC[I]GCGACCAC[I]
**ABO-rI-14**	CAUGGAGUUC[I]GCGACCA[I][I]
**ABO-rI-15**	CAUGGAG[I]UC[I]GCGACCA[I][I]

b

ABO-T1: 5′-CATGGAGTTCCGCGACCACG<u>TGG</u>-3′

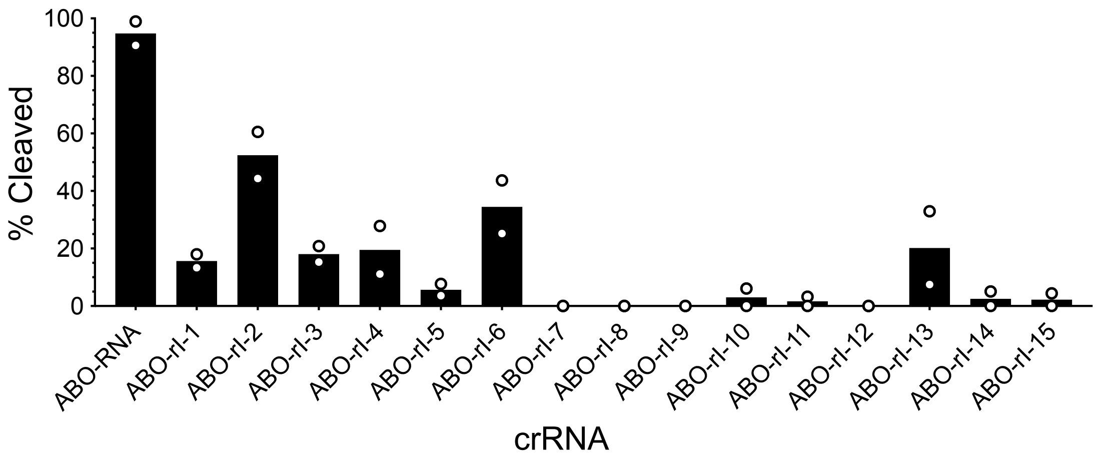

Supplementary Figure 4*. In vitro* activity of inosine-modified crRNAs on ABO-T1 target. a List of modified crRNA sequences with inosine position(s) indicated by a red [I]. **b** Bar graph showing the relative amount of DNA cleavage resulting from *in vitro* reactions using the indicated inosine-modified crRNAs versus the ABO-T1 target sequence; Mean with individual data points shown (n = 2 independent experiments). Reactions were performed using fixed concentrations of gRNA (80 nM) and Cas9 (40 nM). Quantification of cleavage percentages was performed using ImageJ.

a

Name	crRNA Sequence (5′→3′)
**ABO-RNA**	CAUGGAGUUCCGCGACCACG
**ABO-rI-1**	CAUGGAG[I]UCCGCGACCAC[I]
**ABO-rI-8**	CAUGGAG[I]UCCGCGACCA[I]G

ABO-T1 Target: 5′- CATGGAGTTCCGCGACCACG**<u>TGG</u>** -3′

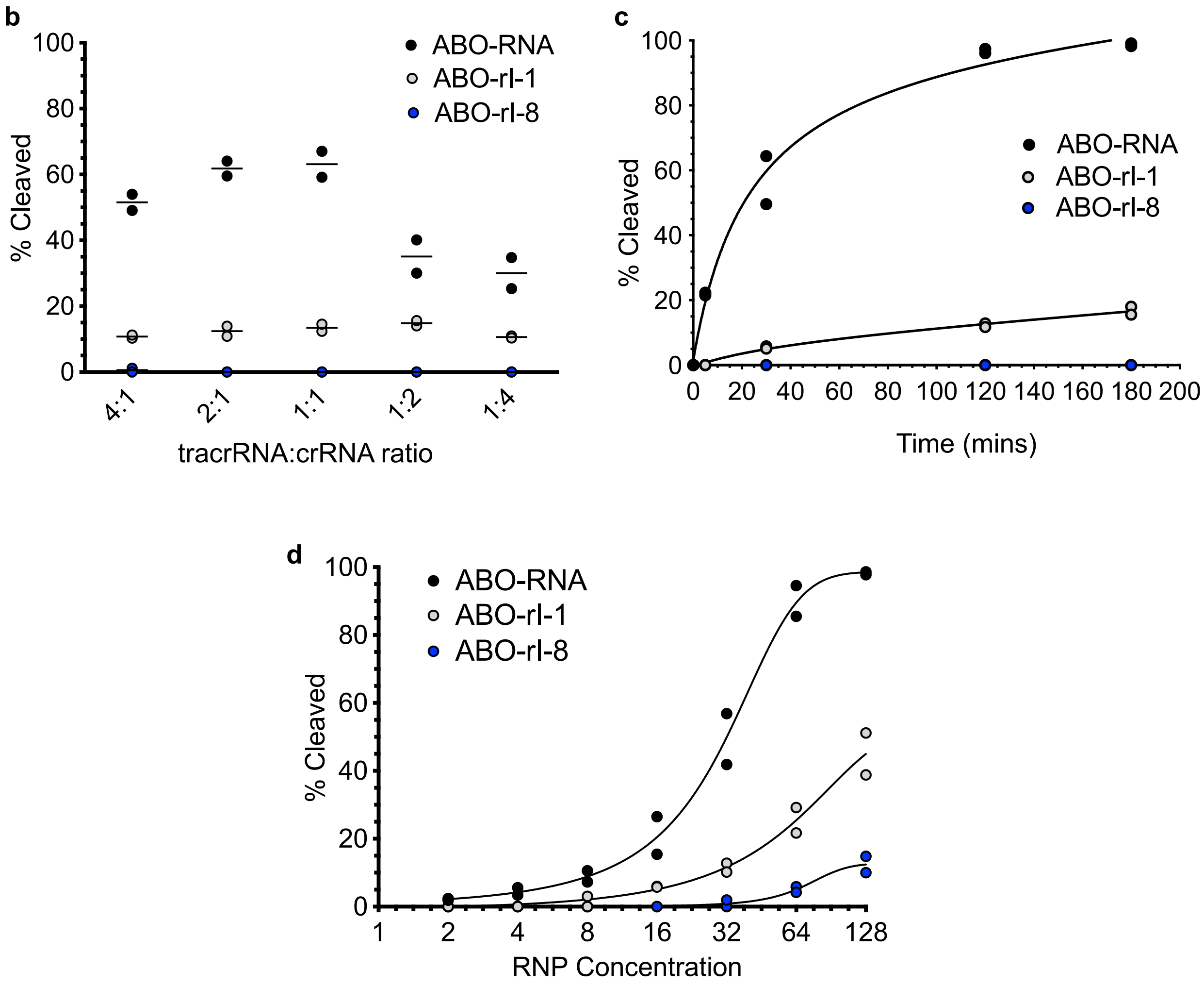

Supplementary Figure 5. Effect of inosine modifications on the kinetics and activity of Cas9. a List of inosine-modified crRNA sequences. A red [I] indicates the position of ribose inosine modifications in the crRNA sequence. **b** Graph showing Cas9 cleavage activity as a function of the tracrRNA:crRNA using either ABO-RNA, ABO-rI-1, or ABO-rI-8 versus the ABO-T1 target sequence; Mean with individual data points shown (n = 2 independent experiments). **c** Time course of Cas9 cleavage of ABO-T1 using either ABO-RNA, ABO-rI-1, or ABO-rI-8; Individual data points shown (n = 2 independent experiments). Kinetic assays were performed using fixed concentrations of gRNA (80 nM) and Cas9 (40 nM) and measured a the indicated time points. **d** Graph showing cleavage activity resulting from a titration of Cas9 RNP complex loaded with either ABO-RNA, ABO-rI-1, or ABO-rI-8 versus the ABO-T1 target sequence; Individual data points shown (n = 2 independent experiments).

a

Name	crRNA Sequence (5′→3′)
**ABO-RNA**	CAUGGAGUUCCGCGACCACG
**ABO-rI-1**	CAUGGAG[I]UCCGCGACCAC[I]
**ABO-rI-8**	CAUGGAG[I]UCCGCGACCA[I]G

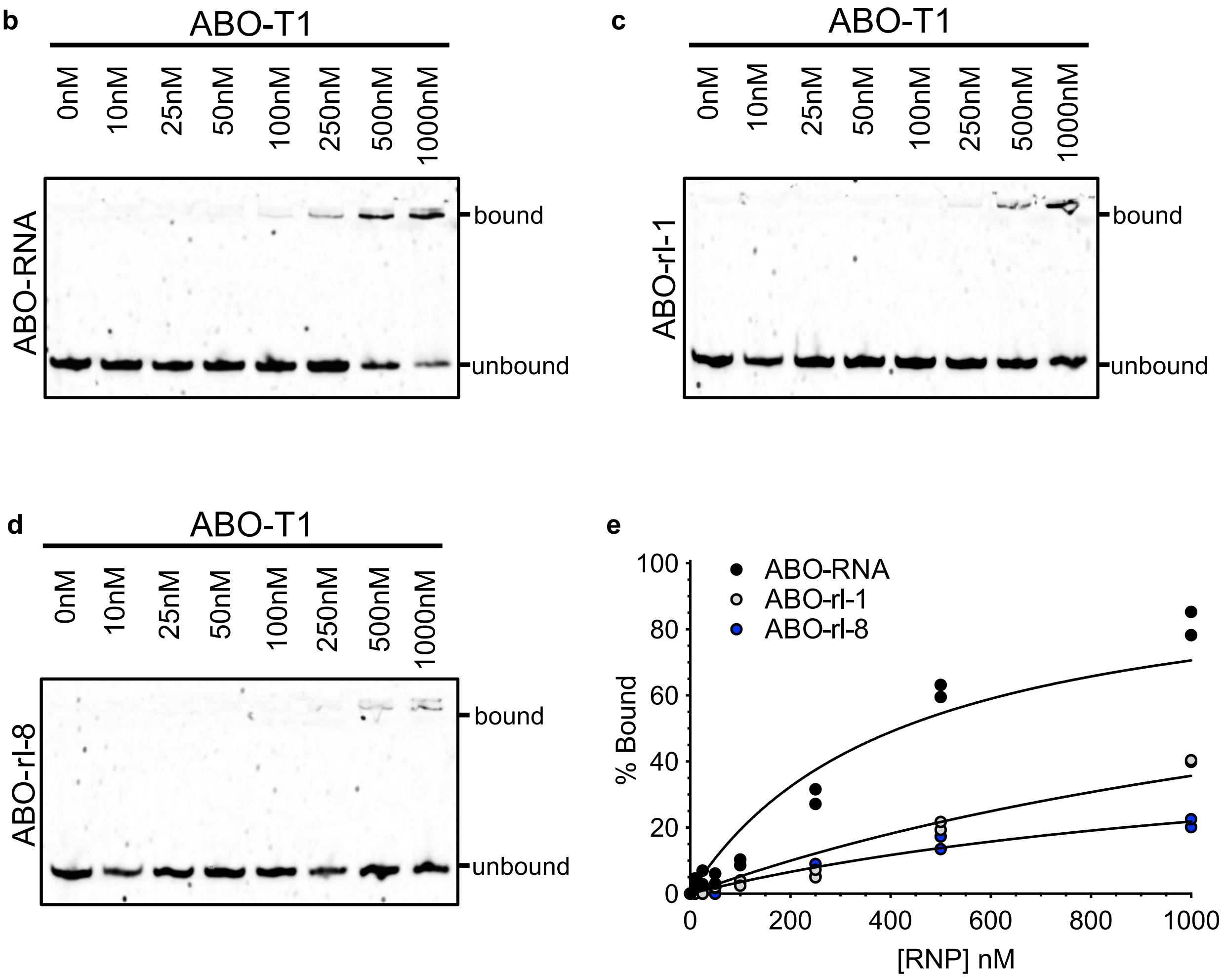

Supplementary Figure 6. Effect of inosine modifications in crRNAs on the ability of dCas9 to bind target DNA. a List of inosine-modified crRNA sequences. Red [I] indicates the position of ribose inosine modifications in the crRNA sequence. Representative gels showing binding of nuclease-deficient Cas9 (dCas9) to ABO-T1 target DNA using **b** ABO-RNA, **c** ABO-rI-1 or **d** ABO-rI-8 crRNAs. The top band represents the bound DNA substrate while the bottom band corresponds to the unbound substrate. **e** Graph showing the DNA binding resulting from a titration of Cas9 RNP complex loaded with either ABO-RNA, ABO-rI-1, or ABO-rI-8 versus the ABO-T1 target sequence; Individual data points shown (n = 2 independent experiments). Binding experiments were performed in duplicate with similar results.

a

Name	crRNA Sequence (5′→3′)	Name	DNA Sequence (5′→3′)
**ABO-RNA**	CAUGGAGUUCCGCGACCACG	**ABO-T1**	CATGGAGTTCCGCGACCACGTGG
**ABO-rI-1**	CAUGGAG[I]UCCGCGACCAC[I]	**ABO-T5**	CATGGAGATCCGCGACCACATGG
**ABO-rI-8**	CAUGGAG[I]UCCGCGACCA[I]G	**ABO-T7**	CATGGAGATCCGCGACCATGTGG

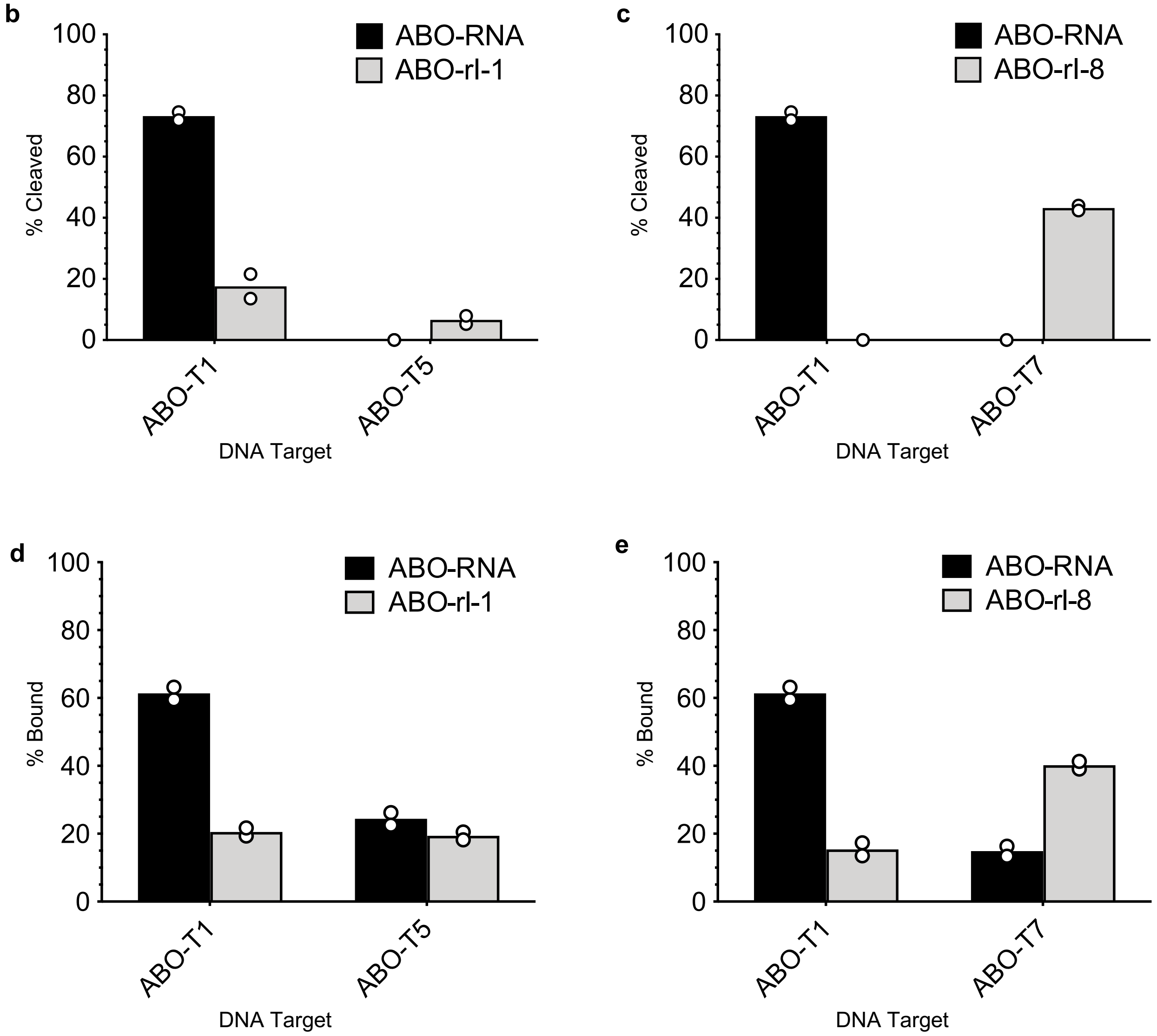

Supplementary Figure 7. Relationship between cleavage activity and target engagement of Cas9 using inosine-modified crRNAs. a List of inosine-modified crRNA sequences and DNA target sequences. Red [I] indicates the position of ribose inosine modifications in the crRNA sequence. SNPs in DNA targets are indicated with red lettering. Bar graphs showing the relative amount of DNA cleavage resulting from *in vitro* reactions containing Cas9 with ABO-RNA or **b** ABO-rI-1 or **c** ABO-rI-8 versus the indicated target sequences; Mean with individual data points shown (n = 2 independent experiments). Bar graphs showing the relative amount of DNA binding resulting from *in vitro* reactions containing dCas9 with ABO-RNA or **d** ABO-rI-1 or **e** ABO-rI-8 versus the indicated target sequences; Mean with individual data points shown (n = 2 independent experiments). *In vitro* binding assays were performed using fixed concentrations of crRNA (750 nM) and Cas9 (500 nM). Quantification of cleavage percentages was performed using ImageJ.

a

Name	crRNA Sequence (5′→3′)
**ABO-RNA**	CAUGGAGUUCCGCGACCACG
**ABO-rI-1**	CAUGGAG[I]UCCGCGACCAC[I]
**ABO-rI-8**	CAUGGAG[I]UCCGCGACCA[I]G

b **ABO-T1 Target:** 5′- CATGGAGTTCCGCGACCACG<u>**TGG**</u> -3′

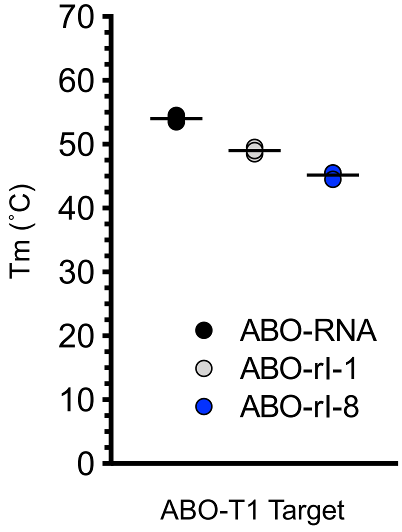

Supplementary Figure 8. Effect of ribose inosine modifications on crRNA-DNA target heteroduplex melting temperature. a List of inosine-modified crRNA sequences. Red [I] indicates the position of ribose inosine modifications in the crRNA sequence. **b** Plot showing melting temperature for heteroduplexes comprised of single-stranded sequence corresponding to ABO-T1 DNA and an RNA oligonucleotide corresponding to the spacer portion of ABO-RNA, ABO-rI-1, or ABO-rI-8; Mean with individual data points shown (n = 3 independent experiments).

a

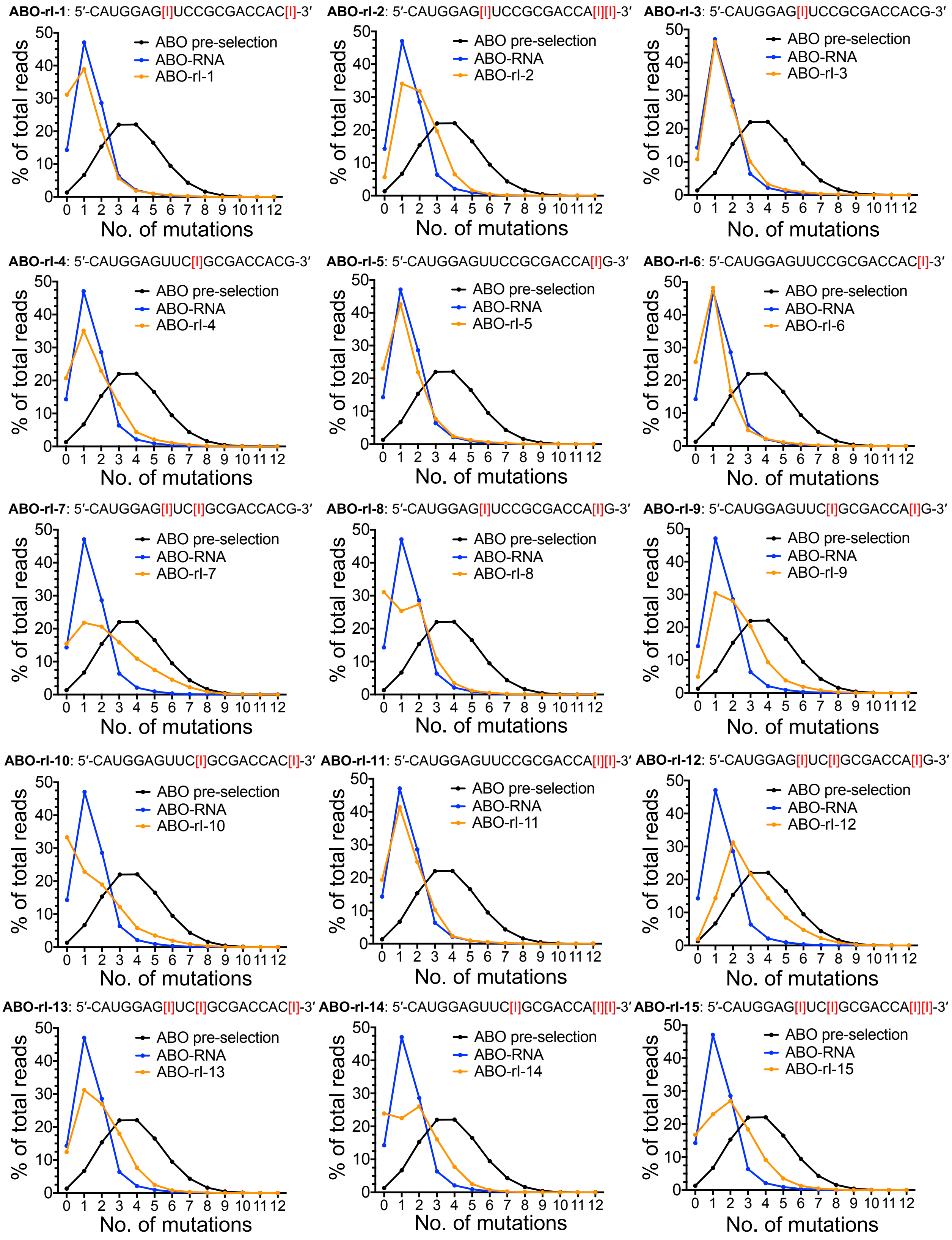
ABO-rl-1: 5′-CAUGGAG[I]UCCGCGACCAC[I]-3′
ABO-rl-2: 5′-CAUGGAG[I]UCCGCGACCA[I][I]-3′
ABO-rl-3: 5′-CAUGGAG[I]UCCGCGACCACG-3′
ABO-rl-4: 5′-CAUGGAGUUC[I]GCGACCACG-3′
ABO-rl-5: 5′-CAUGGAGUUCCGCGACCA[I]G-3′
ABO-rl-6: 5′-CAUGGAGUUCCGCGACCAC[I]-3′
ABO-rl-7: 5′-CAUGGAG[I]UC[I]GCGACCACG-3′
ABO-rl-8: 5′-CAUGGAG[I]UCCGCGACCA[I]G-3′
ABO-rl-9: 5′-CAUGGAGUUC[I]GCGACCA[I]G-3′
ABO-rl-10: 5′-CAUGGAGUUC[I]GCGACCAC[I]-3′
ABO-rl-11: 5′-CAUGGAGUUCCGCGACCA[I][I]-3′
ABO-rl-12: 5′-CAUGGAG[I]UC[I]GCGACCA[I]G-3′
ABO-rl-13: 5′-CAUGGAG[I]UC[I]GCGACCAC[I]-3′
ABO-rl-14: 5′-CAUGGAGUUC[I]GCGACCA[I][I]-3′
ABO-rl-15: 5′-CAUGGAG[I]UC[I]GCGACCA[I][I]-3′
ABO pre-selection
ABO-RNA
ABO-rl-1
ABO-rl-2
ABO-rl-3
ABO-rl-4
ABO-rl-5
ABO-rl-6
ABO-rl-7
ABO-rl-8
ABO-rl-9
ABO-rl-10
ABO-rl-11
ABO-rl-12
ABO-rl-13
ABO-rl-14
ABO-rl-15
% of total reads
No. of mutations

Supplementary Figure 9. Distribution of mutations in pre- and post-selection libraries for ABO-RNA and inosine-modified crRNAs. a Graphs indicating the number of target sequence mutations in each pre- and post-selection library. ABO pre-selection (black) and ABO-RNA post-selection (blue) data are compared to the post-selection of crRNAs containing ribose inosine(s) (orange). A red [I] denotes the position of inosine modifications in the crRNA sequence. Mutations were counted for each position throughout the 20 bp target site.

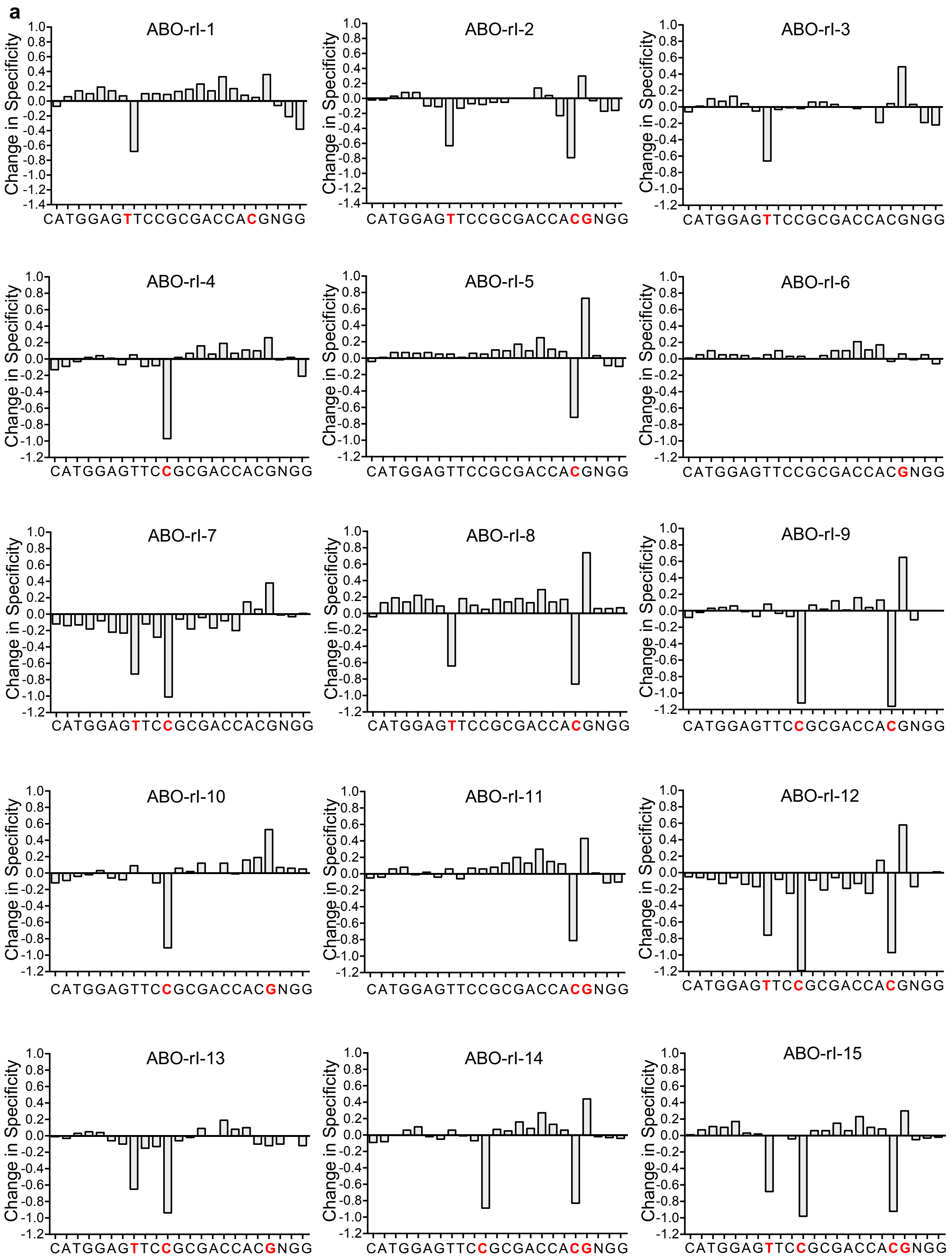
a
ABO-rl-1
ABO-rl-2
ABO-rl-3
ABO-rl-4
ABO-rl-5
ABO-rl-6
ABO-rl-7
ABO-rl-8
ABO-rl-9
ABO-rl-10
ABO-rl-11
ABO-rl-12
ABO-rl-13
ABO-rl-14
ABO-rl-15
Change in Specificity
CATGGAGTTCCGCGACCACGNGG

Supplementary Figure 10. Change in specificity score of inosine-modified crRNAs compared to ABO-RNA. a Bar graphs showing the quantitative difference in specificity score at each position in the DNA target site for inosine-modified crRNAs. SNP locations in the 20 base-pair DNA target are indicated with red lettering. The PAM is shown as “NGG” on the 3′ end of the target. A score of zero indicates no change in specificity. The difference in specificity was calculated as the specificity score(modified)−specificity score(ABO-RNA). The specificity scoring of each nucleotide position is relative to the pre-selection control library data.

a

Name	crRNA Sequence (5′→3′)	Name	crRNA Sequence (5′→3′)
**ABO-RNA**	CAUGGAGUUCCGCGACCACG	**ABO-rI-8**	CAUGGAG[I]UCCGCGACCA[I]G
**ABO-rI-1**	CAUGGAG[I]UCCGCGACCAC[I]	**ABO-rI-9**	CAUGGAGUUC[I]GCGACCA[I]G
**ABO-rI-2**	CAUGGAG[I]UCCGCGACCA[I][I]	**ABO-rI-10**	CAUGGAGUUC[I]GCGACCAC[I]
**ABO-rI-3**	CAUGGAG[I]UCCGCGACCACG	**ABO-rI-11**	CAUGGAGUUCCGCGACCA[I][I]
**ABO-rI-4**	CAUGGAGUUC[I]GCGACCACG	**ABO-rI-12**	CAUGGAG[I]UC[I]GCGACCA[I]G
**ABO-rI-5**	CAUGGAGUUCCGCGACCA[I]G	**ABO-rI-13**	CAUGGAG[I]UC[I]GCGACCAC[I]
**ABO-rI-6**	CAUGGAGUUCCGCGACCAC[I]	**ABO-rI-14**	CAUGGAGUUC[I]GCGACCA[I][I]
**ABO-rI-7**	CAUGGAG[I]UC[I]GCGACCACG	**ABO-rI-15**	CAUGGAG[I]UC[I]GCGACCA[I][I]

b

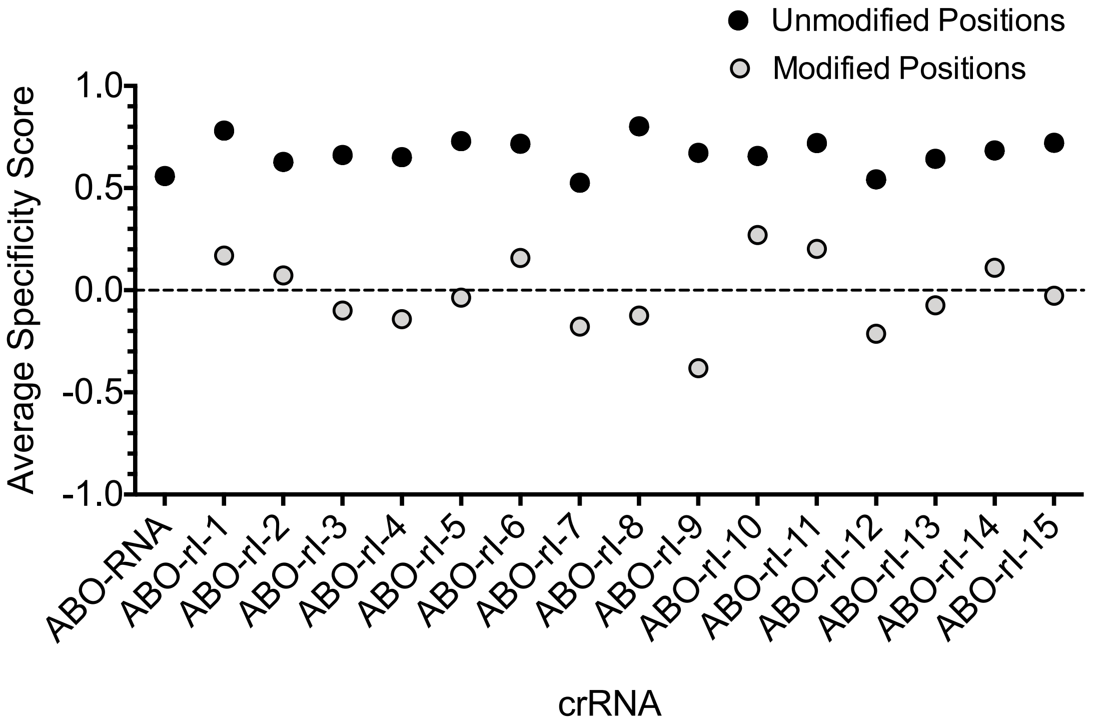

Supplementary Figure 11. Average specificity score of ribose inosine modified positions in the *ABO* crRNAs. a List of inosine-modified crRNA sequences. Red [I] indicates the position of ribose inosine modifications in the crRNA sequence. **b** Graph showing the average specificity score for all non-modified positions vs. all modified positions for the indicated crRNAs. This value was calculated by averaging the specificity scores at each nucleotide position as visualized with heat maps in **Figure 2**. Specificity scores of 1.0 correspond to 100% enrichment for, while scores of −1.0 correspond to 100% enrichment against a base-pair at a specific position. These scores were averaged for all unmodified positions: (sum of specificity scores for each unmodified position in the crRNA) / (# of unmodified positions in the crRNA) = average unmodified specificity score (black). The average of all the modified positions was also calculated: (sum of specificity scores for each modified position in the crRNA) / (# of modified positions in the crRNA) = average modified specificity score (grey). The dotted horizontal line represents an average crRNA specificity score of 0.

a

[*] = Modification

crRNA: 5′-CAUGGAG[*]UCCGCGACCAC[*]-3′

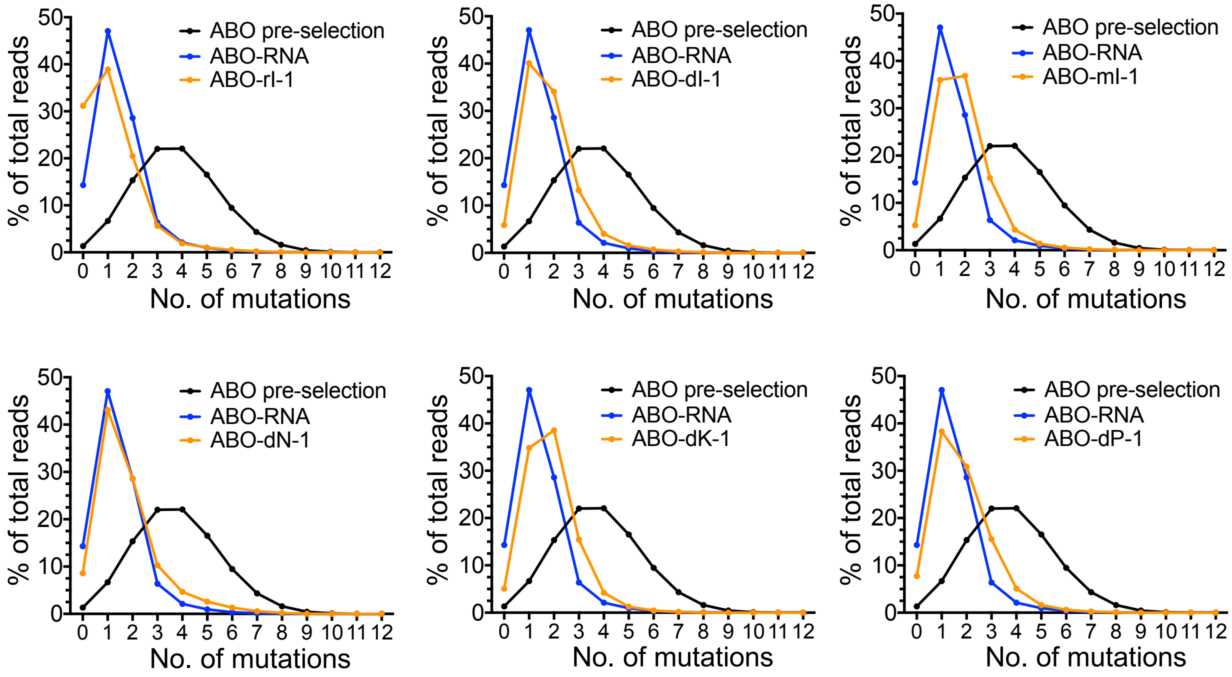

b

[*] = Modification

crRNA: 5′-CAUGGAG[*]UCCGCGACCA[*][*]-3′

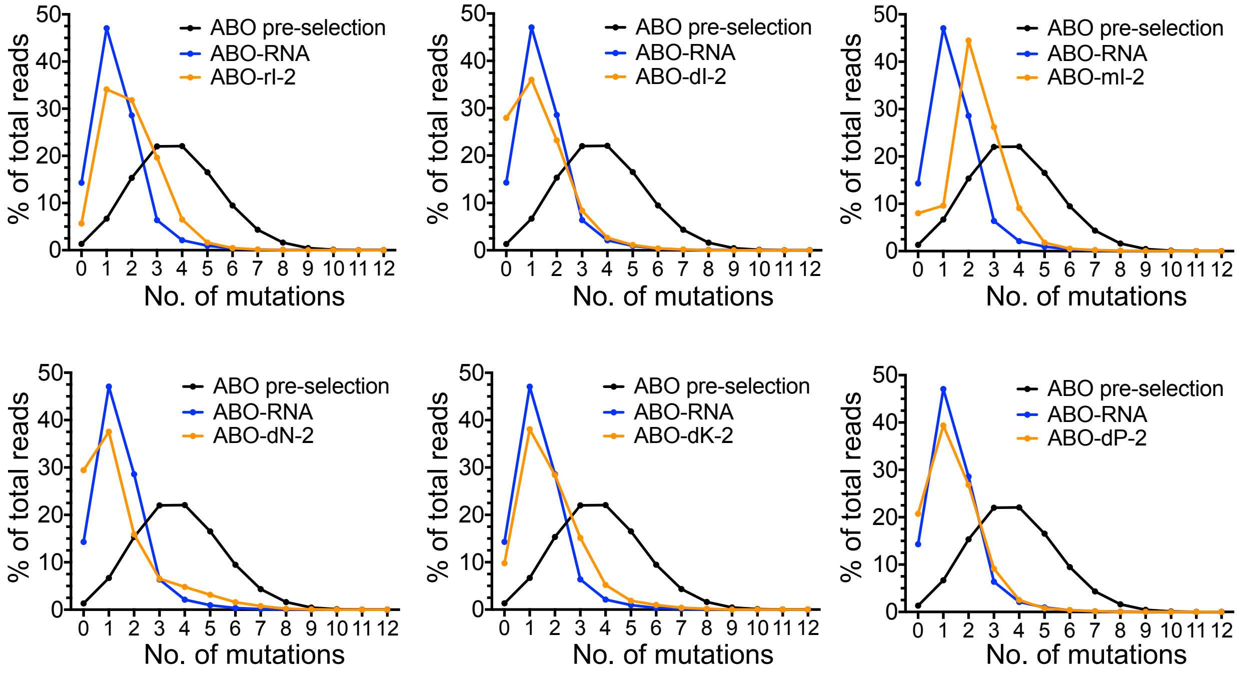

Supplementary Figure 12. Distribution of mutations in pre- and post-selection libraries for ABO-RNA and universal base-modified crRNAs. Graphs indicating the number of target sequence mutations in each pre- and post-selection library using crRNAs modified with universal bases at **a** two or **b** three positions, as indicated. ABO pre-selection (black) and ABO-RNA post-selection (blue) data are compared to the post-selection of crRNAs containing the indicated universal base(s) (orange). A red [*] denotes the position of the universal base modification in the crRNA sequence. Mutations were counted for each position throughout the 20 bp target site.

a

Target	DNA Sequence (5′→3′)	Allele Frequency (%)
**ABO-T1**	CATGGAGTTCCGCGACCACGTGG	54.21
**ABO-T2**	CATGGAGATCCGCGACCACGTGG	25.50
**ABO-T3**	CATGGAGTTCCGCGACCATGTGG	15.98
**ABO-T4**	CATGGAGTTCCGCGACCACATGG	0.015
**ABO-T5**	CATGGAGATCCGCGACCACATGG	0.0038
**ABO-T6**	CATGGAGATCCGCGACCATATGG	0.00061
**ABO-T7**	CATGGAGATCCGCGACCATATGG	4.08
**ABO-T8**	CATGGAGTTCCGCGACCATATGG	0.0024

b [*] = Modification

crRNA: 5′-CAUGGAG[*]UCCGCGACCAC[*]-3′

c [*] = Modification

crRNA: 5′-CAUGGAG[*]UCCGCGACCA[*][*]-3′

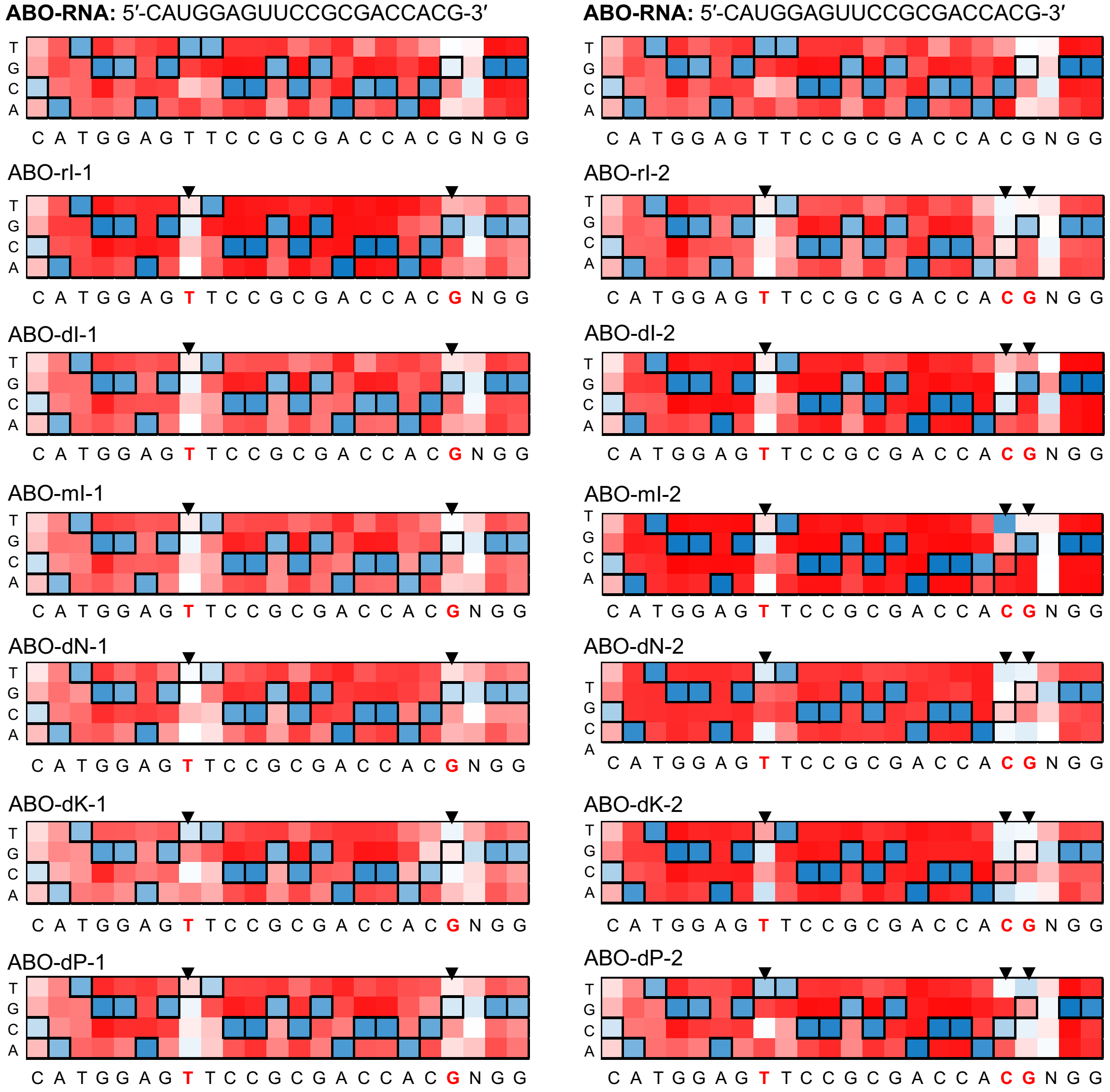

Supplementary Figure 13. ***In vitro*** **specificity profiles for** ***ABO*** **crRNAs containing various universal base modifications. a** List of DNA targets corresponding to sequences in the *ABO* gene based on clinical polymorphism data. SNPs are indicated with red lettering. The PAM sequence is underlined. Allele frequency indicates either the current tallied allele frequency or the statistically predicted frequency (for sequences containing multiple SNPs). Heat maps corresponding to the specificity profiles of crRNAs modified with universal bases at **b** two or **c** three positions, as indicated. The positions of universal bases are indicated by black arrows. Specificity scores of 1.0 (dark blue) correspond to 100% enrichment for, while scores of −1.0 (dark red) correspond to 100% enrichment against a base-pair at a specific position. Black boxes denote the intended target nucleotide.

a

[*] = Modification

crRNA: 5′-CAUGGAG[*]UCCGCGACCAC[*]-3′

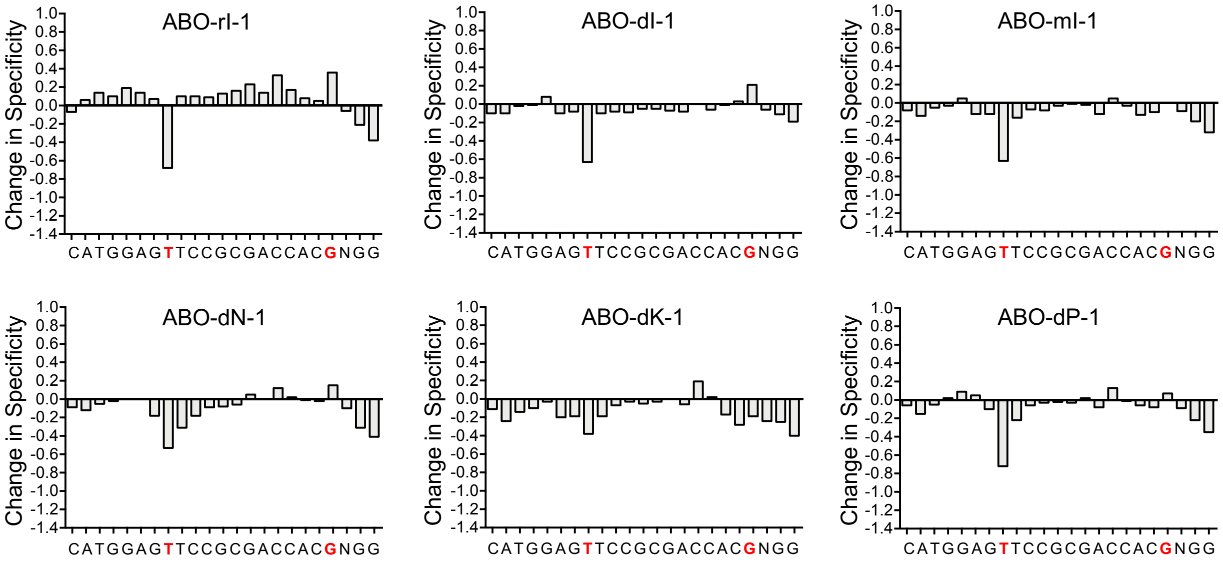

[*] = Modification

b

crRNA: 5′-CAUGGAG[*]UCCGCGACCA[*][*]-3′

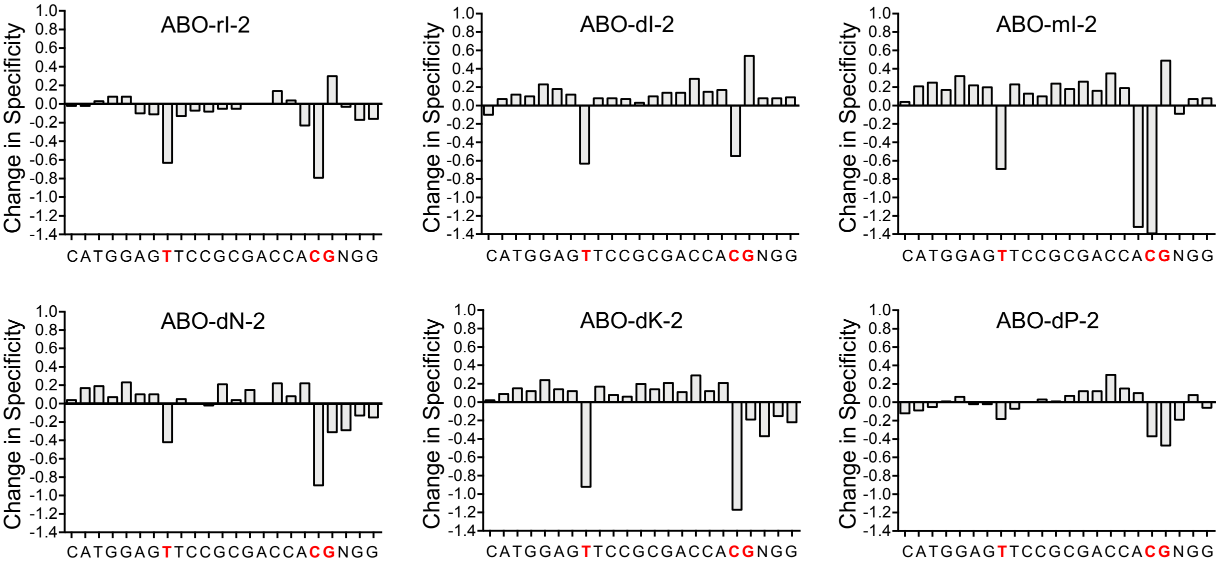

Supplementary Figure 14. Change in specificity score of universal base-modified crRNAs compared to ABO-RNA. Bar graphs showing the quantitative difference in specificity score at each position in the DNA target site for crRNAs modified with universal bases at **a** two or **b** three positions, as indicated. SNP locations in the 20 bp DNA target are indicated with red lettering. The PAM is shown as "NGG" on the 3′ end of the target. A score of zero indicates no change in specificity. The difference in specificity was calculated as the specificity score(modified)−specificity score(ABO-RNA). The specificity scoring of each nucleotide position is relative to the pre-selection control library data.

a

Name	crRNA Sequence (5′→3′)
**ABO-RNA**	CAUGGAGUUCCGCGACCACG
**ABO-[X]-1**	CAUGGAG[*]UCCGCGACCAC[*]
**ABO-[X]-2**	CAUGGAG[*]UCCGCGACCA[*][*]

b

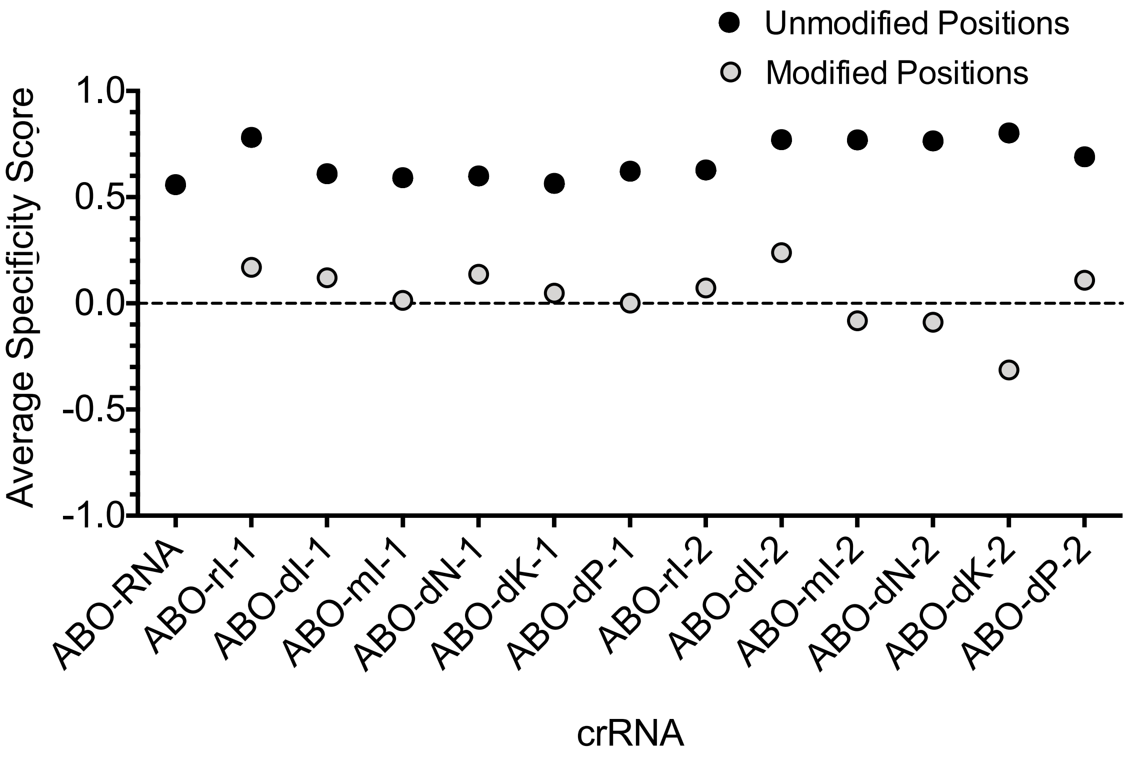

Supplementary Figure 15. Average specificity score of universal base modified positions in the *ABO* crRNAs. a List of univerasal base-modified crRNA sequences. Red [I] indicates the position of the universal base modifications in the crRNA sequence. **b** Graph showing the average specificity score for all non-modified positions vs. all modified positions for the indicated crRNAs. This value was calculated by averaging the specificity scores at each nucleotide position as visualized with heat maps in **Supplementary Figure 13**. Specificity scores of 1.0 correspond to 100% enrichment for, while scores of −1.0 correspond to 100% enrichment against a base-pair at a specific position. These scores were averaged for all unmodified positions: (sum of specificity scores for each unmodified position in the crRNA) / (# of unmodified positions in the crRNA) = average unmodified specificity score (black). The average of all the modified positions was also calculated: (sum of specificity scores for each modified position in the crRNA) / (# of modified positions in the crRNA) = average modified specificity score (grey). The dotted horizontal line represents an average crRNA specificity score of 0.

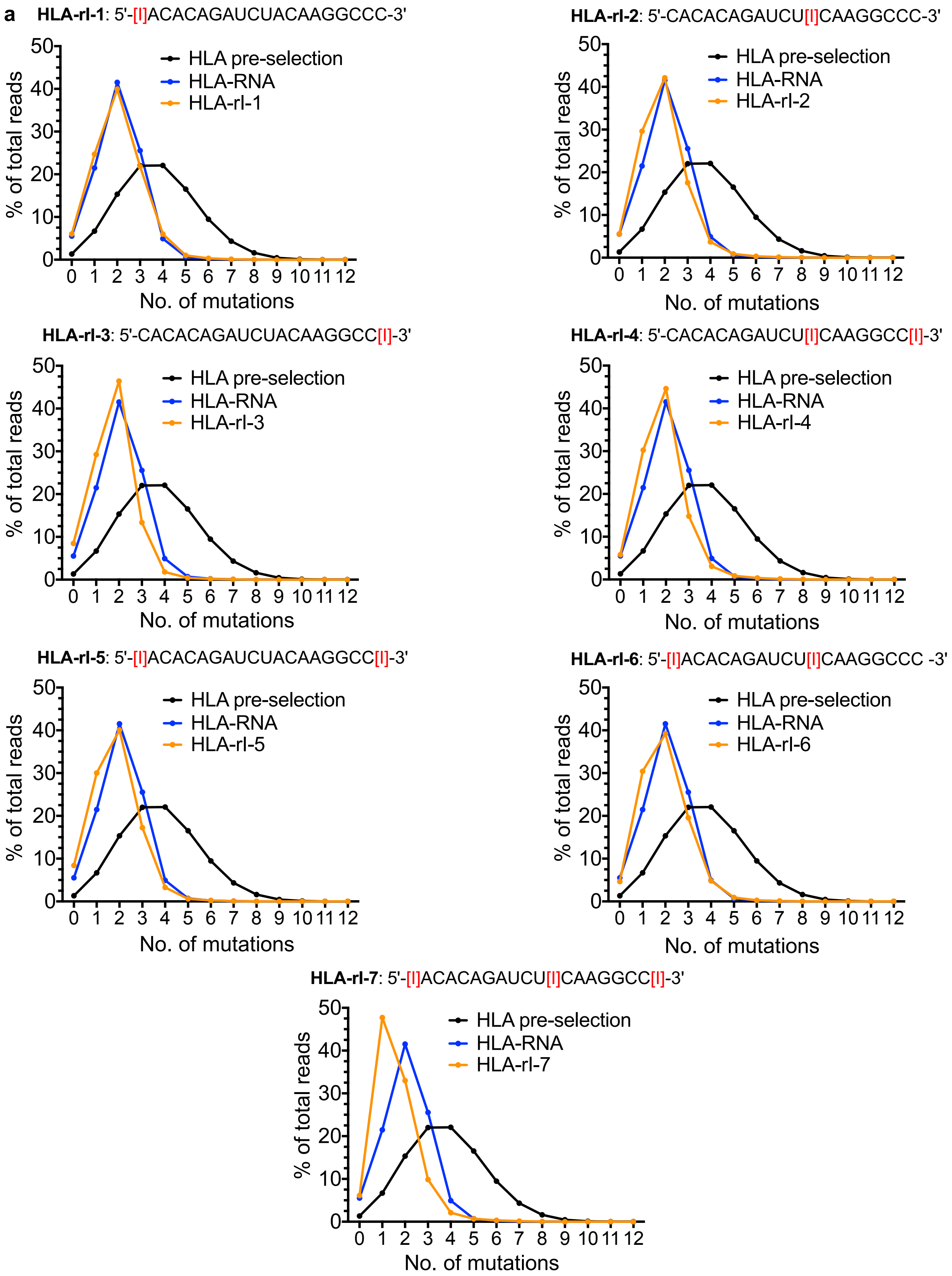
a
HLA-rl-1: 5'-[I]ACACAGAUCUACAAGGCCC-3'
HLA pre-selection
HLA-RNA
HLA-rl-1
% of total reads
No. of mutations
HLA-rl-2: 5'-CACACAGAUCU[I]CAAGGCCC-3'
HLA pre-selection
HLA-RNA
HLA-rl-2
HLA-rl-3: 5'-CACACAGAUCUACAAGGCC[I]-3'
HLA pre-selection
HLA-RNA
HLA-rl-3
HLA-rl-4: 5'-CACACAGAUCU[I]CAAGGCC[I]-3'
HLA pre-selection
HLA-RNA
HLA-rl-4
HLA-rl-5: 5'-[I]ACACAGAUCUACAAGGCC[I]-3'
HLA pre-selection
HLA-RNA
HLA-rl-5
HLA-rl-6: 5'-[I]ACACAGAUCU[I]CAAGGCCC -3'
HLA pre-selection
HLA-RNA
HLA-rl-6
HLA-rl-7: 5'-[I]ACACAGAUCU[I]CAAGGCC[I]-3'
HLA pre-selection
HLA-RNA
HLA-rl-7

Supplementary Figure 16. Distribution of mutations in pre- and post-selection libraries for HLA-RNA and inosine-modified crRNAs. a Graphs indicating the number of target sequence mutations in each pre- and post-selection library. HLA pre-selection (black) and HLA-RNA post-selection (blue) data are compared to the post-selection of crRNAs containing ribose inosine(s) (orange). Red [I] denotes the position of inosine modifications in the crRNA sequence*.* Mutations were counted for each position throughout the 20 bp target site.

a

Target	DNA Sequence (5′→3′)	Allele Frequency (%)
**HLA-T1**	CACACAGATCTACAAGGCCC**AGG**	0.50
**HLA-T2**	**G**ACACAGATCTACAAGGCCC**AGG**	45.49
**HLA-T3**	CACACAGATCT**C**CAAGGCCC**AGG**	36.70
**HLA-T4**	CACACAGATCTACAAGGCC**A**AGG	74.20
**HLA-T5**	CACACAGATCT**C**CAAGGCC**A**AGG	27.23
**HLA-T6**	**G**ACACAGATCTACAAGGCC**A**AGG	33.75
**HLA-T7**	**G**ACACAGATCT**C**CAAGGCCC**AGG**	16.69
**HLA-T8**	**G**ACACAGATCT**C**CAAGGCC**A**AGG	12.39

b

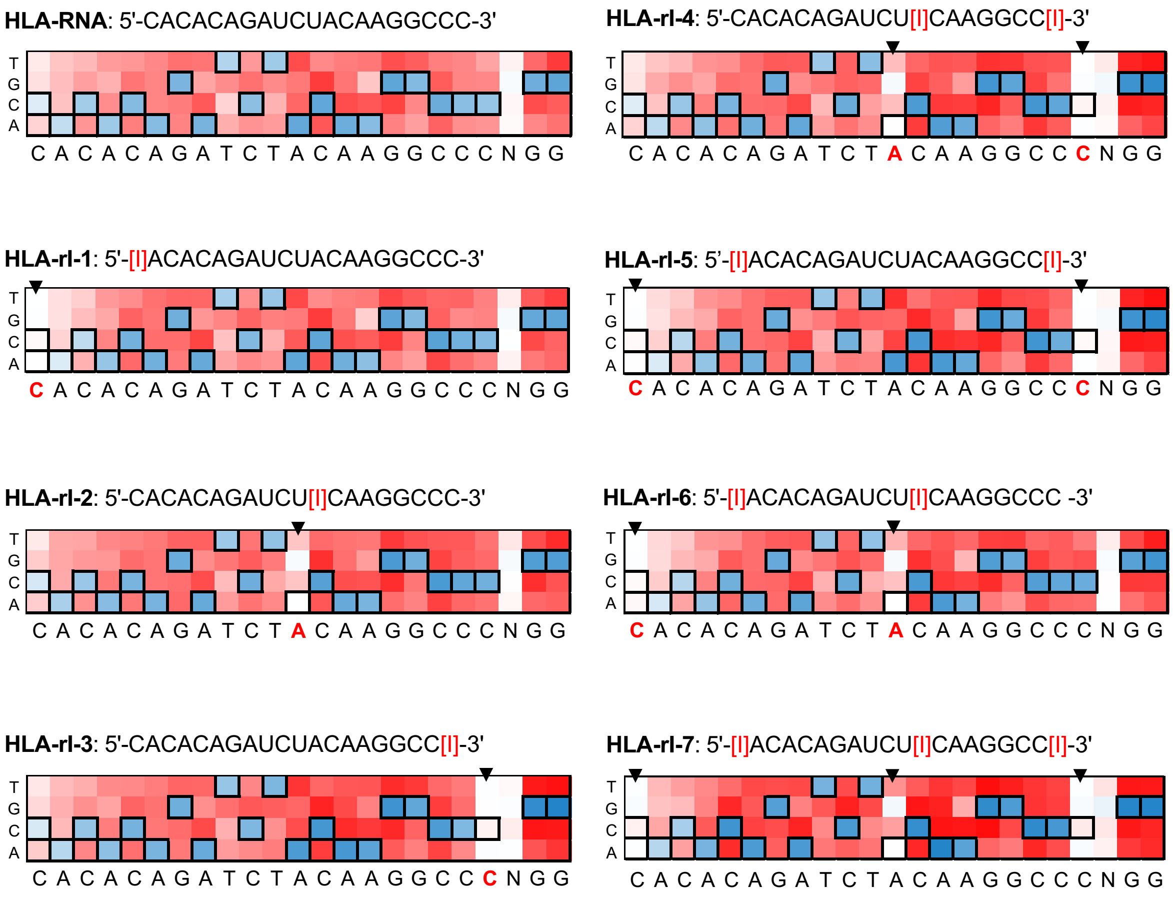

Supplementary Figure 17. ***In vitro*** **specificity profiles for** ***HLA*** **crRNAs containing inosine base modifications. a** List of DNA targets corresponding to sequences in the *HLA* gene based on clinical polymorphism data. SNPs are indicated with red lettering. The PAM sequence is underlined. Allele frequency indicates either the current tallied allele frequency or the statistically predicted frequency (for sequences containing multiple SNPs). **b** Heat maps corresponding to the specificity profiles of crRNAs modified with inosine bases at the indicated positions. The positions of inosine bases are indicated by black arrows. Specificity scores of 1.0 (dark blue) correspond to 100% enrichment for, while scores of −1.0 (dark red) correspond to 100% enrichment against a base-pair at a specific position. Black boxes denote the intended target nucleotide.

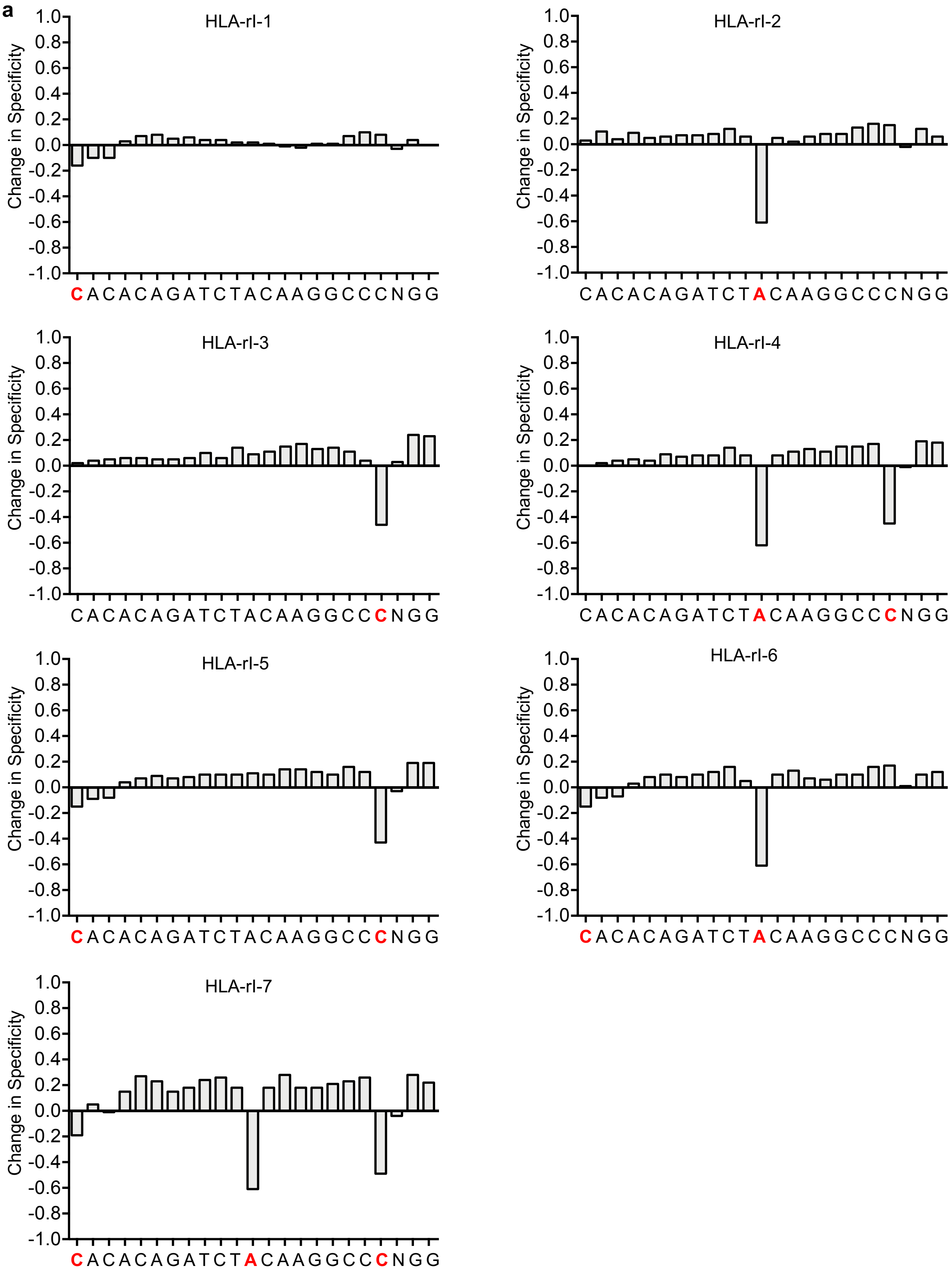
a
HLA-rl-1
HLA-rl-2
HLA-rl-3
HLA-rl-4
HLA-rl-5
HLA-rl-6
HLA-rl-7
Change in Specificity
1.0
0.8
0.6
0.4
0.2
0.0
-0.2
-0.4
-0.6
-0.8
-1.0
CACACAGATCTACAAGGCCCNGG

Supplementary Figure 18. Change in specificity score of inosine-modified crRNAs compared to HLA-RNA. a Bar graphs showing the quantitative difference in specificity score at each position in the DNA target site for crRNAs modified with inosine bases as indicated. SNP locations in the 20 base-pair DNA target are indicated with red lettering. The PAM is shown as “NGG” on the 3′ end of the target. A score of zero indicates no change in specificity. The difference in specificity was calculated as the specificity score(modified)−specificity score(HLA-RNA). The specificity scoring of each nucleotide position is relative to the pre-selection control library data.

a

Name	crRNA Sequence (5′→3′)
**HLA-RNA**	CACACAGAUCUACAAGGCCC
**HLA-rI-1**	[I]ACACAGAUCUACAAGGCCC
**HLA-rI-2**	CACACAGAUCU[I]CAAGGCCC
**HLA-rI-3**	CACACAGAUCUACAAGGCC[I]
**HLA-rI-4**	CACACAGAUCU[I]CAAGGCC[I]
**HLA-rI-5**	[I]ACACAGAUCUACAAGGCC[I]
**HLA-rI-6**	[I]ACACAGAUCU[I]CAAGGCCC
**HLA-rI-7**	[I]ACACAGAUCU[I]CAAGGCC[I]

b

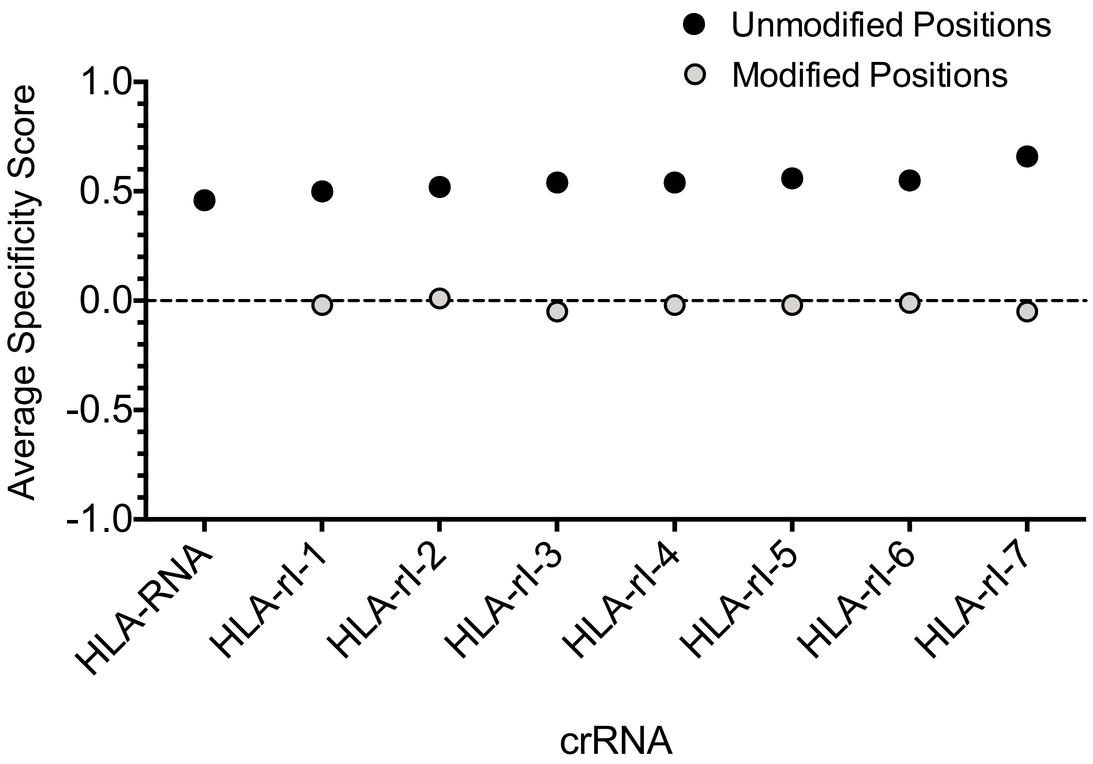

Supplementary Figure 19. Average specificity score of ribose inosine modified positions in the *HLA* crRNAs. a List of inosine-modified crRNA sequences. Red [I] indicates the position of ribose inosine modifications in the crRNA sequence. **b** Graph showing the average specificity score for all non-modified positions vs. all modified positions for the indicated crRNAs. This value was calculated by averaging the specificity scores at each nucleotide position as visualized with heat maps in **Supplementary Figure 17**. Specificity scores of 1.0 correspond to 100% enrichment for, while scores of −1.0 correspond to 100% enrichment against a base-pair at a specific position. These scores were averaged for all unmodified positions: (sum of specificity scores for each unmodified position in the crRNA) / (# of unmodified positions in the crRNA) = average unmodified specificity score (black). The average of all the modified positions was also calculated: (sum of specificity scores for each modified position in the crRNA) / (# of modified positions in the crRNA) = average modified specificity score (grey). The dotted horizontal line represents an average crRNA specificity score of 0.

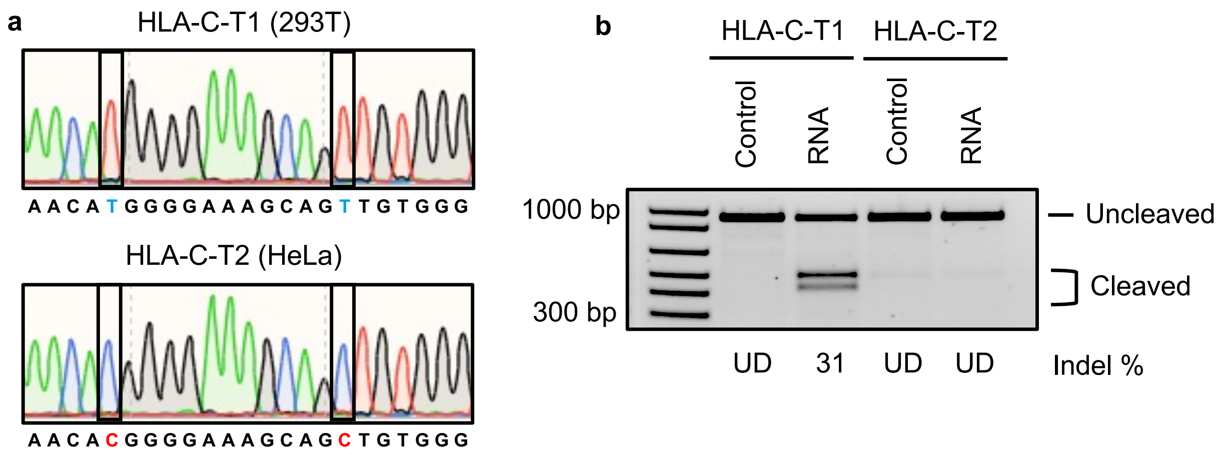

c

Name	crRNA Sequence (5′→3′)
**HLA-C-RNA**	AACAUGGGGAAAGCAGUUGU
**HLA-C-rI**	AACA[rI]GGGGAAAGCAG[rI]UGU
**HLA-C-dI**	AACA[dI]GGGGAAAGCAG[dI]UGU
**HLA-C-dP**	AACA[dP]GGGGAAAGCAG[dP]UGU

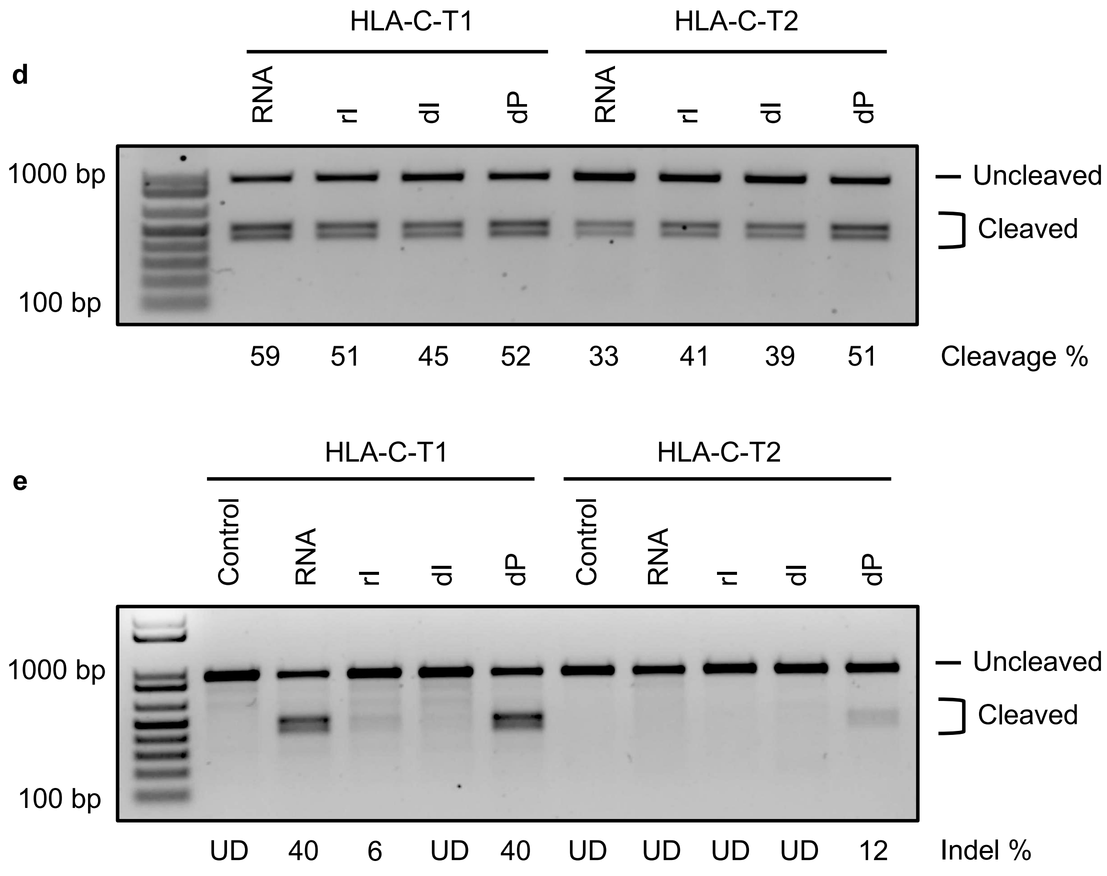

Supplementary Figure 20. Cas9 cleavage activity using HLA-C-RNA and universal base-modified crRNAs *in vitro* and on the endogenous *HLA-C* locus in 293T and HeLa cells. a Sanger sequencing trace of endogenous SNPs present within the *HLA-C* locus of 293T and HeLa cells. **b** Gel showing the cellular Cas9 cleavage efficiencies of HLA-C-T1 or HLA-C-T2 sequences using an umodified HLA-C-RNA crRNA as determined by T7 endonuclease I digestion. Controls were harvested from cells stably expressing Cas9 that were not transfected with a guide RNA. **c** List of universal base-modified crRNA sequences. Red [X] indicates the position of the modified base within the crRNA sequence. **d** Gel showing the relative *in vitro* cleavage efficiencies of Cas9 RNPs complexed with HLA-C-RNA or universal base-modified crRNAs against the HLA-C-T1 or HLA-C-T2 DNA targets. **e** Gel showing the relative cellular cleavage efficiences of Cas9 against HLA-C-T1 or HLA-C-T2 when complexed with HLA-C-RNA or universal base-modified crRNAs as determined by T7 endonuclease I digestion. Controls were harvested from cells stably expressing Cas9 that were not transfected with a guide RNA. UD denotes undetectable. Both *in vitro* and cellular cleavage assays of HLA-C-T1/T2 were performed in duplicate with similar results.

a

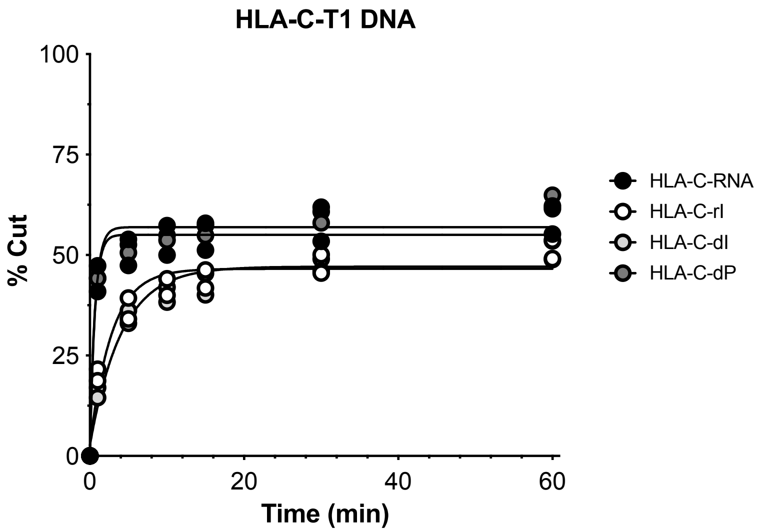

b

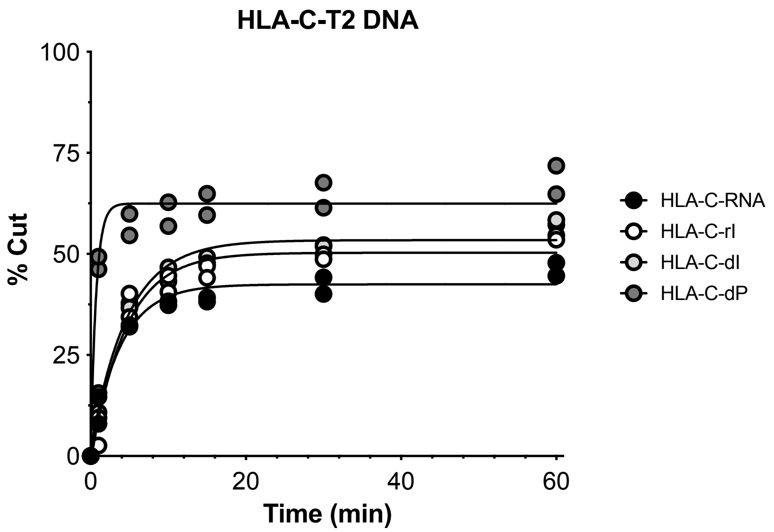

Supplementary Figure 21. ***In vitro*** **Cas9 cleavage kinetics using HLA-C-RNA or universal base-modified crRNAs.** Time course showing *in vitro* cleavage activity of Cas9 on **a** HLA-C-T1 or **b** HLA-C-T2 DNA targets using HLA-C-RNA or the indicated universal base-modified crRNAs. Experiments were performed with a concentration of 10 nM Cas9, 20 nM gRNA and 5 nM DNA target; Individual data points shown (n = 2 independent experiments).

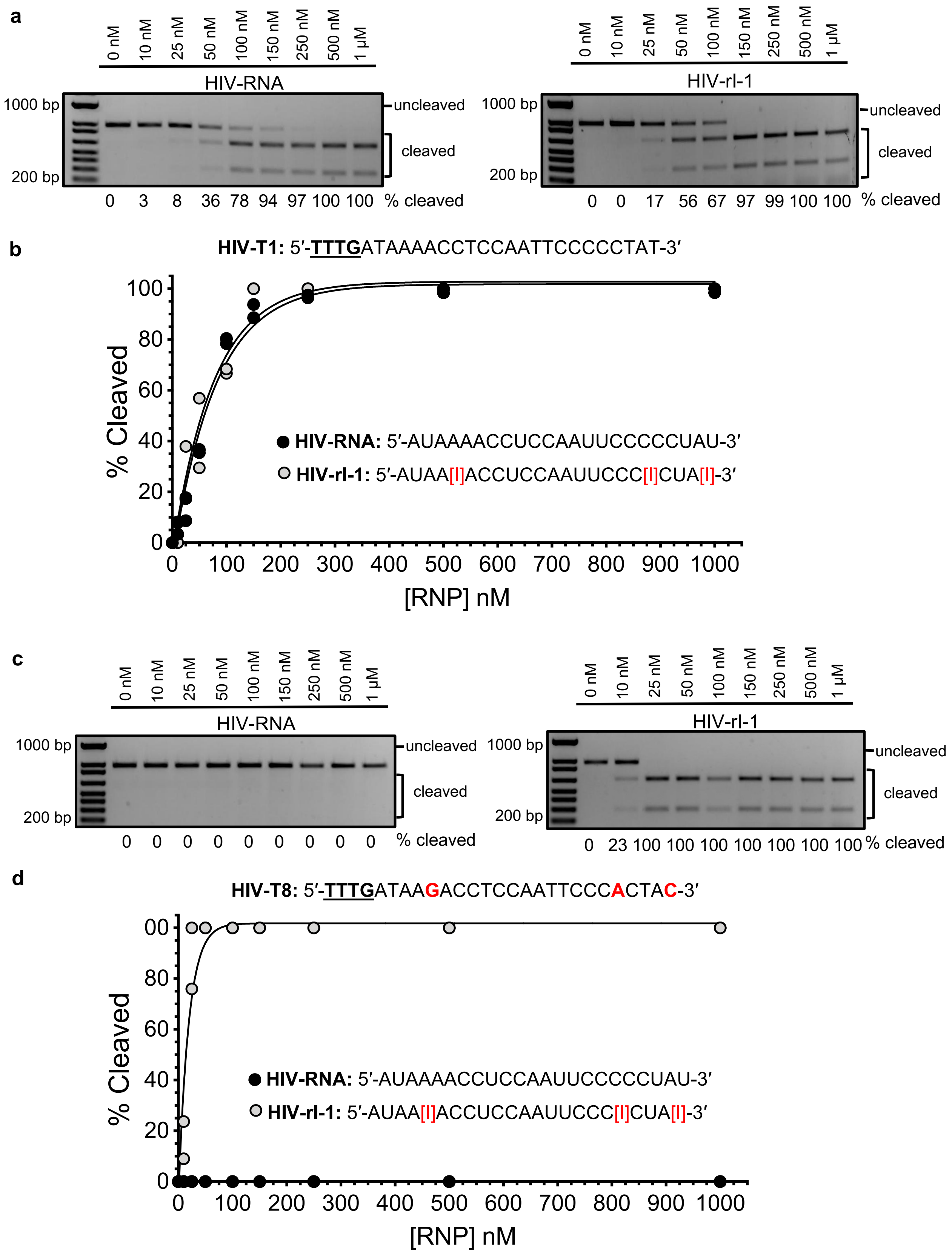
a
0 nM
10 nM
25 nM
50 nM
100 nM
150 nM
250 nM
500 nM
1 μM
HIV-RNA
1000 bp
200 bp
uncleaved
cleaved
0 3 8 36 78 94 97 100 100 % cleaved
HIV-rI-1
0 0 17 56 67 97 99 100 100 % cleaved
b
HIV-T1: 5′-TTTGATAAAACCTCCAATTCCCCCTAT-3′
% Cleaved
[RNP] nM
HIV-RNA: 5′-AUAAAACCUCCAAUUCCCCCUAU-3′
HIV-rI-1: 5′-AUAA[I]ACCUCCAAUUCCC[I]CUA[I]-3′
c
0 0 0 0 0 0 0 0 0 % cleaved
0 23 100 100 100 100 100 100 100 % cleaved
d
HIV-T8: 5′-TTTGATAAGACCTCCAATTCCCACTAC-3′

Supplementary Figure 22. Titration of Cas12a RNP containing HIV-RNA or HIV-rI-1 against select target sequences *in vitro*. a Representative gels showing a titration of Cas12a RNP containing HIV-RNA or HIV-rI-1 crRNAs against the HIV-T1 target sequence. The bottom two bands in the gel represent the cleaved DNA substrate while the top band corresponds to the undigested substrate. **b** Plot quantifying the results of experiments in **a.** DNA target sequences with SNPs are indicated with red lettering. **[I]** in the crRNA sequence indicates a ribose inosine position. Quantification of cleavage percentages was performed using ImageJ; Individual data points shown (n = 2 independent experiments). **c** Representative gels showing a titration of Cas12a RNP containing HIV-RNA or HIV-rI-1 crRNAs against the HIV-T8 target sequence. The bottom two bands in the gel represent the cleaved DNA substrate while the top band corresponds to the undigested substrate. **d** Plot quantifying the results of experiments in **c.** DNA target sequences with SNPs are indicated with red lettering. Red **[I]** in the crRNA sequence indicates a ribose inosine position. Quantification of cleavage percentages was performed using ImageJ; Individual data points shown (n = 2 independent experiments). Cleavage experiments were performed in duplicate with similar results.

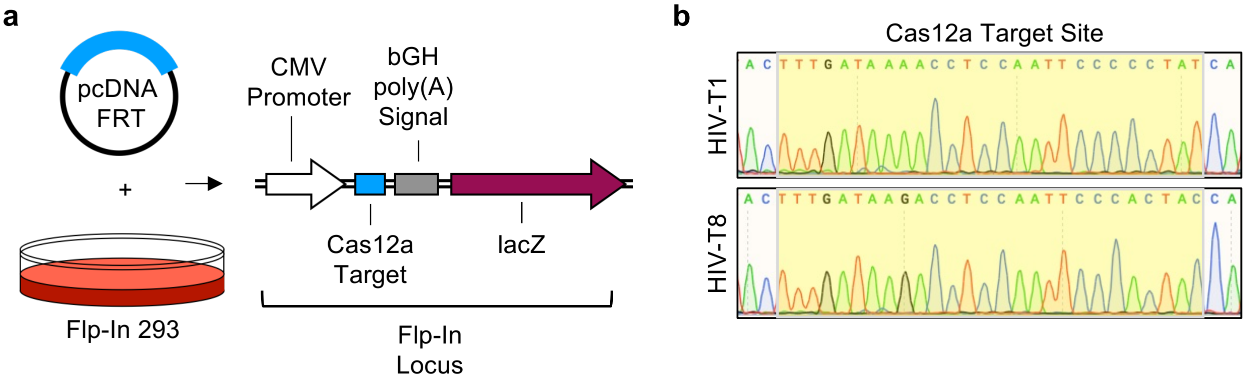

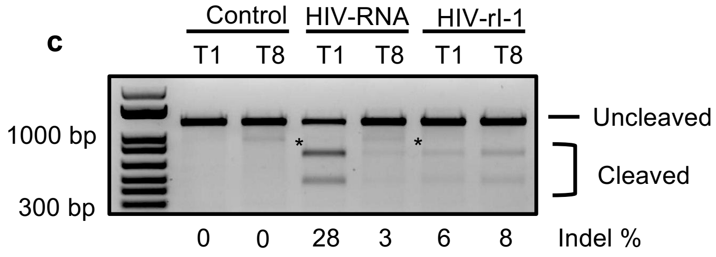

d

Target	No. of Mismatches	Sequence
**HIV-T1**	0	ATAAAACCTCCAATTCCCCCTAT
**HIV-OT1**	4	ATccAcCaTCCAATTCCCCCTAT
**HIV-OT2**	4	ATAAAACCTCtAATTCCataTAT

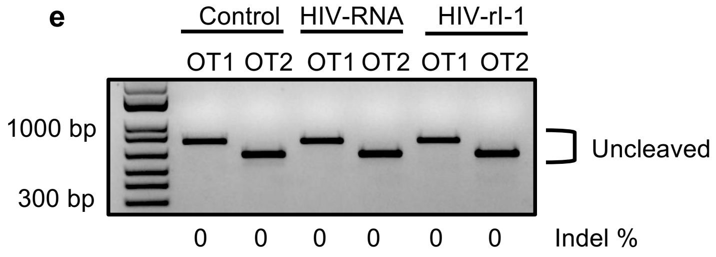

Supplementary Figure 23. Cleavage of HIV target sequences by Cas12a with unmodified and inosine modified crRNAs in cells. a Diagram illustrating the generation of Flp-In 293 cells containing single-copy genomic HIV DNA target sequences. **b** Sanger sequencing trace confirming DNA target integration. **c** Gel representing cellular Cas12a cleavage efficiencies of the HIV-T1 or HIV-T8 sequences using either the unmodified or ribose inosine-modified HIV crRNAs, as determined by T7 endonuclease I digestion. Control transfections were performed without crRNAs. Indel percentages were determined using densitometry (ImageJ) and are shown below each lane. **d** Potential genomic off-target sequences (OT1: Chr17:40142160, OT2: Chr1:213961893) corresponding to the HIV target sequence (HIV-T1) predicted using Cas-OFFinder ((http://www.rgenome.net/cas-offinder/)[1]. **e** Gel representing cellular Cas12a cleavage efficiencies of the OT1 or OT2 sequences using either the unmodified or ribose inosine-modified *HIV* crRNAs, as determined by T7 endonuclease I digestion. Control transfections were performed without crRNAs. Indel percentages were determined using densitometry (ImageJ) and are shown below each lane. Cellular cleavage experiments were performed in duplicate with similar results.

Supplementary Table 1. Sequences of crRNAs and tracrRNA. [rI] = Ribose inosine, [dI] = Deoxyribose inosine, [mI] = 2′O methyl ribose inosine, [dN] = Deoxyribose 5′-nitroindole, [dK] = Deoxyribose K, [dP] = Deoxyribose P, and /Cy5/ = Cyanine 5.

Name	Sequence (5′→3′)
HLA-RNA	rCrArCrArCrArGrArUrCrUrArCrArArGrGrCrCrCrGrUrUrUrArGrArGr CrUrArUrGrCrU
HLA-rI-1	[rI]rArCrArCrArGrArUrCrUrArCrArArGrGrCrCrCrGrUrUrUrArGrArGr CrUrArUrGrCrU
HLA-rI-2	rCrArCrArCrArGrArUrCrU[rI]rCrArArGrGrCrCrCrGrUrUrUrArGrArGr CrUrArUrGrCrU
HLA-rI-3	rCrArCrArCrArGrArUrCrUrArCrArArGrGrCrC[rI]rGrUrUrUrArGrArGr CrUrArUrGrCrU
HLA-rI-4	rCrArCrArCrArGrArUrCrU[rI]rCrArArGrGrCrC[rI]rGrUrUrUrArGrArG rCrUrArUrGrCrU
HLA-rI-5	[rI]rArCrArCrArGrArUrCrUrArCrArArGrGrCrC[rI]rGrUrUrUrArGrArGr CrUrArUrGrCrU
HLA-rI-6	[rI]rArCrArCrArGrArUrCrU[rI]rCrArArGrGrCrCrCrGrUrUrUrArGrArG rCrUrArUrGrCrU
HLA-rI-7	[rI]rArCrArCrArGrArUrCrU[rI]rCrArArGrGrCrC[rI]rGrUrUrUrArGrArG rCrUrArUrGrCrU
ABO-RNA	rCrArUrGrGrArGrUrUrCrCrGrCrGrArCrCrArCrGrGrUrUrUrArGrArG rCrUrArUrGrCrU
ABO-rI-1	rCrArUrGrGrArG[rI]rUrCrCrGrCrGrArCrCrArC[rI]rGrUrUrUrArGrArG rCrUrArUrGrCrU
ABO-dI-1	rCrArUrGrGrArG[dI]rUrCrCrGrCrGrArCrCrArC[dI]rGrUrUrUrArGrAr GrCrUrArUrGrCrU
ABO-mI-1	rCrArUrGrGrArG[mI]rUrCrCrGrCrGrArCrCrArC[mI]rGrUrUrUrArGrA rGrCrUrArUrGrCrU
ABO-dN-1	rCrArUrGrGrArG[5NitInd]rUrCrCrGrCrGrArCrCrArC[5NitInd]rGrUrU rUrArGrArGrCrUrArUrGrCrU
ABO-dK-1	rCrArUrGrGrArG[dK]rUrCrCrGrCrGrArCrCrArC[dK]rGrUrUrUrArGr ArGrCrUrArUrGrCrU
ABO-dP-1	rCrArUrGrGrArG[dP]rUrCrCrGrCrGrArCrCrArC[dP]rGrUrUrUrArGr ArGrCrUrArUrGrCrU
ABO-rI-2	rCrArUrGrGrArG[rI]rUrCrCrGrCrGrArCrCrA[rI][rI]rGrUrUrUrArGrAr GrCrUrArUrGrCrU
ABO-dI-2	rCrArUrGrGrArG[dI]rUrCrCrGrCrGrArCrCrA[dI][dI]rGrUrUrUrArGrAr GrCrUrArUrGrCrU
ABO-mI-2	rCrArUrGrGrArG[mI]rUrCrCrGrCrGrArCrCrA[mI][mI]rGrUrUrUrArGr ArGrCrUrArUrGrCrU
ABO-dN-2	rCrArUrGrGrArG[5NitInd]rUrCrCrGrCrGrArCrCrA[5NitInd][5NitInd]rGr UrUrUrArGrArGrCrUrArUrGrCrU

ABO-dK-2	rCrArUrGrGrArG[dK]rUrCrCrGrCrGrArCrCrA[dK][dK]rGrUrUrUrUrArG rArGrCrUrArUrGrCrU
ABO-dP-2	rCrArUrGrGrArG[dP]rUrCrCrGrCrGrArCrCrA[dP][dP]rGrUrUrUrUrArG rArGrCrUrArUrGrCrU
ABO-rI-3	rCrArUrGrGrArG[rI]rUrCrCrGrCrGrArCrCrArCrGrGrUrUrUrUrArGrArG rCrUrArUrGrCrU
ABO-rI-4	rCrArUrGrGrArGrUrUrC[rI]rGrCrGrArCrCrArCrGrGrUrUrUrUrArGrArG rCrUrArUrGrCrU
ABO-rI-5	rCrArUrGrGrArGrUrUrCrCrGrCrGrArCrCrA[rI]rGrGrUrUrUrUrArGrArG rCrUrArUrGrCrU
ABO-rI-6	rCrArUrGrGrArGrUrUrCrCrGrCrGrArCrCrArC[rI]rGrUrUrUrUrArGrArG rCrUrArUrGrCrU
ABO-rI-7	rCrArUrGrGrArG[rI]rUrC[rI]rGrCrGrArCrCrArCrGrGrUrUrUrUrArGrArG rCrUrArUrGrCrU
ABO-rI-8	rCrArUrGrGrArG[rI]rUrCrCrGrCrGrArCrCrA[rI]rGrGrUrUrUrUrArGrArG rCrUrArUrGrCrU
ABO-rI-9	rCrArUrGrGrArGrUrUrC[rI]rGrCrGrArCrCrA[rI]rGrGrUrUrUrUrArGrArG rCrUrArUrGrCrU
ABO-rI-10	rCrArUrGrGrArGrUrUrC[rI]rGrCrGrArCrCrArC[rI]rGrUrUrUrUrArGrArG rCrUrArUrGrCrU
ABO-rI-11	rCrArUrGrGrArGrUrUrCrCrGrCrGrArCrCrA[rI][rI]rGrUrUrUrUrArGrArG rCrUrArUrGrCrU
ABO-rI-12	rCrArUrGrGrArG[rI]rUrC[rI]rGrCrGrArCrCrA[rI]rGrGrUrUrUrUrArGrAr GrCrUrArUrGrCrU
ABO-rI-13	rCrArUrGrGrArG[rI]rUrC[rI]rGrCrGrArCrCrArC[rI]rGrUrUrUrUrArGrAr GrCrUrArUrGrCrU
ABO-rI-14	rCrArUrGrGrArGrUrUrC[rI]rGrCrGrArCrCrA[rI][rI]rGrUrUrUrUrArGrAr GrCrUrArUrGrCrU
ABO-rI-15	rCrArUrGrGrArG[rI]rUrC[rI]rGrCrGrArCrCrA[rI][rI]rGrUrUrUrUrArGrAr GrCrUrArUrGrCrU
ABO-Negative	rGrArGrUrCrCrGrArGrCrArGrArArGrArArGrArArGrUrUrUrUrArGrArGr CrUrArUrGrCrU
HLA-C-RNA	rArArCrArUrGrGrGrGrArArArGrCrArGrUrUrGrUrGrUrUrUrUrArGrArGr CrUrArUrGrCrU
HLA-C-rI	rArArCrA[rI]rGrGrGrGrArArArGrCrArG[rI]rUrGrUrGrUrUrUrUrArGrArG rCrUrArUrGrCrU
HLA-C-dI	rArArCrA[dI]rGrGrGrGrArArArGrCrArG[dI]rUrGrUrGrUrUrUrUrArGrAr GrCrUrArUrGrCrU
HLA-C-dP	rArArCrA[dP]rGrGrGrGrArArArGrCrArG[dP]rUrGrUrGrUrUrUrUrArGr ArGrCrUrArUrGrCrU
HIV-RNA	rUrArArUrUrUrCrUrArCrUrCrUrUrGrUrArGrArUrArUrArArArArCrCrUr CrCrArArUrUrCrCrCrCrCrUrArU

HIV-rI-1	rUrArArUrUrUrCrUrArCrUrCrUrUrGrUrArGrArUrArUrArA[rI]rArCrCrUr CrCrArArUrUrCrCrC[rI]rCrUrA[rI]
tracrRNA	rArGrCrArUrArGrCrArArGrUrUrArArArArUrArArGrGrCrUrArGrUrCrCr GrUrUrArUrCrArArCrUrUrGrArArArArArGrUrGrGrCrArCrCrGrArGrUr CrGrGrUrGrCrUrUrU

Supplementary Table 2. Sequences of oligonucleotides.

Name	Sequences (5′→3′)
Oligos used to create *in vitro* cleavage assay DNA target constructs.	
ABO-T1-F	GCCGAAGCTTCTCCACGTGGTCGCGGAACTCCATGCTTCTA GAGGCC
ABO-T1-R	GGCCTCTAGAAGCATGGAGTTCCGCGACCACGTGGAGAAGC TTCGGC
ABO-T2-F	GCCGAAGCTTCTCCACGTGGTCGCGGATCTCCATGCTTCTA GAGGCC
ABO-T2-R	GGCCTCTAGAAGCATGGAGATCCGCGACCACGTGGAGAAG CTTCGGC
ABO-T3-F	GCCGAAGCTTCTCCACATGGTCGCGGAACTCCATGCTTCTA GAGGCC
ABO-T3-R	GGCCTCTAGAAGCATGGAGTTCCGCGACCATGTGGAGAAGC TTCGGC
ABO-T4-F	GCCGAAGCTTCTCCATGTGGTCGCGGAACTCCATGCTTCTA GAGGCC
ABO-T4-R	GGCCTCTAGAAGCATGGAGTTCCGCGACCACATGGAGAAGC TTCGGC
ABO-T5-F	GCCGAAGCTTCTCCATGTGGTCGCGGATCTCCATGCTTCTA GAGGCC
ABO-T5-R	GGCCTCTAGAAGCATGGAGATCCGCGACCACATGGAGAAGC TTCGGC
ABO-T6-F	GCCGAAGCTTCTCCATATGGTCGCGGATCTCCATGCTTCTAG AGGCC
ABO-T6-R	GGCCTCTAGAAGCATGGAGATCCGCGACCATATGGAGAAGC TTCGGC
ABO-T7-F	GCCGAAGCTTCTCCACATGGTCGCGGATCTCCATGCTTCTA GAGGCC
ABO-T7-R	GGCCTCTAGAAGCATGGAGATCCGCGACCATGTGGAGAAGC TTCGGC
ABO-T8-F	GCCGAAGCTTCTCCATATGGTCGCGGAACTCCATGCTTCTA GAGGCC
ABO-T8-R	GGCCTCTAGAAGCATGGAGTTCCGCGACCATATGGAGAAGC TTCGGC

ABO-T9-F	GCCGAAGCTTCTCCACGTGGTCGCAGAACTCCATGCTTCTA GAGGCC
ABO-T9-R	GGCCTCTAGAAGCATGGAGTTCTGCGACCACGTGGAGAAGC TTCGGC
ABO-T10-F	GCCGAAGCTTCTCCACGTGGTCGCAGATCTCCATGCTTCTA GAGGCC
ABO-T10-R	GGCCTCTAGAAGCATGGAGATCTGCGACCACGTGGAGAAGC TTCGGC
ABO-T11-F	GCCGAAGCTTCTCCACATGGTCGCAGAACTCCATGCTTCTA GAGGCC
ABO-T11-R	GGCCTCTAGAAGCATGGAGTTCTGCGACCATGTGGAGAAGC TTCGGC
ABO-T12-F	GCCGAAGCTTCTCCATGTGGTCGCAGAACTCCATGCTTCTA GAGGCC
ABO-T12-R	GGCCTCTAGAAGCATGGAGTTCTGCGACCACATGGAGAAGC TTCGGC
ABO-T13-F	GCCGAAGCTTCTCCACATGGTCGCAGATCTCCATGCTTCTAG AGGCC
ABO-T13-R	GGCCTCTAGAAGCATGGAGATCTGCGACCATGTGGAGAAGC TTCGGC
ABO-T14-F	GCCGAAGCTTCTCCATGTGGTCGCAGATCTCCATGCTTCTAG AGGCC
ABO-T14-R	GGCCTCTAGAAGCATGGAGATCTGCGACCACATGGAGAAGC TTCGGC
ABO-T15-F	GCCGAAGCTTCTCCATATGGTCGCAGAACTCCATGCTTCTAG AGGCC
ABO-T15-R	GGCCTCTAGAAGCATGGAGTTCTGCGACCATATGGAGAAGC TTCGGC
ABO-T16-F	GCCGAAGCTTCTCCATATGGTCGCAGATCTCCATGCTTCTAG AGGCC
ABO-T16-R	GGCCTCTAGAAGCATGGAGATCTGCGACCATATGGAGAAGC TTCGGC
ABO-T5 (G-G)-F	GCC GAA GCT TCT CCA CGT GGT CGC GGA CCT CCA TGC TTC TAG AGG CC
ABO-T5 (G-G)-R	GGC CTC TAG AAG CAT GGA GGT CCG CGA CCA CGT GGA GAA GCT TCG GC
ABO-T5 (G-C)-F	GCC GAA GCT TCT CCA CGT GGT CGC GGA GCT CCA TGC TTC TAG AGG CC
ABO-T5 (G-C)-R	GGC CTC TAG AAG CAT GGA GCT CCG CGA CCA CGT GGA GAA GCT TCG GC
ABO-T5 (G-T)-F	GCC GAA GCT TCT CCA CGT GGT CGC GGA ACT CCA TGC TTC TAG AGG CC
ABO-T5 (G-T)-R	GGC CTC TAG AAG CAT GGA GTT CCG CGA CCA CGT GGA GAA GCT TCG GC

ABO-T5 (G-A)-F	GCC GAA GCT TCT CCA CGT GGT CGC GGA TCT CCA TGC TTC TAG AGG CC
ABO-T5 (G-A)-R	GGC CTC TAG AAG CAT GGA GAT CCG CGA CCA CGT GGA GAA GCT TCG GC
ABO-T5 (C-G)-F	GCC GAA GCT TCT CCA GGT GGT CGC GGA CCT CCA TGC TTC TAG AGG CC
ABO-T5 (C-G)-R	GGC CTC TAG AAG CAT GGA GGT CCG CGA CCA CCT GGA GAA GCT TCG GC
ABO-T5 (C-C)-F	GCC GAA GCT TCT CCA GGT GGT CGC GGA GCT CCA TGC TTC TAG AGG CC
ABO-T5 (C-C)-R	GGC CTC TAG AAG CAT GGA GCT CCG CGA CCA CCT GGA GAA GCT TCG GC
ABO-T5 (C-T)-F	GCC GAA GCT TCT CCA GGT GGT CGC GGA ACT CCA TGC TTC TAG AGG CC
ABO-T5 (C-T)-R	GGC CTC TAG AAG CAT GGA GTT CCG CGA CCA CCT GGA GAA GCT TCG GC
ABO-T5 (C-A)-F	GCC GAA GCT TCT CCA GGT GGT CGC GGA TCT CCA TGC TTC TAG AGG CC
ABO-T5 (C-A)-R	GGC CTC TAG AAG CAT GGA GAT CCG CGA CCA CCT GGA GAA GCT TCG GC
ABO-T5 (T-G)-F	GCC GAA GCT TCT CCA AGT GGT CGC GGA CCT CCA TGC TTC TAG AGG CC
ABO-T5 (T-G)-R	GGC CTC TAG AAG CAT GGA GGT CCG CGA CCA CTT GGA GAA GCT TCG GC
ABO-T5 (T-C)-F	GCC GAA GCT TCT CCA AGT GGT CGC GGA GCT CCA TGC TTC TAG AGG CC
ABO-T5 (T-C)-R	GGC CTC TAG AAG CAT GGA GCT CCG CGA CCA CTT GGA GAA GCT TCG GC
ABO-T5 (T-T)-F	GCC GAA GCT TCT CCA AGT GGT CGC GGA ACT CCA TGC TTC TAG AGG CC
ABO-T5 (T-T)-R	GGC CTC TAG AAG CAT GGA GTT CCG CGA CCA CTT GGA GAA GCT TCG GC
ABO-T5 (T-A)-F	GCC GAA GCT TCT CCA AGT GGT CGC GGA TCT CCA TGC TTC TAG AGG CC
ABO-T5 (T-A)-R	GGC CTC TAG AAG CAT GGA GAT CCG CGA CCA CTT GGA GAA GCT TCG GC
ABO-T5 (A-G)-F	GCC GAA GCT TCT CCA TGT GGT CGC GGA CCT CCA TGC TTC TAG AGG CC
ABO-T5 (A-G)-R	GGC CTC TAG AAG CAT GGA GGT CCG CGA CCA CAT GGA GAA GCT TCG GC
ABO-T5 (A-C)-F	GCC GAA GCT TCT CCA TGT GGT CGC GGA GCT CCA TGC TTC TAG AGG CC
ABO-T5 (A-C)-R	GGC CTC TAG AAG CAT GGA GCT CCG CGA CCA CAT GGA GAA GCT TCG GC
ABO-T5 (A-T)-F	GCC GAA GCT TCT CCA TGT GGT CGC GGA ACT CCA TGC TTC TAG AGG CC

ABO-T5 (A-T)-R	GGC CTC TAG AAG CATGGAGTTCCGCGACCACATGGA GAA GCT TCG GC
ABO-T5 (A-A)-F	GCC GAA GCT TCT CCATGTGGT CGC GGA TCT CCA TGC TTC TAG AGG CC
ABO-T5 (A-A)-R	GGC CTC TAG AAG CATGGAGATCCGCGACCACATGGA GAA GCT TCG GC
ABO-T7 (G-G)-F	GCC GAA GCT TCT CCA CCT GGT CGC GGA CCT CCA TGC TTC TAG AGG CC
ABO-T7 (G-G)-R	GGC CTC TAG AAG CATGGAGGTCCGCGACCAGGTGGA GAA GCT TCG GC
ABO-T7 (G-C)-F	GCC GAA GCT TCT CCA CCT GGT CGC GGA GCT CCA TGC TTC TAG AGG CC
ABO-T7 (G-C)-R	GGC CTC TAG AAG CATGGAGCTCCGCGACCAGGTGGA GAA GCT TCG GC
ABO-T7 (G-T)-F	GCC GAA GCT TCT CCA CCT GGT CGC GGA ACT CCA TGC TTC TAG AGG CC
ABO-T7 (G-T)-R	GGC CTC TAG AAG CATGGAGTTCCGCGACCAGGTGGA GAA GCT TCG GC
ABO-T7 (G-A)-F	GCC GAA GCT TCT CCA CCT GGT CGC GGA TCT CCA TGC TTC TAG AGG CC
ABO-T7 (G-A)-R	GGC CTC TAG AAG CATGGAGATCCGCGACCAGGTGGA GAA GCT TCG GC
ABO-T7 (C-G)-F	GCC GAA GCT TCT CCA CGT GGT CGC GGA CCT CCA TGC TTC TAG AGG CC
ABO-T7 (C-G)-R	GGC CTC TAG AAG CATGGAGGTCCGCGACCACGTGGA GAA GCT TCG GC
ABO-T7 (C-C)-F	GCC GAA GCT TCT CCA CGT GGT CGC GGA GCT CCA TGC TTC TAG AGG CC
ABO-T7 (C-C)-R	GGC CTC TAG AAG CATGGAGCTCCGCGACCACGTGGA GAA GCT TCG GC
ABO-T7 (C-T)-F	GCC GAA GCT TCT CCA CGT GGT CGC GGA ACT CCA TGC TTC TAG AGG CC
ABO-T7 (C-T)-R	GGC CTC TAG AAG CAT GGA GTT CCG CGA CCA CGT GGA GAA GCT TCG GC
ABO-T7 (C-A)-F	GCC GAA GCT TCT CCA CGT GGT CGC GGA TCT CCA TGC TTC TAG AGG CC
ABO-T7 (C-A)-R	GGC CTC TAG AAG CATGGAGATCCGCGACCACGTGGA GAA GCT TCG GC
ABO-T7 (T-G)-F	GCC GAA GCT TCT CCA CAT GGT CGC GGA CCT CCA TGC TTC TAG AGG CC
ABO-T7 (T-G)-R	GGC CTC TAG AAG CATGGAGGTCCGCGACCATGTGGA GAA GCT TCG GC
ABO-T7 (T-C)-F	GCC GAA GCT TCT CCA CAT GGT CGC GGA GCT CCA TGC TTC TAG AGG CC

ABO-T7 (T-C)-R	GGC CTC TAG AAG CATGGAGCTCCGCGACCATGTGGA GAA GCT TCG GC
ABO-T7 (T-T)-F	GCC GAA GCT TCT CCA CAT GGT CGC GGA ACT CCA TGC TTC TAG AGG CC
ABO-T7 (T-T)-R	GGC CTC TAG AAG CATGGAGTTCCGCGACCATGTGGA GAA GCT TCG GC
ABO-T7 (T-A)-F	GCC GAA GCT TCT CCA CAT GGT CGC GGA TCT CCA TGC TTC TAG AGG CC
ABO-T7 (T-A)-R	GGC CTC TAG AAG CATGGAGATCCGCGACCATGTGGA GAA GCT TCG GC
ABO-T7 (A-G)-F	GCC GAA GCT TCT CCA CTT GGT CGC GGA CCT CCA TGC TTC TAG AGG CC
ABO-T7 (A-G)-R	GGC CTC TAG AAG CATGGAGGTCCGCGACCAAGTGGA GAA GCT TCG GC
ABO-T7 (A-C)-F	GCC GAA GCT TCT CCA CTT GGT CGC GGA GCT CCA TGC TTC TAG AGG CC
ABO-T7 (A-C)-R	GGC CTC TAG AAG CATGGAGCTCCGCGACCAAGTGGA GAA GCT TCG GC
ABO-T7 (A-T)-F	GCC GAA GCT TCT CCA CTT GGT CGC GGA ACT CCA TGC TTC TAG AGG CC
ABO-T7 (A-T)-R	GGC CTC TAG AAG CATGGAGTTCCGCGACCAAGTGGA GAA GCT TCG GC
ABO-T7 (A-A)-F	GCC GAA GCT TCT CCA CTT GGT CGC GGA TCT CCA TGC TTC TAG AGG CC
ABO-T7 (A-A)-R	GGC CTC TAG AAG CATGGAGATCCGCGACCAAGTGGA GAA GCT TCG GC
HIV-T1-F	GCC GAA GCT TCT TTTGATAAAACCTCCAATTCCCCCTATC TTC TAG AGG CC
HIV-T1-R	GGC CTC TAG AAG ATAGGGGGAATTGGAGGTTTTATCAAAA GAA GCT TCG GC
HIV-T2-F	GCC GAA GCT TCT TTTGATAAGACCTCCAATTCCCCCTATC TTC TAG AGG CC
HIV-T2-R	GGC CTC TAG AAG ATAGGGGGAATTGGAGGTCTTATCAAA A GAA GCT TCG GC
HIV-T3-F	GCC GAA GCT TCT TTTGATAAAACCTCCAATTCCCACTATC TTC TAG AGG CC
HIV-T3-R	GGC CTC TAG AAG ATAGTGGGAATTGGAGGTTTTATCAAAA GAA GCT TCG GC
HIV-T4-F	GCC GAA GCT TCT TTTGATAAAACCTCCAATTCCCCCTACC TTC TAG AGG CC
HIV-T4-R	GGC CTC TAG AAG GTAGGGGGAATTGGAGGTTTTATCAAA A GAA GCT TCG GC
HIV-T5-F	GCC GAA GCT TCT TTTGATAAGACCTCCAATTCCCACTATC TTC TAG AGG CC

HIV-T5-R	GGC CTC TAG AAG ATAGTGGGAATTGGAGGTCTTATCAAAA GAA GCT TCG GC
HIV-T6-F	GCC GAA GCT TCT TTTGATAAGACCTCCAATTCCCCCTACC TTC TAG AGG CC
HIV-T6-R	GGC CTC TAG AAG GTAGGGGGAATTGGAGGTCTTATCAAA A GAA GCT TCG GC
HIV-T7-F	GCC GAA GCT TCT TTTGATAAAACCTCCAATTCCCACTACC TTC TAG AGG CC
HIV-T7-R	GGC CTC TAG AAG GTAGTGGGAATTGGAGGTTTTATCAAAA GAA GCT TCG GC
HIV-T8-F	GCC GAA GCT TCT TTTGATAAGACCTCCAATTCCCACTACC TTC TAG AGG CC
HIV-T8-R	GGC CTC TAG AAG GTAGTGGGAATTGGAGGTCTTATCAAA A GAA GCT TCG GC
Negative-F	GCC GAA GCT TCT TTTGATTCTTGCTCTGCTCTCTTCGTCC TTC TAG AGG CC
Negative-R	GGC CTC TAG AAG GACGAAGAGAGCAGAGCAAGAATCAAA A GAA GCT TCG GC
pUC19_F	CAGCGAGTCAGTGAGCGA
pUC19_R	GCGACACGGAAATGTTGAATACTCAT
HLA-C-F	ACACACTCGAAACGTCCCAA
HLA-C-R	AAGTCCTTCTGGAGCCCTTC

Name	Sequences (5′→3′)
Oligos used in *in vitro* high throughput assay experiments.	
HLA-library	/5′Phos/*TTGTGT*NNNNC*C*NG*G*G*C*C*T*T*G*T*A*G*A*T *C*T*G*T*G*T*G*NNNNACCTGCCGAG*TTGTGT*
ABO-library	/5′Phos/*AGAGAA*NNNNC*C*NC*G*T*G*G*T*C*G*C*G*G*A* A*C*T*C*C*A*T*G*NNNNACCTGCCGAG*AGAGAA*
S501-F	AATGATACGGCGACCACCGAGATCTACACTCTTTCCCTA CACGACGCTCTTCCGATCT***TAGATCGC***
S501-R	GCGATCTAAGATCGGAAGAGCGTCGTGTAGGGAAAGAG TGTAGATCTCGGTGG
S502-F	AATGATACGGCGACCACCGAGATCTACACTCTTTCCCTA CACGACGCTCTTCCGATCT*C****TCTCTAT***
S502-R	ATAGAGAGAGATCGGAAGAGCGTCGTGTAGGGAAAGAG TGTAGATCTCGGTGG
S503-F	AATGATACGGCGACCACCGAGATCTACACTCTTTCCCTA CACGACGCTCTTCCGATCT***TATCCTCT***
S503-R	AGAGGATAAGATCGGAAGAGCGTCGTGTAGGGAAAGAG TGTAGATCTCGGTGG

S504-F	AATGATACGGCGACCACCGAGATCTACACTCTTTCCCTA CACGACGCTCTTCCGATCT***AGAGTAGA***
S504-R	TCTACTCTAGATCGGAAGAGCGTCGTGTAGGGAAAGAGT GTAGATCTCGGTGG
S505-F	AATGATACGGCGACCACCGAGATCTACACTCTTTCCCTA CACGACGCTCTTCCGATCT***GTAAGGAG***
S505-R	CTCCTTACAGATCGGAAGAGCGTCGTGTAGGGAAAGAGT GTAGATCTCGGTGG
S506-F	AATGATACGGCGACCACCGAGATCTACACTCTTTCCCTA CACGACGCTCTTCCGATCT***ACTGCATA***
S506-R	TATGCAGTAGATCGGAAGAGCGTCGTGTAGGGAAAGAG TGTAGATCTCGGTGG
S507-F	AATGATACGGCGACCACCGAGATCTACACTCTTTCCCTA CACGACGCTCTTCCGATCT***AAGGAGTA***
S507-R	TACTCCTTAGATCGGAAGAGCGTCGTGTAGGGAAAGAGT GTAGATCTCGGTGG
S508-F	AATGATACGGCGACCACCGAGATCTACACTCTTTCCCTA CACGACGCTCTTCCGATCT***CTAAGCCT***
S508-R	AGGCTTAGAGATCGGAAGAGCGTCGTGTAGGGAAAGAG TGTAGATCTCGGTGG
HLA-N701	CAAGCAGAAGACGGCATACGAGAT***TCGCCTTA***ACCTGCC GAG*TTGTGT*
HLA-N702	CAAGCAGAAGACGGCATACGAGAT***CGTACTAG***ACCTGC CGAG*TTGTGT*
HLA-N703	CAAGCAGAAGACGGCATACGAGAT***TTCTGCCT***ACCTGCC GAG*TTGTGT*
ABO-N705	CAAGCAGAAGACGGCATACGAGAT***AGGAGTCC***ACCTGC CGAG*AGAGAA*
ABO-N706	CAAGCAGAAGACGGCATACGAGAT***CATGCCTA***ACCTGC CGAG*AGAGAA*
ABO-N707	CAAGCAGAAGACGGCATACGAGAT***GTAGAGAG***ACCTGC CGAG*AGAGAA*
ABO-N708	CAAGCAGAAGACGGCATACGAGAT***CCTCTCTG***ACCTGCC GAG*AGAGAA*
PE2_short	AAT GAT ACG GCG ACC ACC GA
HLA_sel_PCR	CAA GCA GAA GAC GGC ATA CGA GAT ACC TGC CGA G*TT GTG T*
ABO_sel_PCR	CAA GCA GAA GAC GGC ATA CGA GAT ACC TGC CGA G*AG AGA A*
Lib_adaptor1	GAC GGC ATA CGA GAT
HLA_lib_adaptor2	*TTG T*AT CTC GTA TGC CGT CTT CTG CTT G
ABO_lib_adaptor2	*AGA G*AT CTC GTA TGC CGT CTT CTG CTT G
lib_PCR_F	CAA GCA GAA GAC GGC ATA CGA GAT
HLA_PCR_R	AAT GAT ACG GCG ACC ACC GAG ATC TAC ACT CTT TCC CTA CAC GAC GCT CTT CCG ATC TNN NNA CCT ACC TGC CGA G*TT GTG T*

ABO_PCR_R	AAT GAT ACG GCG ACC ACC GAG ATC TAC ACT CTT TCC CTA CAC GAC GCT CTT CCG ATC TNN NNA CCT ACC TGC CGA G*AG AGA A*

NNNNNNN = Library barcode, ***NNNNNNNN*** = target barcode
An asterisk (*) indicates that the preceding nucleotide was incorporated as a hand mix of bases consisting of 79 mol % of the indicated base, and 7 mol % of each of the other three natural bases. "/5Phos/" denotes a 5′ phosphate group added to the sequence.

Name	Sequences (5′→3′)
Oligos used to create DNA target constructs in a cell reporter assay.	
ABO-T1-REP-F	AATTCC CATGGAGTTCCGCGACCACGTGG AGGAG
ABO-T1-REP-R	GATCCTCCT CCACGTGGTCGCGGAACTCCATG GG
ABO-T2-REP-F	AATTCC CATGGAGATCCGCGACCACGTGG AGGAG
ABO-T2-REP-R	GATCCTCCT CCACGTGGTCGCGGATCTCCATG GG
ABO-T3-REP-F	AATTCC CATGGAGTTCCGCGACCATGTGG AGGAG
ABO-T3-REP-R	GATCCTCCT CCACATGGTCGCGGAACTCCATG GG
ABO-T4-REP-F	AATTCC CATGGAGTTCCGCGACCACATGG AGGAG
ABO-T4-REP-R	GATCCTCCT CCATGTGGTCGCGGAACTCCATG GG
ABO-T5-REP-F	AATTCC CATGGAGATCCGCGACCACATGG AGGAG
ABO-T5-REP-R	GATCCTCCT CCATGTGGTCGCGGATCTCCATG GG
ABO-T6-REP-F	AATTCC CATGGAGATCCGCGACCATATGG AGGAG
ABO-T6-REP-R	GATCCTCCT CCATATGGTCGCGGATCTCCATG GG
ABO-T7-REP-F	AATTCC CATGGAGATCCGCGACCATGTGG AGGAG
ABO-T7-REP-R	GATCCTCCT CCACATGGTCGCGGATCTCCATG GG
ABO-T8-REP-F	AATTCC CATGGAGTTCCGCGACCATATGG AGGAG
ABO-T8-REP-R	GATCCTCCT CCATATGGTCGCGGAACTCCATG GG

Name	Sequences (5′→3′)
Oligo used as ssDNA target in melting temperature assay experiments.	
ABO-T1-Tm	CCCACGTGGTCGCGGAACTCCATGT

Name	Sequences (5′→3′)
Oligos used for dsDNA target in EMSA assay experiments.	
ABO-T1-EMSA-F	CAATA CCACGTGGTCGCGGAACTCCATG
ABO-T1-EMSA-R	CATGGAGTTCCGCGACCACGTGG TATTGCGC/Cy5/
ABO-T5-EMSA-F	CAATA CCATGTGGTCGCGGATCTCCATG
ABO-T5-EMSA-R	CATGGAGATCCGCGACCACATGG TATTGCGC/Cy5/

ABO-T7-EMSA-F	CAATA CCACATGGTCGCGGATCTCCATG
ABO-T7-EMSA-R	CATGGAGATCCGCGACCATGTGG TATTGCGC/Cy5/
ABO-Neg-EMSA-F	CAATA CCTGTGCCTTGTAGATCTGTGTG
ABO-Neg-EMSA-R	CACACAGATCTACAAGGCACAGG TATTGCGC/Cy5/

/Cy5/ refers to Cyanine 5.

Name	Sequences (5′→3′)
Oligos used in cellular cleavage assay experiments.	
HLA-C-F	ACACACTCGAAACGTCCCAA
HLA-C-R	AAGTCCTTCTGGAGCCCTTC
HIV-T1-Flp-F	GATCCCGAGTGAAGATGGAAACCAAAAATGATAGGGGG AATTGGAGGTTTTATCAAAGTAAGACAGTATGATCAGATC GGCCGC
HIV-T1-Flp-R	TCGAGCGGCCGATCTGATCATACTGTCTTACTTTGATAAA ACCTCCAATTCCCCCTATCATTTTTGGTTTCCATCTTCACT CGG
HIV-T8-Flp-F	GATCCCGAGTGAAGATGGAAACCAAAAATGGTAGTGGGA ATTGGAGGTCTTATCAAAGTAAGACAGTATGATCAGATC GGCCGC
HIV-T8-Flp-R	TCGAGCGGCCGATCTGATCATACTGTCTTACTTTGATAAG ACCTCCAATTCCCACTACCATTTTTGGTTTCCATCTTCAC TCGG
FlpLocus-F	CGATGTACGGGCCAGATATAC
FlpLocus-R	AGGGAAGAAAGCGAAAGGAG
HIV-OT1-F	CGTGTACACACCTTCGTTGC
HIV-OT1-R	TCCCGACTGCCTAAGATGGA
HIV-OT2-F	GCTCTCTTGCCCATGGAGTT
HIV-OT2-R	AATTGGGGCCTTGAGACCAG

REFERENCES

1 Bae, S., Park, J. & Kim, J. S. Cas-OFFinder: a fast and versatile algorithm that searches for potential off-target sites of Cas9 RNA-guided endonucleases. *Bioinformatics* **30**, 1473-1475, doi:10.1093/bioinformatics/btu048 (2014).
