# Supplementary material for: Guide RNAs containing universal bases enable Cas9/Cas12a recognition of polymorphic sequences
Source: Nat Commun. 2022 Mar 25;13:1617. doi: 10.1038/s41467-022-29202-x (PMC8956631; doi:10.1038/s41467-022-29202-x)
Supplement: Supplementary file 2 — Reporting Summary [file 41467_2022_29202_MOESM2_ESM.pdf]

nature portfolio

Corresponding author(s): Basil P. Hubbard

Last updated by author(s): Feb 21, 2022

Reporting Summary

Nature Portfolio wishes to improve the reproducibility of the work that we publish. This form provides structure for consistency and transparency in reporting. For further information on Nature Portfolio policies, see our Editorial Policies and the Editorial Policy Checklist.

Statistics

For all statistical analyses, confirm that the following items are present in the figure legend, table legend, main text, or Methods section.

n/a	Confirmed	
☐	☒	The exact sample size (n) for each experimental group/condition, given as a discrete number and unit of measurement
☐	☒	A statement on whether measurements were taken from distinct samples or whether the same sample was measured repeatedly
☒	☐	The statistical test(s) used AND whether they are one- or two-sided<br>*Only common tests should be described solely by name; describe more complex techniques in the Methods section.*
☒	☐	A description of all covariates tested
☒	☐	A description of any assumptions or corrections, such as tests of normality and adjustment for multiple comparisons
☐	☒	A full description of the statistical parameters including central tendency (e.g. means) or other basic estimates (e.g. regression coefficient) AND variation (e.g. standard deviation) or associated estimates of uncertainty (e.g. confidence intervals)
☒	☐	For null hypothesis testing, the test statistic (e.g. F, t, r) with confidence intervals, effect sizes, degrees of freedom and P value noted<br>*Give P values as exact values whenever suitable.*
☒	☐	For Bayesian analysis, information on the choice of priors and Markov chain Monte Carlo settings
☒	☐	For hierarchical and complex designs, identification of the appropriate level for tests and full reporting of outcomes
☒	☐	Estimates of effect sizes (e.g. Cohen's d, Pearson's r), indicating how they were calculated

Our web collection on statistics for biologists contains articles on many of the points above.

Software and code

Policy information about availability of computer code

Data collection	No software was used for data collection.
Data analysis	Custom python scripts are used for data analysis of high-throughput profiling data. These have been previously published (Cromwell et al., Nature Comm, 2018). Densitometry was performed using ImageJ (v 1.53a).

For manuscripts utilizing custom algorithms or software that are central to the research but not yet described in published literature, software must be made available to editors and reviewers. We strongly encourage code deposition in a community repository (e.g. GitHub). See the Nature Portfolio guidelines for submitting code & software for further information.

Data

Policy information about availability of data

All manuscripts must include a data availability statement. This statement should provide the following information, where applicable:

- Accession codes, unique identifiers, or web links for publicly available datasets
- A description of any restrictions on data availability
- For clinical datasets or third party data, please ensure that the statement adheres to our policy

Data associated with this manuscript are included in this published article and its associated Supplementary Information files; unprocessed data outputs are found in the Source Data file. All high-throughput sequencing data files associated with this manuscript have been deposited in the NCBI SRA database and are available under accession number: PRJNA669024. Cellular targets were created based on genomes from Ensembl (https://uswest.ensembl.org/index.html) and HEK293 (http://www.hek293genome.org/v1/index.php) databases. This information is indicated in the 'Data availability' section of the manuscript.

Field-specific reporting

Please select the one below that is the best fit for your research. If you are not sure, read the appropriate sections before making your selection.

- [x] Life sciences
- [ ] Behavioural & social sciences
- [ ] Ecological, evolutionary & environmental sciences

For a reference copy of the document with all sections, see nature.com/documents/nr-reporting-summary-flat.pdf

Life sciences study design

All studies must disclose on these points even when the disclosure is negative.

Sample size	High-throughput experiments utilized libraries of ~10^12 unique DNA oligonucleotide sequences. Shown high-throughput analysis is representative of all sequences in the final samples. For all other data points, experiments were performed in duplicate (unless otherwise indicated in the figure legend).
Data exclusions	N/A. All data is presented.
Replication	Experiments were performed twice in independent replicates (unless otherwise stated). All replication attempts were successful for the given data.
Randomization	Randomization is not relevant in this study. High-throughput experiments utilized libraries of ~10^12 partially randomized sequences and can be described as a physical equivalent to experiment randomization.
Blinding	As no human subjects were used in this study, blinding was not relevant. Data was processed as described within the manuscript, without data being omitted. Data analysis was not biased by the performing researcher's input.

Reporting for specific materials, systems and methods

We require information from authors about some types of materials, experimental systems and methods used in many studies. Here, indicate whether each material, system or method listed is relevant to your study. If you are not sure if a list item applies to your research, read the appropriate section before selecting a response.

Materials & experimental systems

n/a	Involved in the study	
[x]	[ ]	Antibodies
[ ]	[x]	Eukaryotic cell lines
[x]	[ ]	Palaeontology and archaeology
[x]	[ ]	Animals and other organisms
[x]	[ ]	Human research participants
[x]	[ ]	Clinical data
[x]	[ ]	Dual use research of concern

Methods

n/a	Involved in the study	
[x]	[ ]	ChIP-seq
[x]	[ ]	Flow cytometry
[x]	[ ]	MRI-based neuroimaging

Eukaryotic cell lines

Policy information about cell lines

Cell line source(s)	HeLa and 293T cells (previously used and obtained from ATCC), 293-Flp In Cells (bought for this work from ThermoFisher).
Authentication	Cell lines were authenticated by vendors prior to purchase, no internal genetic authentication was performed.
Mycoplasma contamination	As stated in the manuscript, all cell lines used were tested for mycoplasma at the time of use and were negative.
Commonly misidentified lines (See ICLAC register)	HEK derivative (293T, Flp-In 293) cells were used in this study. However cell lines were authenticated by vendors prior to purchase. No internal genetic authentication was performed.
